# Synthesis of α-(perfluoroalkylsulfonyl)propiophenones: a new set of reagents for the light-mediated perfluoroalkylation of aromatics

**DOI:** 10.3762/bjoc.18.79

**Published:** 2022-07-04

**Authors:** Durbis J Castillo-Pazos, Juan D Lasso, Chao-Jun Li

**Affiliations:** 1 Department of Chemistry and FRQNT Centre for Green Chemistry and Catalysis, McGill University, 801 Sherbrooke Street W, Montreal, Quebec H3A 0B8, Canada

**Keywords:** α-(perfluoroalkylsulfonyl)propiophenones, innate functionalization, late-stage functionalization, light-mediated perfluoroalkylation, perfluoroalkyl sulfinates

## Abstract

In response to the demand for late-stage perfluoroalkylation in synthetic chemistry, we report the synthesis of a series of bench-stable α-(perfluoroalkylsulfonyl)propiophenones. Their application as photocleavable reagents was tested with electron-rich aromatics under metal-free, redox- and pH-neutral conditions to enable late-stage perfluorooctylation, perfluorohexylation, and perfluorobutylation.

## Introduction

Perfluorinated compounds are a family of molecules containing a backbone where all C–H bonds have been substituted with fluorine atoms. Within this family of molecules, perfluoroalkyl groups represent an industrially relevant moiety, capable of modifying the physicochemical properties of the scaffold that they are attached to. Such properties and a distinctive reactivity – or inert character – have been harnessed in a plethora of applications in modern life: Teflon in non-stick pans, fire-fighting foams, stain-resistant and weatherproof fabrics, etching of circuit boards, and even imaging agents [1]. Given their importance, multiple synthetic methodologies for the introduction of long perfluorinated chains into aromatic rings have been developed since the first reports of such transformation by George Tiers in 1960, and McLoughlin and Thrower in 1969 [2–3]. Most approaches have made extensive use of organometallic chemistry, radical initiators, photocatalysis, electrochemistry, and more sophisticated platforms such as metal nanoparticles, all of which have been reviewed thoroughly in the literature [4–8].

However, the methods referenced so far display one or more of the following setbacks: involvement of harsh oxidants or reductants, use of expensive metal catalysts, need for superstoichiometric amounts of starting materials, generation of undesired perfluoroalkylated byproducts, and poor chemoselectivity. Modern perfluoralkylation methodologies exceedingly rely on the use of perfluoroalkyl iodides as their principal source of perfluoroalkyl synthons. Indeed, while these molecules are cheap and abundant starting materials their use is fraught with technical complications. This family of molecules is extremely sensitive to bench conditions and requires a carefully controlled refrigeration in addition to low light levels to avoid decomposition. Furthermore, true to the unique solubility of this class of molecules, perfluoroalkyl iodides have a tendency to be weakly soluble in common organic solvents (i.e., ethyl acetate and methanol) rendering their application troublesome [9]. Moreover, the homolysis of the perfluoroalkyl iodide produces iodine radicals that can result in stray halogenation reactions or oxidation. For these reasons, it would be ideal to develop an efficient methodology that allows for the generation of perfluoroalkyl radicals in a mild, redox- and pH-neutral manner, without the assistance of external photocatalysts, heavy metal catalysts, or further additives. Thus, the expansion of our previously reported propiophenone family of reagents was envisioned as suitable alternative to produce a bench stable, organic soluble, and iodine-free perfluroalkylation source.

In 2017, our group developed a metal-free and redox-neutral protocol for the photoinduced alkylation of aromatics, for which trifluoromethylation was also possible in good to high yields for electron-rich aromatic rings [10]. In this protocol, inspired by Norrish type I reactions and the elimination of β-substituents after ketone photoexcitation [11–13], a series of reagents containing an α-sulfonylpropiophenone moiety readily undergoes homolysis into three parts upon irradiation of light: a propiophenone radical – forming a stabilized and bulky “dummy group” –, a molecule of SO_2_, and our radical of interest. Once this radical is formed in solution, radical addition to the aromatic substrate undergoes readily, and is subsequently followed by a hydrogen atom transfer (HAT) process aided by the “dummy group” radical. These reagents thus fit the paradigm of a green methodology as their implicit design and photoactivity allows them to react without the use of external metal catalysts. The intrinsic reactivity of these molecules allows this set of reagents to be both redox- and pH-neutral, while also being highly diversifiable. Additionally, all byproducts generated either during its synthesis or use in following reactions have the potential to be recycled, if so desired.

**Scheme 1:**
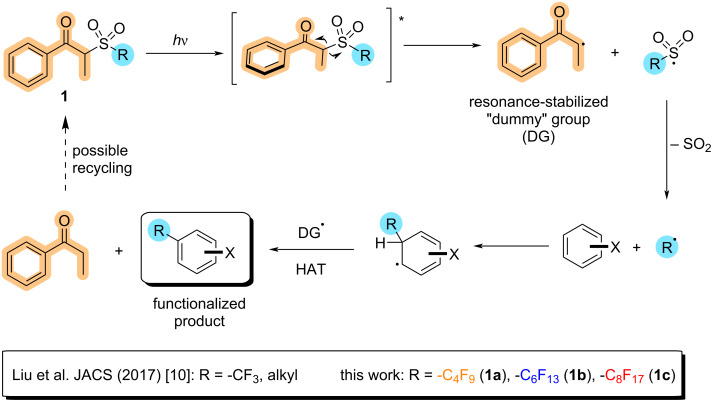
Envisioned Minisci perfluoroalkylation facilitated by “dummy group” reagents **1a**–**c**.

Based on the fact that both, trifluoromethyl radicals and its longer-chain analogues, share a common electrophilic character and a stabilizing stereoelectronic effect [14], we envisioned that the “dummy group” methodology could be translated into the formation of sought after perfluoroalkyl radicals (Scheme 1). In this work, we report the synthesis and application of three new members of the “dummy group” reagent family, based on the α-(perfluoroalkylsulfonyl)propiophenone scaffold for the perfluorobutylation (**1a**), perfluorohexylation (**1b**) and perfluorooctylation (**1c**) of electron-rich aromatics (Scheme 1). With the insights discussed in this paper, the authors hope to provide a new and amenable synthetic tool for the future academic and industrial demand of perfluorinated molecules and materials.

## Results and Discussion

To design an efficient and reproducible methodology for the synthesis of α-(perfluoroalkylsulfonyl)propiophenones, we envisioned the bimolecular nucleophilic substitution between an α-halopropiophenone as the “dummy group” scaffold and the corresponding perfluorinated sodium sulfinate salt – also visualized as the installation of the photocleavable moiety onto the perfluoroalkyl chain [15–19]. The precursory sulfinate salts **2a**–**c** were synthesized through the sulfinatodehalogenation reaction, discovered by Huang and co-workers [20–21], and later on adapted by other research groups [22–23]. Pleasingly, the desired C_4_F_9_- (**2a**), C_6_F_13_- (**2b**), and C_8_F_17_- (**2c**) sulfinate salts were obtained from the perfluoroalkyl iodide precursors in good to quantitative yields as previously described. Additionally, we conceived that this methodology should be amenable to the synthesis of one of the limited commercially available secondary perfluoroalkyl groups such as perfluoroisopropyl iodide. However, despite being able to obtain the corresponding sulfinate in limited yield, the decomposition of this compound after several days at 4 °C, and within a few minutes under heating deemed its applicability impractical.

**Scheme 2:**
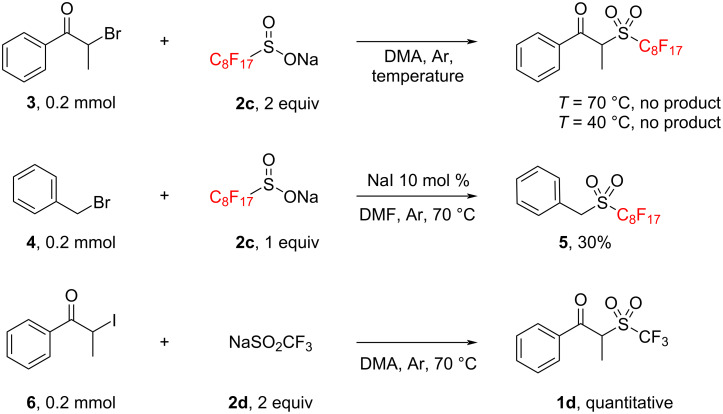
Control experiments for the nucleophilic substitution of perfluoroalkylsulfinates **2** and halogenated electrophilic partners.

After this first step, we proceeded to test the nucleophilic substitution between our perfluoroalkylsulfinate salts **2** and an α-halopropiophenone. Unfortunately, initial attempts of a nucleophilic attack of sodium perfluorooctylsulfinate (**2c**) on α-bromopropiophenone (**3**) were unsuccessful to produce the desired product **1c** due to the insufficient nucleophilicity of the sulfinate to substitute a bromide on a secondary carbon atom at 40 °C (Scheme 2).

Furthermore, increasing the temperature to 70 °C was not found to generate the product and instead resulted in slight decomposition of the starting materials. Attempting to trap the sulfinate nucleophiles with primary benzyl bromide (**4**) with catalytic sodium iodide under thermal conditions afforded product **5** in only 30% yield and underscored the sluggish reactivity of these sulfinate derivatives towards undergoing nucleophilic substitution (Scheme 2). To solve this problem, we turned to the use of α-iodopropiophenone (**6**), generated from its bromide counterpart through a simple Finkelstein reaction [24]. After performing a control experiment between α-iodopropiophenone (**6**) and sodium triflinate (**2d**) that afforded a quantitative yield of **1d**, we proceeded to optimize the conditions for the nucleophilic substitution on this substrate by sodium perfluorohexylsulfinate (**2b**) to synthesize α-(perfluorohexyl)propiophenone (**1b**, Table 1). Temperature displayed a pivotal role in this synthesis: while room temperature proved insufficient to promote the substitution, the use of heat beyond 70 °C was detrimental for the reaction due to decomposition of the product. Once established that 40 °C was enough to promote the reaction, while limiting decomposition, we proceeded to screen the possible molar ratios between both components in the reaction. Given the higher economic value of the perfluorinated salts **2**, we decided to vary the amounts of α-iodopropiophenone **6** to increase the molar ratio. Ranging from a 2.5:1 until a 10:1 molar ratio, yields increased significantly from 20% to 68%; however, a 5:1 ratio offered us a similar yield with a much shorter workup time when the reaction concentration was doubled.

**Table 1:** Optimization for the nucleophilic substitution between α-iodopropiophenone (**6**) and sodium perfluorohexylsulfinate (**2b**).

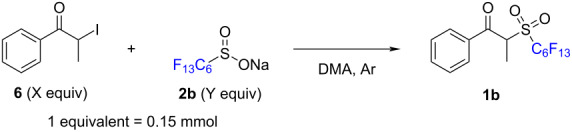					

Entry	Molar ratio **6**/**2b** X:Y	Volume DMA (mL)	Temperature (°C)	Time (h)	NMR yield **1b** (%)^a^

1	1:1.5	1	**70**	16	n.d.
2	1:1.5	1	**40**	16	18
3	1:1.5	1	**20**	16	traces
4	**2.5:1**	0.5	40	18	20
5	**4:1**	0.5	40	18	33
6	**10:1**	0.5	40	18	68
7	**40:1**	0.5	40	18	48
8	**3:1**	0.5	**50**	18	12
9	**4:1**	0.5	**50**	18	19
10	**5:1**	**1**	40	18	24
11	5:1	**0.5**	40	18	38
12	5:1	**0.25**	40	18	**51**
13	5:1	**0.125**	40	18	53

^a^Using dimethylsulfone as a standard.

Knowing that nucleophilicity is a key factor in this reaction, we also employed crown ethers, 15-crown-5 and 18-crown-6, to test whether a “naked” sulfinate ion would help us achieve a better yield. Unfortunately, the addition of such ethers shut down all reactivity, most likely due to side reactions with the sulfinate salt. Moreover, it is worth mentioning that, while other synthetic approaches were explored to obtain these reagents, the S_N_2 strategy described in this work was the most efficient. Such synthetic alternatives included: first, a sulfur(VI) fluoride exchange (SuFEx) between perfluoroalkylsulfonyl fluorides and the corresponding silyl enol ether generated in situ from propiophenone, and second, the deprotection of propiophenone α-thioesters in the presence of perfluoroalkyliodides and subsequent oxidation of the formed perfluorothioether into the sulfone. However, none of these proposed pathways gave yields high enough for the reaction to be scalable (i.e., a maximum of 15% by ^1^H NMR).

**Scheme 3:**
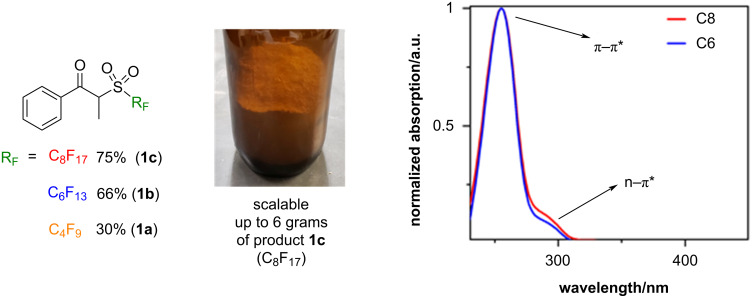
Left: isolated yields of synthesized perfluoroalkylating reagents: perfluorobutyl (**1a**), perfluorohexyl (**1b**), and perfluorooctyl (**1c**) analogues (after conversion of byproduct); middle: gram amounts of perfluorooctyl product **1c**; right: UV–vis absorption of reagents **1b** and **1c**.

Finally, due to the concentration of α-iodopropiophenone (**6**) employed, we detected the formation of a byproduct in the last stages of the optimization, namely the condensation of the desired product with α-propiophenone in the form of an enol ether. Once this byproduct was fully characterized by NMR, and the structure was confirmed by SCXRD, we conceived a hydrolysis protocol to break apart the formed enol ether (fully described in section 2.4 of Supporting Information File 1). After brief optimization, we succeeded at recovering the portion of perfluoroalkylating reagent that participated in such condensation (around 30%), giving us the final yields of perfluoroalkylating reagents **1a**–**c** displayed in Scheme 3. Afterward, to show the practicality of application of these reagents in industry, we proceeded to scale up their synthesis in gram-scale. Satisfactorily, the developed synthesis and workup allowed us to produce the desired products in batches of up to six grams, with no decomposition observed over the course of 6 months.

**Scheme 4:**
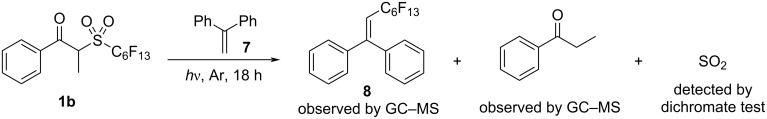
Radical trapping experiment with 1,1-diphenylethylene (**7**) and **1b** confirming the initially proposed mechanism.

For the last section of this work, we proceeded to test the capacity of our reagents to generate the desired perfluoroalkyl radicals under light irradiation for the diversification of arenes. To verify the generation of perfluoroalkyl radicals from compounds **1**, we conducted an experiment with perfluorohexyl analogue **1b** and 1,1-diphenylethylene (**7**) as a radical trapping agent (Scheme 4). Gratifyingly we observed the formation of 2-(perfluorohexyl)-1,1-diphenylethylene (**8**), and propiophenone through GC–MS analysis. Additionally, the presence of free SO_2_ gas was confirmed by the reaction of acidic potassium dichromate solution on paper (green coloration of the exposed surface). See Supporting Information File 1 for details.

**Scheme 5:**
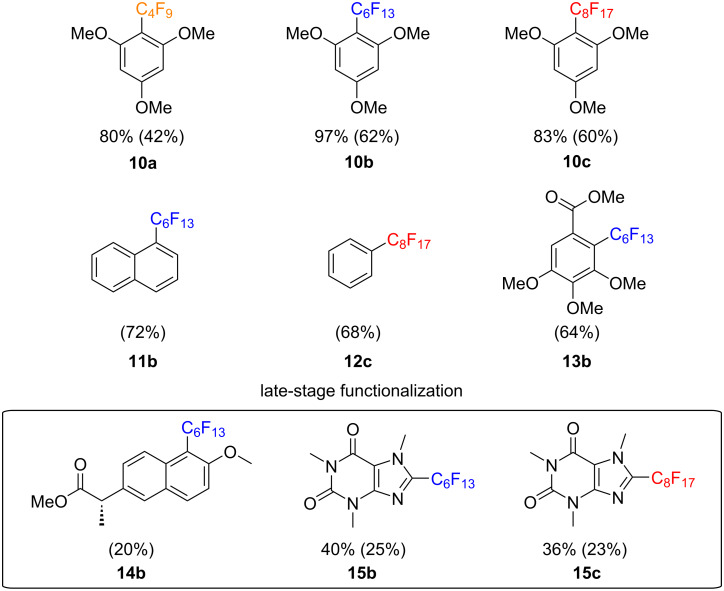
Demonstrative scope for the perfluoroalkylation of aromatics. Isolated yields are shown in parentheses.

Using 1,3,5-trimethoxybenzene (TMB, **9**) as a model substrate, we optimized the perfluoroalkylation reaction under irradiation of a 300 W Xenon arc lamp (Table 2). Based on the UV–vis absorption of our reagents **1**, we used long-pass filters at either 280 nm or 295 nm to avoid side reactions caused by shorter wavelengths. During this optimization, the use of 2 to 3 equivalents of the reagents **1** resulted in better yields, along with more concentrated reaction mixtures, reaching almost quantitative yields (by NMR) for the perfluorohexylation of TMB (**10b**) and 83% NMR yield for its perfluorooctylation (**10c**), both in less than 6 hours (Scheme 5). Unsubstituted arenes such as naphthalene were well tolerated in this methodology and produced 72% isolated yield of the perfluorohexylated product **11b**. The radical addition to unsubstituted benzene was also found to be possible affording perfluorooctylated product **12c** in 68% isolated yield, but as tends to be the case for inactivated substrates, excess quantities of benzene (50% v/v) were required. Compounds containing esters such as methyl 3,4,5-trimethoxybenzoate and naproxen methyl ester were also tolerated and the desired products **13b** and **14b** were isolated in yields of 64% and 20%, respectively. Arenes containing halogens were attempted; however, in accordance to previous reported literature, the compounds were found to decompose under the ultraviolet radiation necessary for the homolysis of the reagent [25]. Lastly, some heteroaromatic substrates such as *N*-phenylpyrrole and 2-phenylindole were found to produce large quantities of the desired perfluorohexyl and perfluorooctyl analogues as observed by both ^1^H NMR and GC–MS analysis. However, these molecules generated large concentrations of fluorinated byproducts which rendered separation of the products impossible. Furthermore, we tested this methodology on caffeine (Scheme 5), leading to a lower yield of products **15b** and **c**, due to its less electron-rich nature [26]. However, this yield was concordant with other radical innate functionalizations reported in the literature, showing the potential of these reagents as late-stage functionalization agents [26–27]. For a trend in reactivity, a more comprehensive scope of arenes and heteroarenes has been explored with the innate trifluoromethylation methodology previously reported by our group [10].

**Table 2:** Optimization for the perfluoroalkylation of aromatics under UV light.

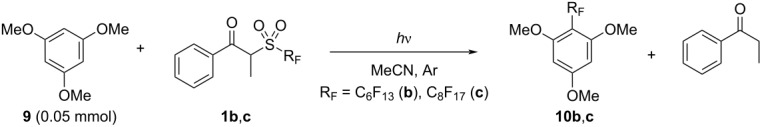					

Entry	Equiv reagent^a^	Vol. MeCN (mL)	Time (h)	Filter (nm)	NMR yield **10** (%)^b^

1	1	0.75	6	>295	20
2	2	0.75	6	>280	25
3	3	0.75	6	>295	35
4	1	0.75	**12**	>295	20
5	1	0.75	6	no filter	20
6	1	0.75	24	CFL^c^	traces
7	1	**0.75**	**18**	>295	20
8	1	**0.50**	6	>295	47
9	1	**0.25**	6	>280	47
10	2	**0.25**	**6**	>**280**	**97**
11	3	0.25	6	>**295**	**97**
12	2	**0.25**	**4**	>**295**	**90**
13	1	0.75	6	>295	36
14	2	**0.5**	**6**	>**295**	**83**

^a^Entries 1–12 were carried out with the perfluorohexyl analogue **1b**, entries 13 and 14 with the perfluorooctyl analogue **1c**; ^b^using dimethylsulfone as a standard; ^c^compact fluorescent lamp, 23 W.

## Conclusion

In summary, we have successfully developed a robust synthetic methodology for α-(perfluoroalkylsulfonyl)propiophenones, envisioned as new members of photocleavable perfluoroalkylating reagents. In this work, we have demonstrated their scalability and applicability in the metal-, catalyst- and additive-free, redox- and pH-neutral perfluoroalkylation of electron-rich aromatics, as well as in the late-stage functionalization of small molecules such as caffeine, which is of great interest in the current literature [28]. In future work, we will explore the reach and applicability of these reagents for the functionalization of compounds of interest in academia and industry, namely, for the synthesis of molecules with novel properties in the fields of material and bioorganic chemistry.

## Supporting Information

CCDC 2163755 contains the supplementary crystallographic data of byproduct B (perfluorooctyl analogue). These data can be obtained free of charge through the Cambridge Crystallographic Data Center (http://www.ccdc.cam.ac.uk/data_request/cif).

File 1 Detailed experimental procedures and compound characterization data.
