# Supplementary material for: Synthesis of α-(perfluoroalkylsulfonyl)propiophenones: a new set of reagents for the light-mediated perfluoroalkylation of aromatics
Source: Beilstein J Org Chem. 2022 Jul 4;18:788–95. doi: 10.3762/bjoc.18.79 (PMC9273985; doi:10.3762/bjoc.18.79)

BEILSTEIN JOURNAL OF ORGANIC CHEMISTRY

Supporting Information

for

Synthesis of α-(perfluoroalkylsulfonyl)propiophenones: a new set of reagents for the light-mediated perfluoroalkylation of aromatics

Durbis J. Castillo-Pazos, Juan D. Lasso and Chao-Jun Li

Beilstein J. Org. Chem. **2022,** *18,* 788–795. doi:10.3762/bjoc.18.79

Detailed experimental procedures and compound characterization data

License and Terms: This is a supporting information file under the terms of the Creative Commons Attribution License (https://creativecommons.org/licenses/by/4.0). Please note that the reuse, redistribution and reproduction in particular requires that the author(s) and source are credited and that individual graphics may be subject to special legal provisions.
The license is subject to the *Beilstein Journal of Organic Chemistry* terms and conditions: (https://www.beilstein-journals.org/bjoc/terms)

Table of contents

1. General experimental procedures: S1

2. General procedure for the synthesis of the perfluoroalkylating reagents **1a–c** (alkyl chains: -C_8F_{17}, -C_6F_{13}, -C_4F_9) S2

2.1 Step 1: Synthesis of sodium perfluoroalkyl sulfinates **2a–c** S2

2.2 Step 2: Synthesis of 2-iodopropiophenone (**6**) S2

2.3 Step 3: Synthesis of the 1-phenyl-2-perfluoroalkylsulfonyl-propan-1-ones **1a–c** S3

2.4 Step 4: Conversion of byproduct B S4

2.5 Single-crystal X-ray diffraction of byproduct B (perfluorooctyl analogue) S5

3. General procedure for the perfluoroalkylation of aromatics S6

3.1 Mechanistic evidence (Scheme 4) S7

4. Characterization data of reported compounds: S8

5. References S12

Spectra collection S13

1. General experimental procedures:

All reactions were carried out under argon atmosphere using standard Schlenk technique. ^{1}H NMR (500 MHz), ^{13}C NMR (126 MHz) and ^{19}F NMR (471 MHz) were recorded on an NMR spectrometer with $CDCl_3$ or acetone-d_6 as the solvent. Chemical shifts of ^{1}H, ^{13}C and ^{19}F NMR spectra are reported in parts per million (ppm). The residual solvent signals were used as references and the chemical shifts were converted to the TMS scale ($CDCl_3$: δ H = 7.26 ppm, δ C = 77.16 ppm). All coupling constants (*J* values) are reported in Hertz (Hz). High-resolution mass spectrometry was conducted through using atmospheric pressure chemical ionization (APCI) or electro-spraying ionization (ESI), and was performed at McGill University on a Thermo-Scientific Exactive Orbitrap. Protonated molecular ions $[M + H]^+$ or sodium adducts $[M+Na]^+$ were used for empirical formula confirmation. Column chromatography was performed either on silica gel 200−300 mesh, or on C8-reversed-phase perfluorinated silica gel.

2. **General procedure for the synthesis of the perfluoroalkylating reagents 1a–c (alkyl chains: $-C_8F_{17}$, $-C_6F_{13}$, $-C_4F_9$)**

2.1 Step 1: Synthesis of sodium perfluoroalkyl sulfinates 2a–c

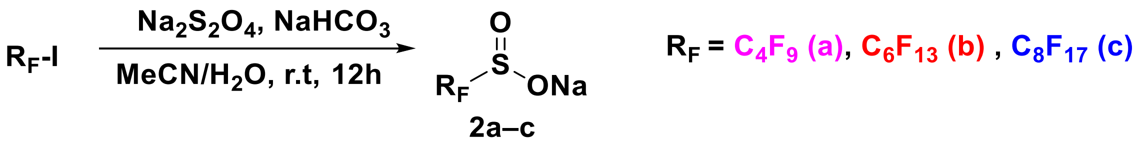

The reaction was performed in an open 100 mL flask with a magnetic stirring bar. Sodium dithionite (5.22 g, 30 mmol) and sodium bicarbonate (2.0 g, 24 mmol) were suspended in a water/acetonitrile 2:1 solution (45 mL) cooled with a bath of ice and salt. Then, 25 mmol of the corresponding perfluoroalkyl iodide (perfluorooctyl-, perfluorohexyl-, perfluorobutyl-) were added. The reaction was left overnight to reach ambient temperature.[1] During this time, the generation of gas (carbon and sulfur dioxides) is observed, and the solution turns from colorless to pale transparent yellow with no presence of suspended salts.

Once the reaction finished, the crude was evaporated to dryness while stirring at 82–100 °C. The solids at the bottom of the flask were washed with acetone until the fractions collected were colorless and passed through a fritted glass filter. For perfluorohexyl (**2b**) and perfluorooctyl sulfinate (**2c**) salts, the collected acetone fractions were evaporated with a rotary evaporator and under high vacuum to yield a pale-yellow solid that was later recrystallized from acetonitrile to give the final sodium sulfinate salt as a white crystalline salt. The crude sodium perfluorobutyl sulfinate (**2a**), was too soluble in acetonitrile and was instead washed with DCM until colorless on a fritted glass filter and allowed to dry. Isolated yields might range from quantitative to 50%, depending on the correct performance of the recrystallization step.

2.2 Step 2: Synthesis of 2-iodopropiophenone (6)

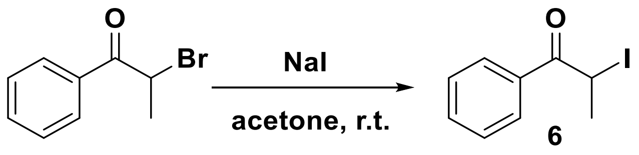

A solution of 20 mmol of sodium iodide (3.0 g) in 50 mL of acetone was stirred in a 100 mL open flask at room temperature for one or two minutes until saturation of the salt was evidenced by the appearance of a pale-yellow color. 2-Bromopropiophenone (2.13 g, 10 mmol) was added dropwise while stirring vigorously, leading to the formation of a cloudy yellow suspension due to the precipitation of sodium bromide (Finkelstein reaction) [2]. After 15 minutes, acetone was evaporated on a rotary evaporator and the product was extracted 3 times with ethyl acetate, washed with brine, and dried over anhydrous sodium sulfate. If the solution turned to a darker tone of brown, washing with saturated sodium thiosulfate would suffice to eliminate the forming iodine. The dried organic phases were evaporated to give a yellow oil in quantitative yield. The resulting product is sensitive to light and was typically synthesized for use on same day.

2.3 Step 3: Synthesis of the 1-phenyl-2-perfluoroalkylsulfonyl-propan-1-ones 1a–c

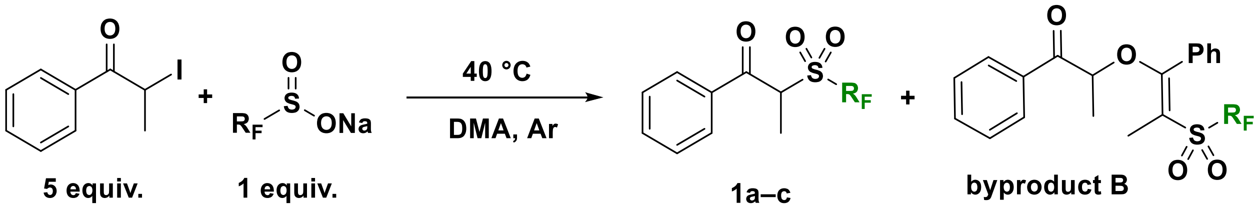

R_F = $-C_8F_{17}$, $-C_6F_{13}$, $-C_4F_9$

In a 25 mL flask equipped with a stirring bar, 3 mmol of the corresponding sodium sulfinate and 5 equivalents of 2-iodopropiophenone (13 g) were dissolved in 5 mL of dried DMA. The reaction was stirred vigorously for 16 h at 40 °C under inert atmosphere. Over time, the reaction mixture turned black due to the generation of iodine in solution. Once it was finished, the reaction was quenched with 30 mL of water, and the product was extracted with ethyl acetate (3 × 10 mL). The organic fractions were collected, washed with saturated sodium thiosulfate and brine, and evaporated. The excess of 2-iodopropiophenone was separated from the desired product through a C8 perfluorinated silica plug (40 g) using the following gradient: methanol/water 2:1 → methanol → acetone. The methanol and acetone fractions were collected and evaporated to give a clean mixture products **1a**–**c** and byproduct B (2:1, respectively) as a white powder. Isolated yield of the product at this stage can be up to 50%. The reaction demonstrated a scalability of up to 6 grams of product. Recycling of excess 2-iodopropiophenone utilized can be achieved by silica column with a gradient of 0% to 10% ethyl acetate in hexanes.

Note: When running the crude through the perfluorinated silica plug, constant pressure must be maintained for optimal separation. A successful separation is shown below:

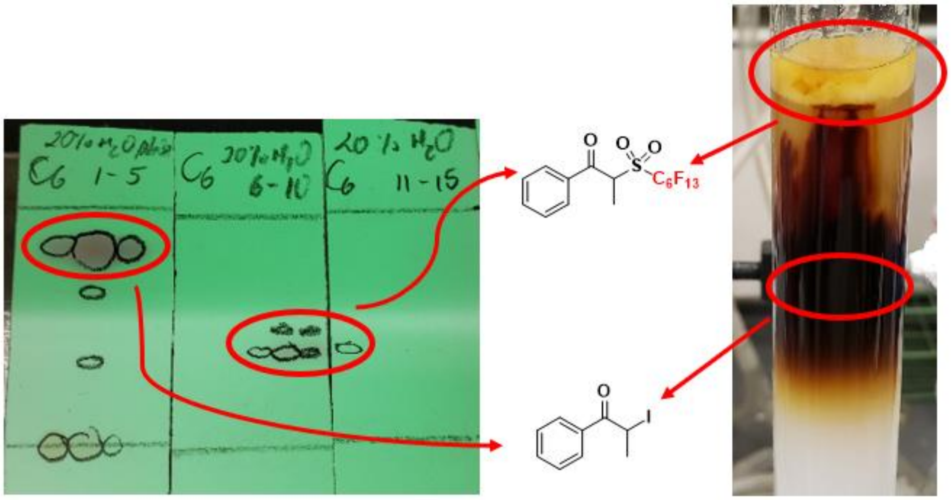

2.4 Step 4: Conversion of byproduct B

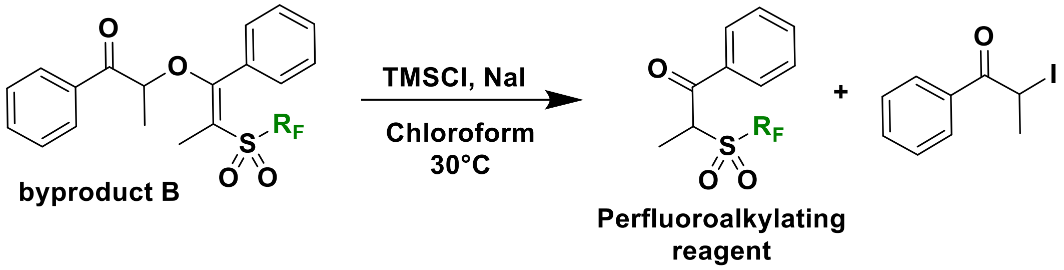

In a 50 mL flask, dissolve the mixture obtained in step 3 in the minimum amount possible of chloroform (to form a solution around 0.4 M). While stirring vigorously, add 5 equivalents of chlorotrimethylsilane and 5 equivalents of sodium iodide (calculated against the amount of byproduct B detected by NMR). Monitor the reaction at 30 °C until completion (approximately 1–3 hours) and wash with one portion of each: saturated bicarbonate solution, saturated sodium thiosulfate solution, and brine. After evaporating the chloroform on a rotary evaporator, the crude is purified through a C8 perfluorinated silica plug (40 g) using the following gradient: methanol/water 2:1 → methanol → acetone. The methanol and acetone fractions are collected and evaporated to give the final perfluoroalkylating reagent as a white powder. Isolated yield of the product at this final stage can be up to 70%.

The most efficient way of monitoring the amount of byproduct present in the crude (if any) is to trace the proton in the alpha position through NMR as shown in the right:

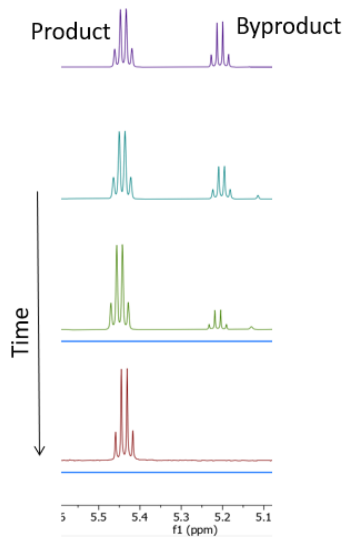

2.5 Single-crystal X-ray diffraction of byproduct B (perfluorooctyl analogue)

The byproduct was separated via column chromatography (100% toluene) and recrystallized from methanol over the course of 3 days at 4 °C to yield colorless needles. A Bruker D8 Advance powder X-ray diffractometer was used for the structure refinement and collection of the following crystal data:

Empirical formula: $C_{26}H_{17}F_{17}O_4S$<br>Formula weight: 748.46<br>Temperature/K: 180(2)<br>Crystal system: monoclinic<br>Space group: C2/c<br>a/Å: 47.7488(17)<br>b/Å: 8.7035(3)<br>c/Å: 14.0715(5)<br>α/°: 90, β/°: 93.0800(10), γ/°: 90<br>Volume/Å^3: 5839.4(4)<br>Z: 8<br>ρcalcg/cm^3: 1.703<br>μ/mm^{-1}: 2.329<br>F(000): 2992.0	Crystal size/mm3 0.540 × 0.320 × 0.310<br>Radiation CuKα (λ = 1.54178)<br>2Θ range for data collection/° 7.416 to 144.608<br>Index ranges -58 ≤ h ≤ 58, -10 ≤ k ≤ 10, -12 ≤ l ≤ 17<br>Reflections collected: 53403<br>Independent reflections: 5678 [Rint = 0.0485, Rsigma = 0.0260]<br>Data/restraints/parameters: 5678/0/435<br>Goodness-of-fit on F2: 1.042<br>Final R indexes [I>=2σ (I)] R1 = 0.0406, wR2 = 0.1067<br>Final R indexes [all data] R1 = 0.0427, wR2 = 0.1090<br>Largest diff. peak/hole / e Å-3 0.28/-0.37

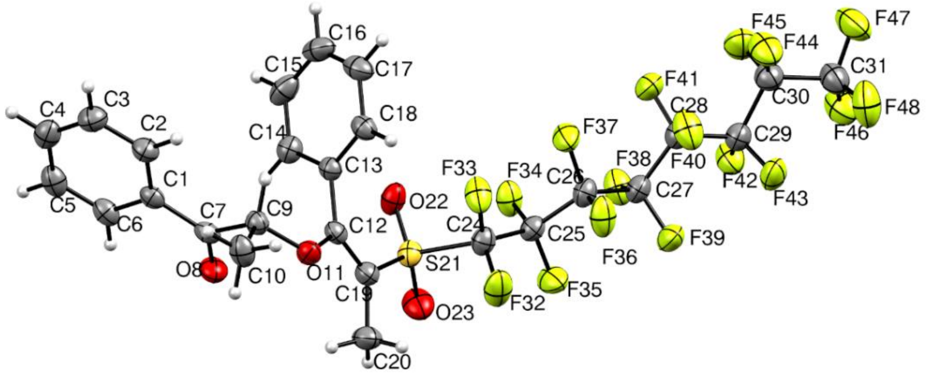

3. General procedure for the perfluoroalkylation of aromatics

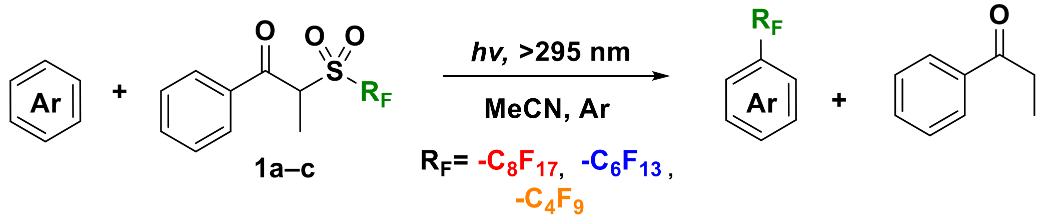

The aromatic substrate (0.1 mmol) and corresponding perfluoroalkylating reagent **1** (0.15–0.2 mmol) were added into 0.5 mL acetonitrile inside an air-tight quartz. Three freeze-pump-thaw cycles were carefully performed before setting the reaction under light irradiation at 20 °C using a 300 W xenon lamp. Temperature was controlled by a cold-water bath in a jacketed glass container connected to a cooler. After the reaction was finished (4–12 h), the product diluted with dichloromethane or acetone, evaporated under reduced pressure, and purified through a C8 perfluorinated silica plug (10–20 g) using the following gradient: methanol/water 2:1 → methanol → acetone. Methanol and acetone fractions were collected and evaporated to give the final product. An alternative purification method consists of preparative thin-layer chromatography, using ethyl acetate/hexanes or dichloromethane/hexanes as the eluent.

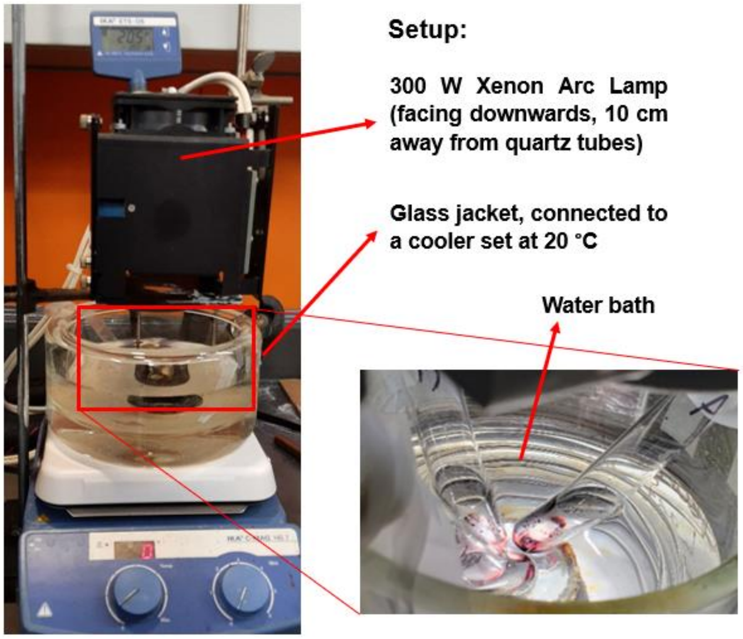

3.1 Mechanistic evidence (Scheme 4)

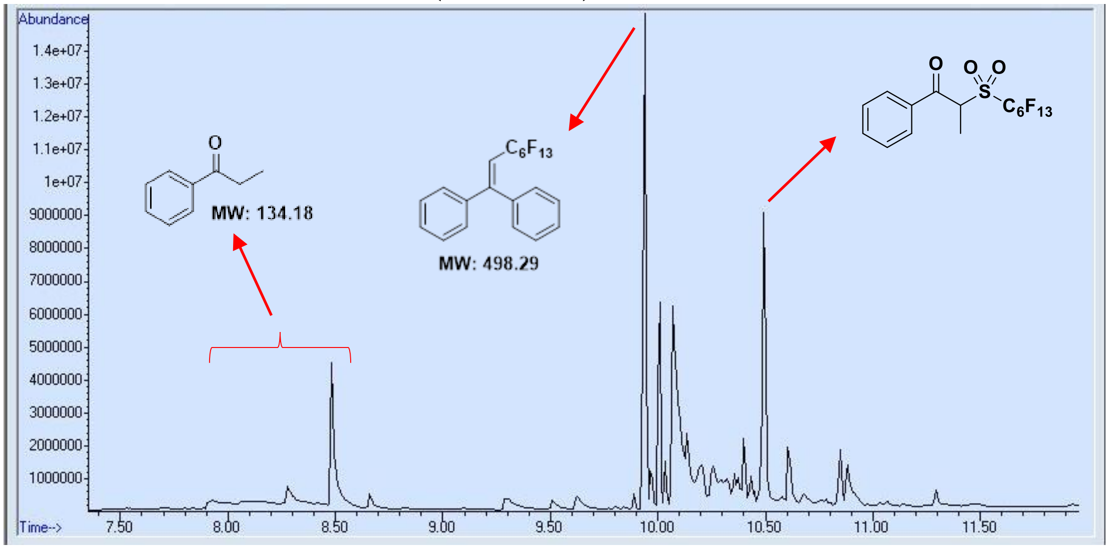

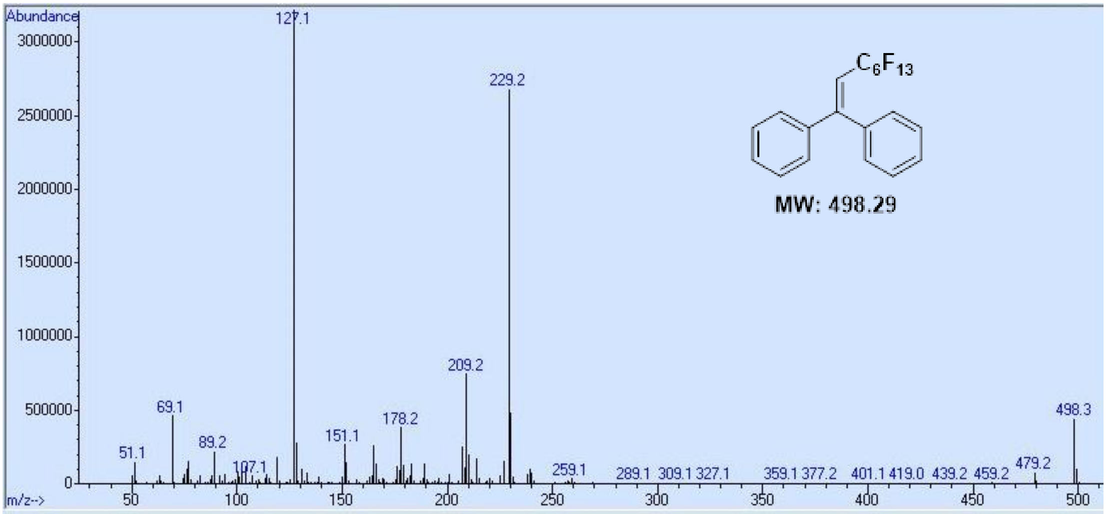

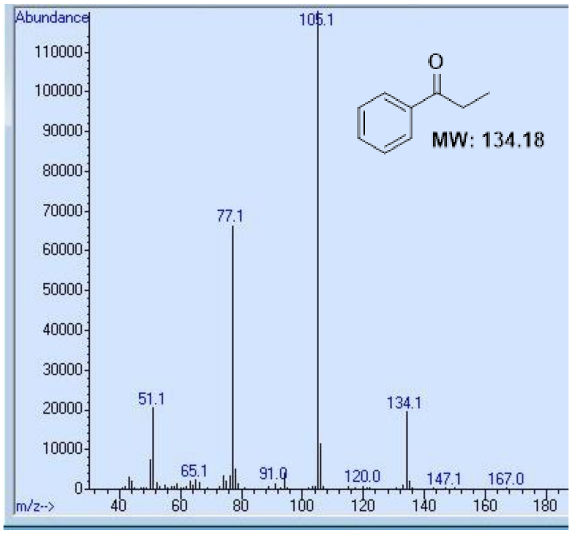

Detection of sulfur dioxide: A paper strip was coated in an acidic solution of potassium dichromate and was used to cap a needle that punctured the sealed reaction vessel. Over the course of 20 min, a blue-green coloration developed in the exposed area, indicating the presence of SO_2

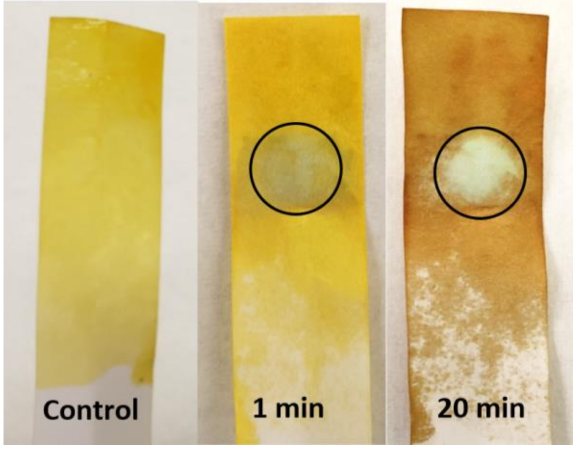

$$K_2Cr_2O_7 + H_2SO_4 + 3SO_2 \longrightarrow K_2SO_4 + Cr_2(SO_4)_3 + H_2O$$

4. Characterization data of reported compounds:

2a 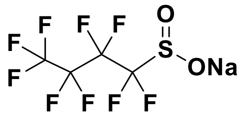

Sodium perfluorobutylsulfinate (2a) [3]: White crystals. Yield: 54%. ^{19}F NMR (471 MHz, Acetone) δ -81.90 – -82.00 (m), -124.32 (dd, J = 14.6, 7.3 Hz), -127.01 – -127.12 (m), -132.58 (td, J = 10.5, 9.1, 6.5 Hz). HRMS: m/z= 282.94816, theoretical= 282.94808

2b 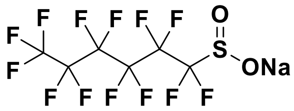

Sodium perfluorohexylsulfinate (2b) [3]: White crystals. Quantitative yield (highly dependent on workup conditions). ^{19}F NMR (471 MHz, Acetone) δ -81.58 – -81.85 (m), -115.16 – -115.45 (m), -121.11, -122.36, -123.36, -126.78 (ddd, J = 18.8, 11.1, 5.5 Hz). HRMS: m/z= 382.94052, theoretical= 382.94169

2c 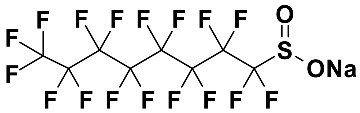

Sodium perfluorooctylsulfinate (2c) [4]: White crystals. Quantitative yield (highly dependent on workup conditions). ^{19}F NMR (471 MHz, Acetone) δ -81.67 (t, J = 10.1 Hz), -122.55 (d, J = 12.7 Hz), -123.26 (d, J = 19.5 Hz), -126.74, -132.39. HRMS: m/z= 482.93492, theoretical= 382.93530

1c

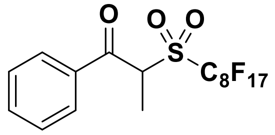

2-((Perfluorooctyl)sulfonyl)-1-phenylpropan-1-one (1c): White powder. 75% yield. ^{1}H NMR (500 MHz, $CDCl_3$) δ 8.00 – 7.94 (m, 2H), 7.72 – 7.65 (m, 1H), 7.58 – 7.51 (m, 2H), 5.42 (q, *J* = 7.0 Hz, 1H), 1.84 (dd, *J* = 7.0, 1.0 Hz, 3H). ^{13}C NMR (126 MHz, Acetone) δ 155.20, 150.96, 146.94, 136.54, 134.05, 128.72, 110.37, 33.40, 27.25. ^{19}F NMR (471 MHz, CDCl3) δ -80.76, -109.18, -119.94, -121.36, -121.69, -121.90, -122.72, -126.11.

1b

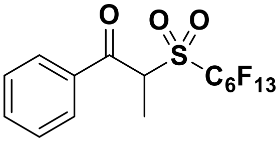

2-((Perfluorohexyl)sulfonyl)-1-phenylpropan-1-one (1b): White powder. 66% yield. ^{1}H NMR (500 MHz, CDCl3) δ 8.02 – 7.96 (m, 2H), 7.75 – 7.68 (m, 1H), 7.61 – 7.54 (m, 2H), 5.44 (q, J = 7.0 Hz, 1H), 1.87 (d, J = 7.0 Hz, 3H). 1^{3}C NMR (126 MHz, CDCl3) δ 188.83, 135.07, 134.90, 129.21, 128.96, 61.86, 13.17. ^{19}F NMR (471 MHz, CDCl3) δ -80.72 (t, J = 10.0 Hz), -109.19 (t, J = 14.9 Hz), -119.97 (ddt, J = 30.5, 18.6, 7.6 Hz), -121.59 (d, J = 22.6 Hz), -122.50 – -122.72 (m), -126.05 (ddq, J = 14.3, 10.9, 4.0 Hz).

1a

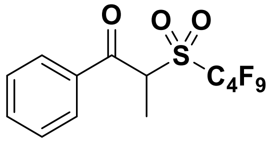

2-((Perfluorobutyl)sulfonyl)-1-phenylpropan-1-one (1a): Yellow solid (melts at room temperature). 30% yield. ^{1}H NMR (500 MHz, Acetone) δ 8.20 – 8.14 (m, 4H), 7.82 – 7.74 (m, 2H), 7.68 – 7.61 (m, 4H), 6.19 – 6.11 (m, 1H), 1.90 – 1.84 (m, 3H), 1.86 (s, 3H), 1.31 (s, 2H), 0.90 (t, *J* = 6.3 Hz, 1H). ^{19}F NMR (471 MHz, Acetone) δ -81.56 – -81.68 (m), -110.44 – -110.62 (m), -121.70 – -121.91 (m), -126.52 (td, *J* = 14.6, 14.1, 7.0 Hz).

10c

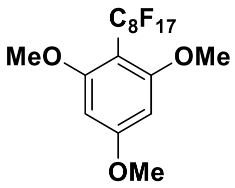

2-(Perfluorooctyl)-1,3,5-trimethoxybenzene (10c) [5]: White solid. 60% yield. ^{1}H NMR (500 MHz, CDCl3) δ 6.17 (s, 2H), 3.86 (s, 3H), 3.83 (s, 6H). ^{13}C NMR (126 MHz, CDCl3) δ 163.86, 161.76, 161.74, 91.70, 56.26, 55.28. ^{19}F NMR (471 MHz, CDCl3) δ -80.88 (t, J = 10.0 Hz), -102.51 – -102.80 (m), -121.61 – -121.91 (m), -121.91 – -122.21 (m), -122.75 (dq, J = 26.7, 13.2, 11.2 Hz), -126.18 (dq, J = 14.5, 6.6 Hz).

10b

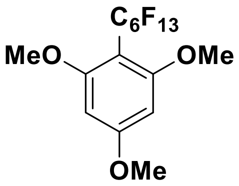

2-(Perfluorohexyl)-1,3,5-trimethoxybenzene (10b) [5]: White solid. 62% yield. ^{1}H NMR (500 MHz, CDCl3) δ 6.17 (s, 2H), 3.86 (s, 3H), 3.83 (s, 6H). ^{13}C NMR (126 MHz, CDCl3) δ 163.85, 161.76, 91.71, 56.30, 55.32. ^{19}F NMR (471 MHz, CDCl3) δ -80.78 – -80.98 (m), -102.56 – -102.72 (m), -122.03 – -122.33 (m), -122.69 (qd, J = 16.8, 13.2, 5.7 Hz), -126.17 (td, J = 14.3, 6.4 Hz).

10a

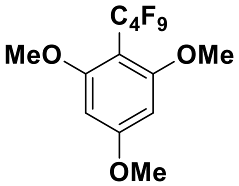

2-(Perfluorobutyl)-1,3,5-trimethoxybenzene (10a) [5]: White solid. 42% yield. ^{1}H NMR (500 MHz, CDCl3) δ 6.17 (d, J = 1.0 Hz, 2H), 3.86 (s, 3H), 3.83 (s, 6H). ^{13}C NMR (126 MHz, CDCl3) δ 163.84, 161.75, 91.71, 56.32, 55.33. ^{19}F NMR (471 MHz, CDCl3) δ -80.89 – -81.05 (m), -102.83 (td, J = 13.6, 3.0 Hz), -122.97 (pq, J = 10.2, 4.6 Hz), -126.33 – -126.48 (m).

11b

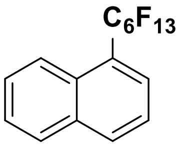

1-Perfluorohexylnaphthalene (11b): White solid. 72% yield. ^{1}H NMR (500 MHz, $CDCl_3$) δ 8.26 (d, *J* = 8.7 Hz, 1H), 8.09 (d, *J* = 8.2 Hz, 1H), 7.95 (dt, *J* = 9.1, 2.7 Hz, 1H), 7.86 (dd, *J* = 7.4, 1.2 Hz, 1H), 7.67 – 7.56 (m, 3H). ^{13}C NMR (201 MHz, CDCl3) δ 134.10, 133.43, 130.29, 129.00, 128.03, 127.65, 127.16, 126.38, 124.83, 124.25. ^{19}F NMR (471 MHz, $CDCl_3$) δ -80.77, -104.42, -120.26, -121.46, -122.73, -126.07.

12c

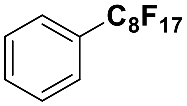

Perfluorooctylbenzene (12c): Yellow oil. 68% yield.^{1}H NMR (500 MHz, $CDCl_3$) δ 7.64 – 7.57 (m, 3H), 7.53 (t, *J* = 7.6 Hz, 2H). ^{13}C NMR (201 MHz, $CDCl_3$) δ 131.92, 128.60, 126.78. ^{19}F NMR (471 MHz, $CDCl_3$) δ -80.81, -110.73, -121.27, -121.90, -122.73, -126.19.

13b

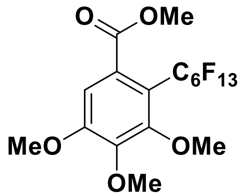

Methyl 3,4,5-trimethoxy-2-(perfluorohexyl)benzoate (13b) [6]: White solid. 64% yield. ^{1}H NMR (500 MHz, $CDCl_3$) δ 6.78 (s, 1H), 3.94 (d, *J* = 1.5 Hz, 6H), 3.91 (s, 3H), 3.89 (s, 3H). ^{13}C NMR (201 MHz, $CDCl_3$) δ 168.61, 156.17, 153.90, 144.01, 136.89, 136.16, 133.34, 132.93, 130.17, 128.79, 128.61, 128.50, 128.48, 107.33, 61.91, 60.85, 56.26, 52.86. ^{19}F NMR (471 MHz, $CDCl_3$) δ -80.74, -100.99.

14b

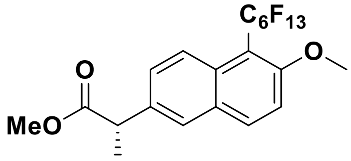

Naproxen methyl ester-C_6F_{13} 14b: White solid. 20% yield. ^{1}H NMR (800 MHz, $CDCl_3$) δ 8.15 (d, *J* = 9.2 Hz, 1H), 7.98 (d, *J* = 9.1 Hz, 1H), 7.70 (d, *J* = 2.1 Hz, 1H), 7.49 (dd, *J* = 9.2, 2.1 Hz, 2H), 7.34 (d, *J* = 9.1 Hz, 1H), 3.97 (s, 3H), 3.87 (q, *J* = 7.3 Hz, 1H), 3.68 (s, 3H), 1.58 (d, *J* = 7.1 Hz, 5H). ^{13}C NMR (201 MHz, $CDCl_3$) δ 174.76, 159.04, 136.18, 134.93, 131.38, 129.46, 126.87, 125.18, 114.68, 57.42, 52.16, 44.93, 18.38. ^{19}F NMR (471 MHz, $CDCl_3$) δ -80.72, -100.17, -120.67, -121.94, -122.61, -126.06.

15b

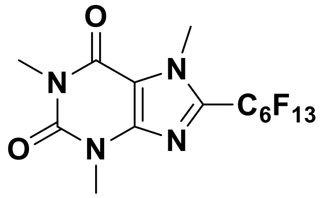

Caffeine-C_6F_{13} 15b [7]: White solid. 25% yield. ^{1}H NMR (500 MHz, Acetone) δ 4.26 (t, J = 2.0 Hz, 6H), 3.51 (s, 6H), 3.33 (s, 6H), 2.10 (d, J = 1.2 Hz, 1H), 1.31 (s, 1H). ^{13}C NMR (126 MHz, Acetone) δ 155.20, 150.95, 146.94, 129.52, 128.43, 110.37, 33.40, 27.25. ^{19}F NMR (471 MHz, Acetone) δ -81.51 – -81.84 (m), -81.71, -109.18 (t, J = 14.4 Hz), -121.34 – -121.70 (m), -121.84, -122.32, -123.28, -126.69 (ddq, J = 19.5, 11.7, 4.0 Hz).

15c

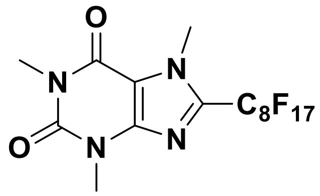

Caffeine-C_8F_{17} 15c [7]: White solid. 23% yield. ^{1}H NMR (500 MHz, Acetone) δ 4.26 (t, J = 2.0 Hz, 3H), 3.52 (s, 3H), 3.33 (s, 3H). ^{19}F NMR (471 MHz, Acetone) δ -81.64 (t, J = 10.3 Hz), -109.16 (t, J = 14.3 Hz), -121.27 – -121.80 (m), -122.22, -122.33, -123.21, -126.51 – -126.89 (m).

byproduct B

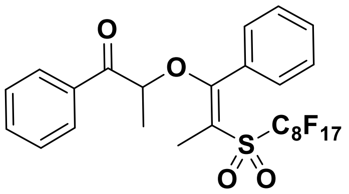

(*E*)-2-((2-((Perfluorooctyl)sulfonyl)-1-phenylprop-1-en-1-yl)oxy)-1-phenylpropan-1-one (byproduct B): White solid.^{1}H NMR (500 MHz, acetone) δ 7.58 – 7.54 (m, 2H), 7.45 – 7.41 (m, 2H), 7.37 (tt, *J* = 7.5, 1.3 Hz, 1H), 5.50 (q, *J* = 6.9 Hz, 1H), 2.33 (d, *J* = 0.8 Hz, 3H), 1.55 (d, *J* = 6.9 Hz, 3H). ^{13}C NMR (126 MHz, acetone) δ 205.15, 197.07, 189.54, 173.10, 142.56 – 120.65 (m), 133.71, 130.22, 129.14, 129.08, 128.74, 127.86, 109.73, 77.13, 62.37, 17.97, 12.78, 12.77. ^{19}F NMR (471 MHz, Acetone) δ -81.58 – -81.69 (m), -111.91 (dd, J = 17.3, 13.0 Hz), -120.88 (tt, J = 14.0, 5.5 Hz), -121.47 – -122.86 (m), -123.22, -126.02 – -128.00 (m).

5. References

1. Dmowski, W.; Piasecka-Maciejewska, K. *J. Fluorine Chem.* **2005**, *126*, 877-882.
2. Finkelstein, H. *Berichte der deutschen chemischen Gesellschaft* **1910**, *43*, 1528-1532.
3. Hu, C.; Qing, F.; Huang, W. *J. Org. Chem.* **1991**, *56*, 2801-2804.
4. Clavel, J.-L.; Langlois, B.; Nantermet, R.; Tordeux, M.; Wakselman, C. *J. Chem. Soc., Perkin Trans. 1* **1992**, 3371-3375, 10.1039/P19920003371.
5. Tasnim, T.; Ryan, C.; Christensen, M. L.; Fennell, C. J.; Pitre, S. P. *Org. Lett.* **2022**, *24*, 446-450.
6. JP Patent: JPS5775932A
7. Wei, Z.; Qi, S.; Xu, Y.; Liu, H.; Wu, J.; Li, H.; Xia, C.; Duan, G. *Adv. Synth. Catal.* **2019**, *361*, 5490-5498, https://doi.org/10.1002/adsc.201900885.

Spectra collection

^{19}F NMR of **2a**, acetone-d_6, 471 MHz

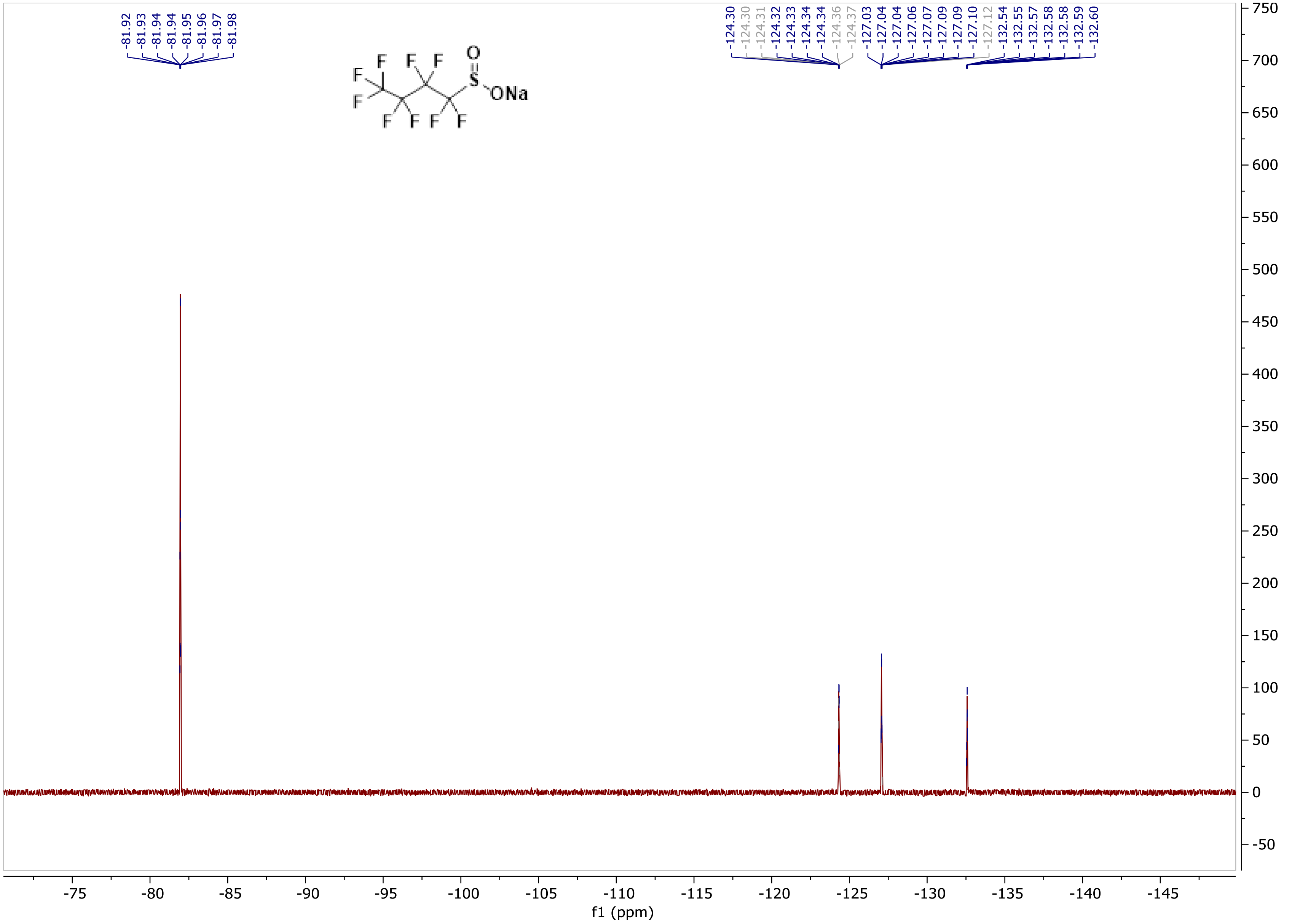

^{19}F NMR of **2b**, acetone-d_6, 471 MHz

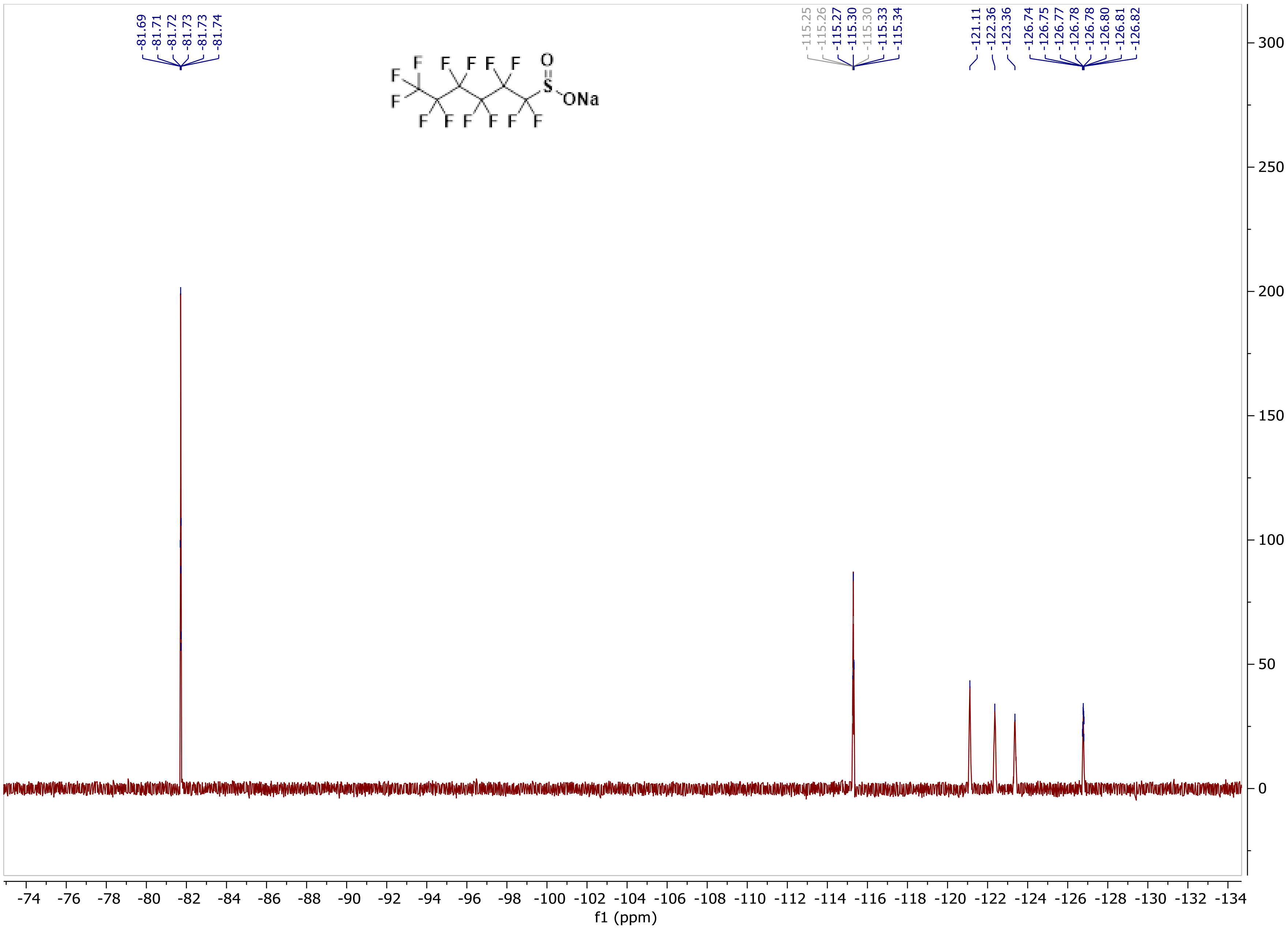

^{19}F NMR of **2c**, acetone-d^6, 471 MHz

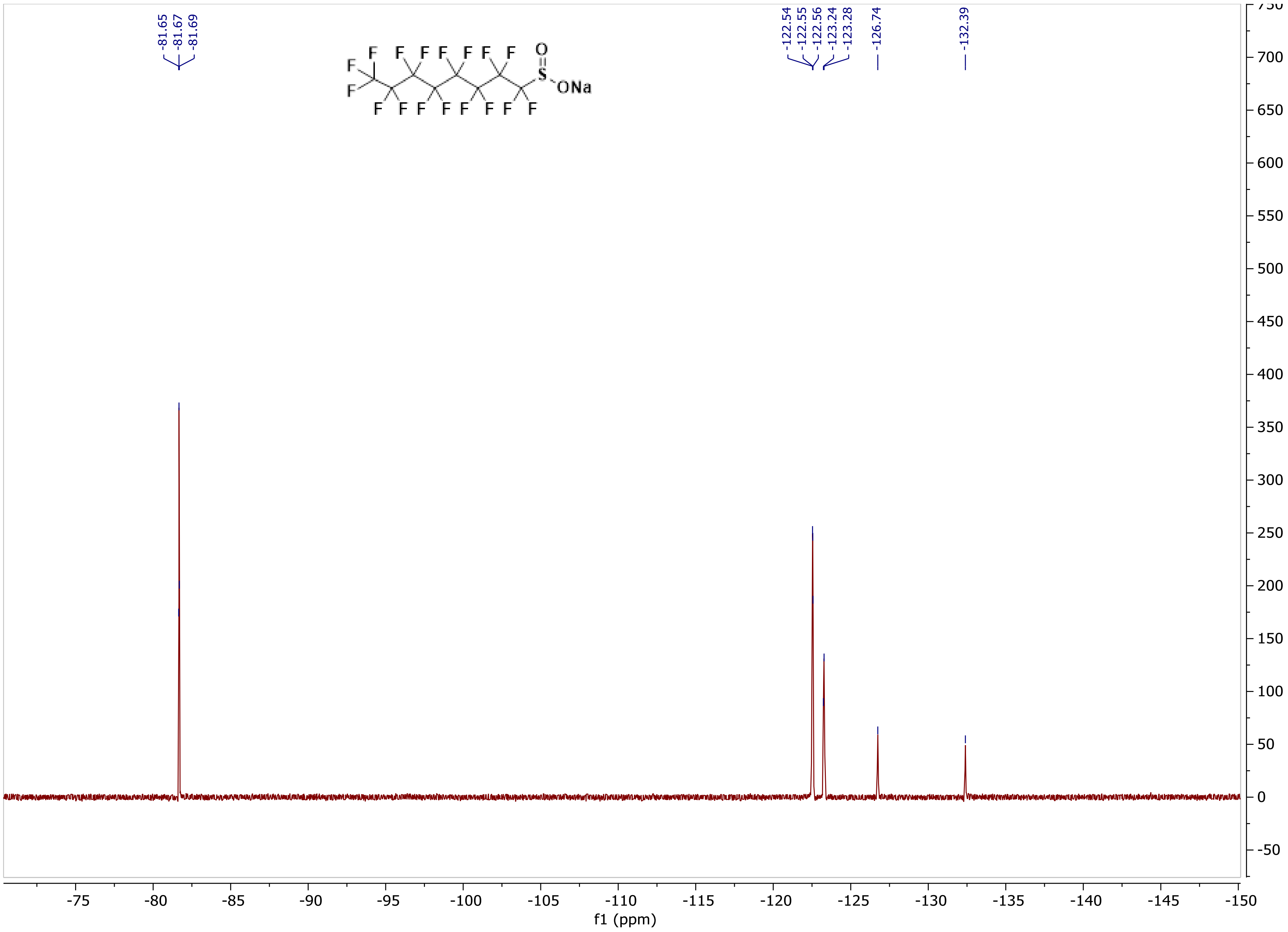

^{1}H NMR of **1c**, CDCl$_3$, 500 MHz

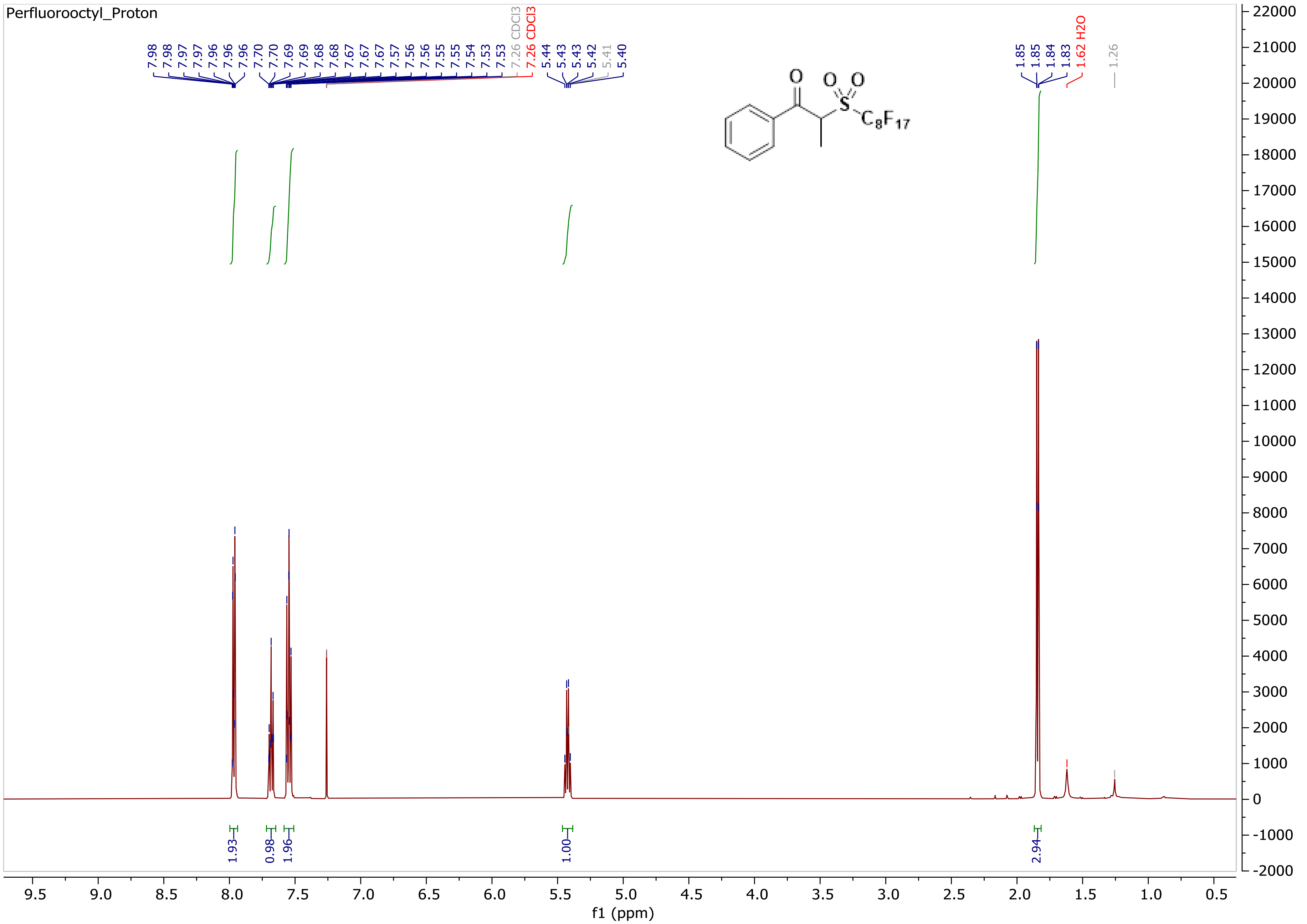

^{13}C NMR of **1c**, $CDCl_3$, 126 MHz

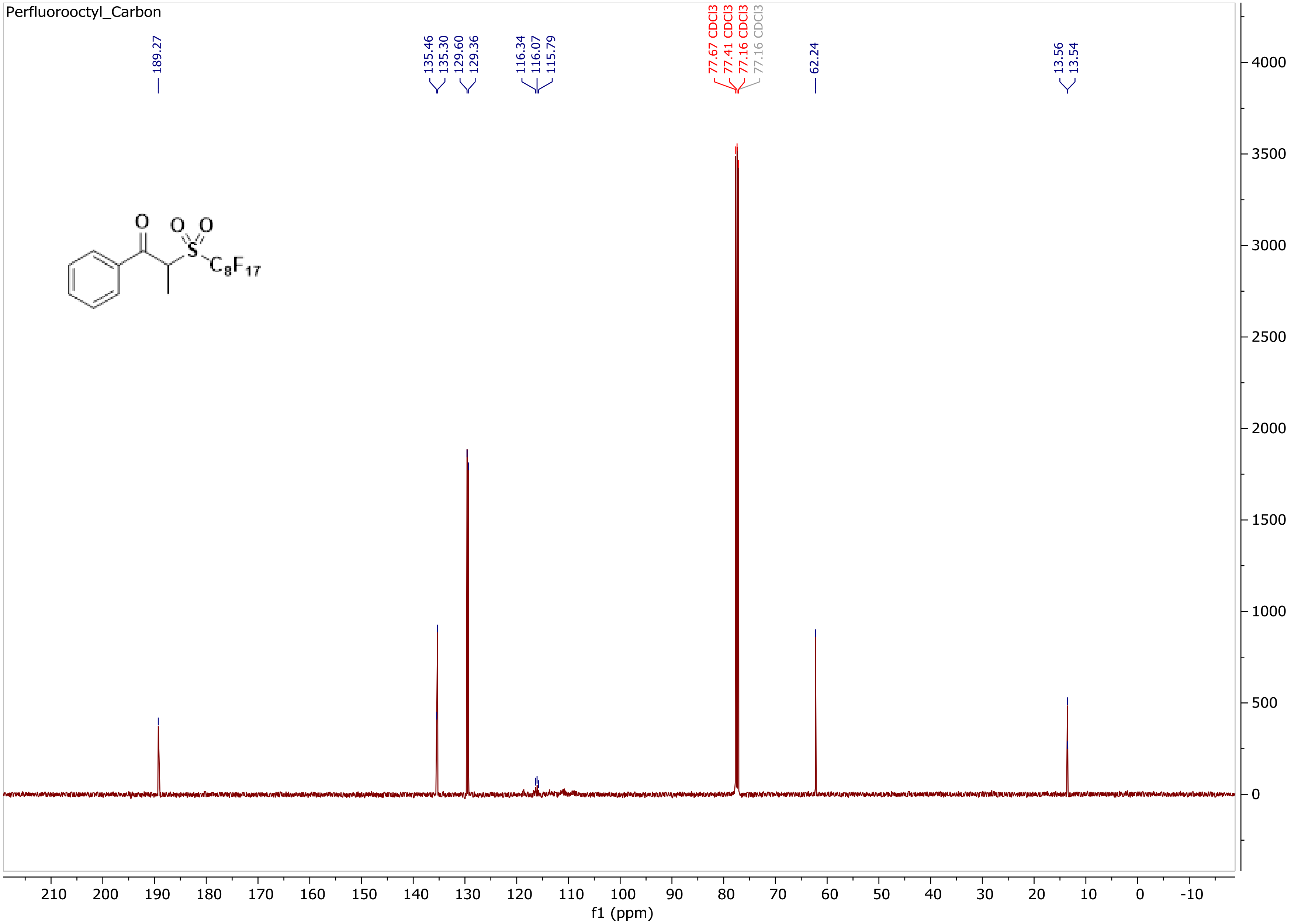

^{19}F NMR of **1c**, $CDCl_3$, 471 MHz

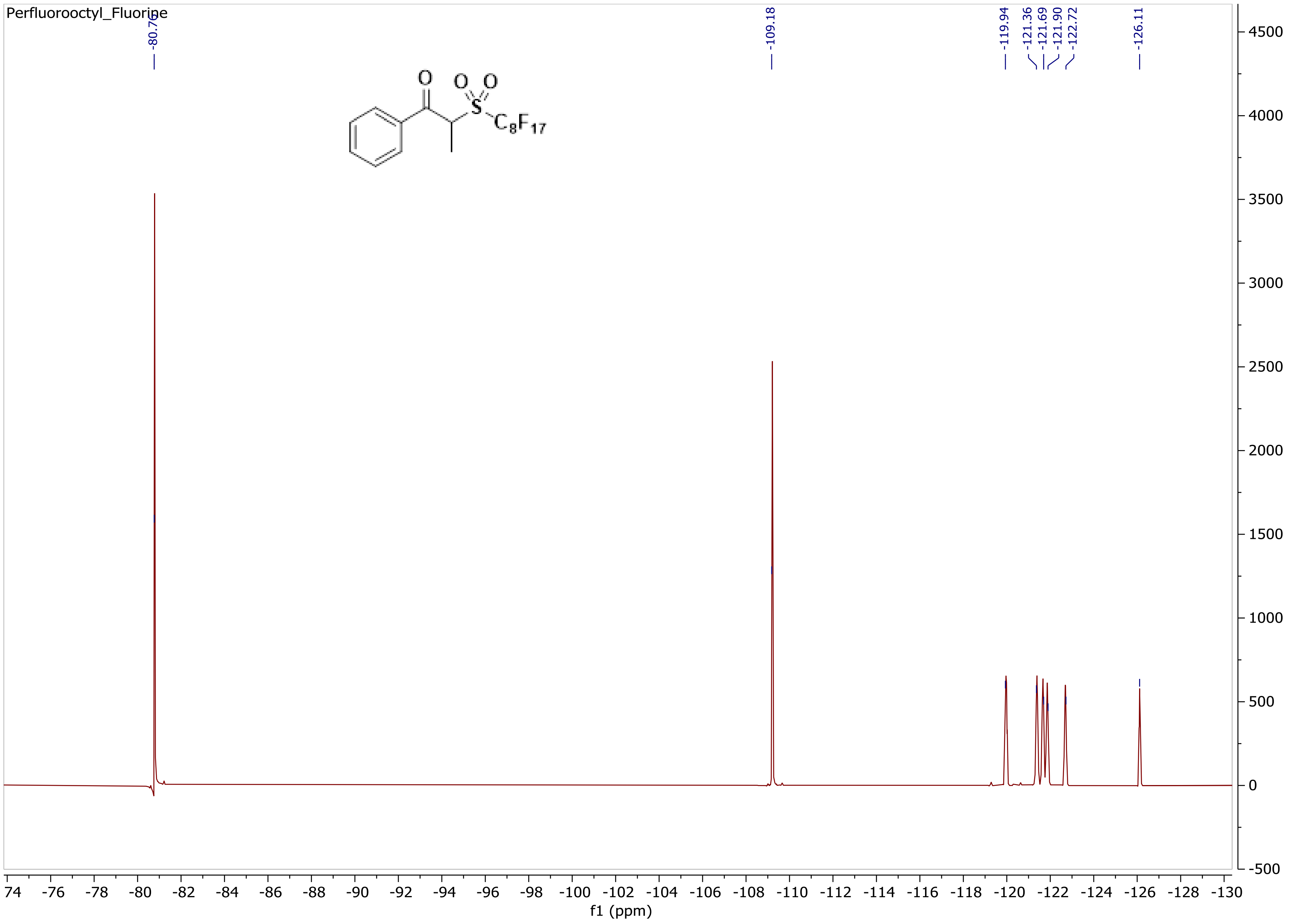

^{1}H-^{19}F HMBC of **1c**

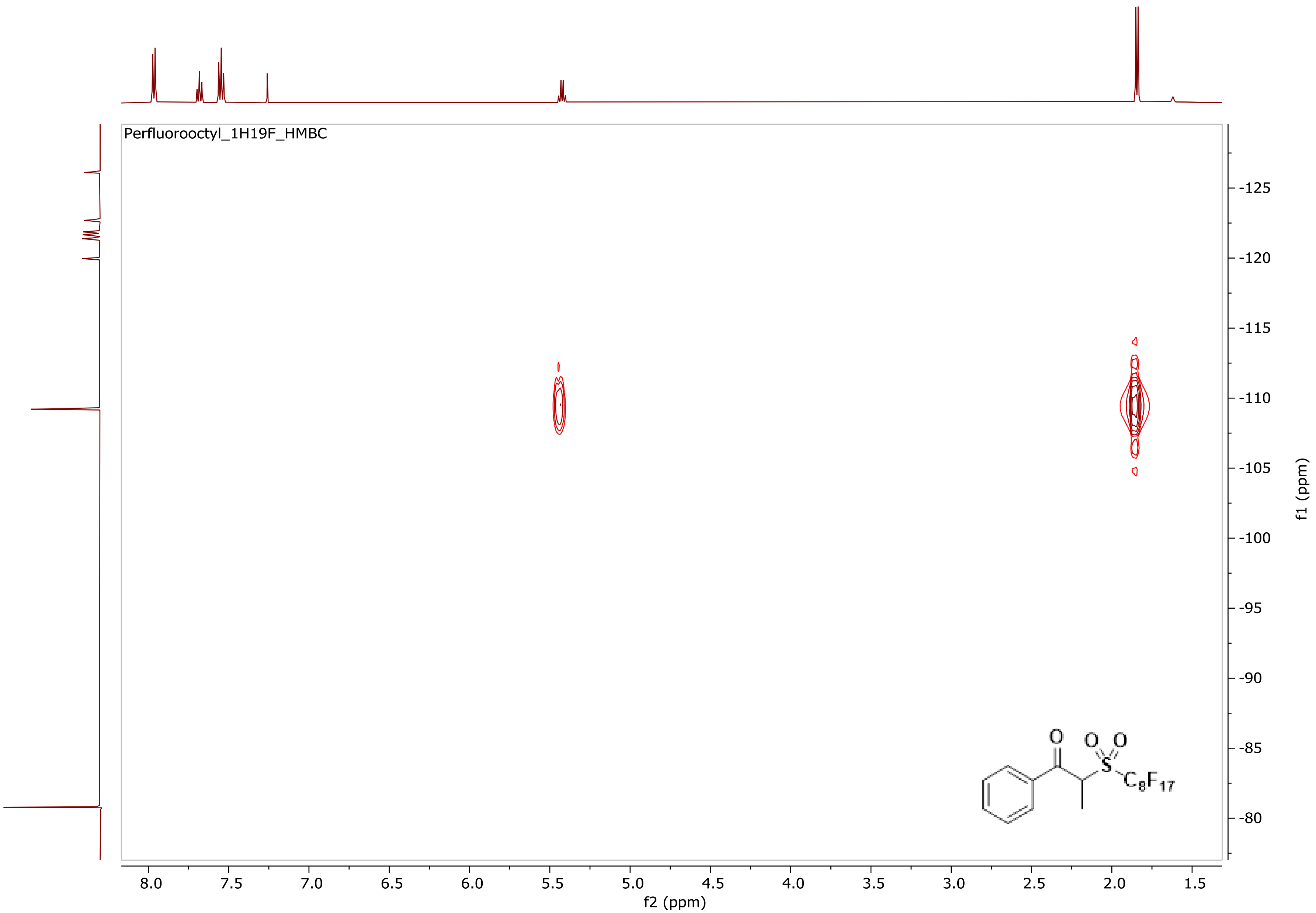

^{1}H NMR of **1b**, $CDCl_3$, 500 MHz

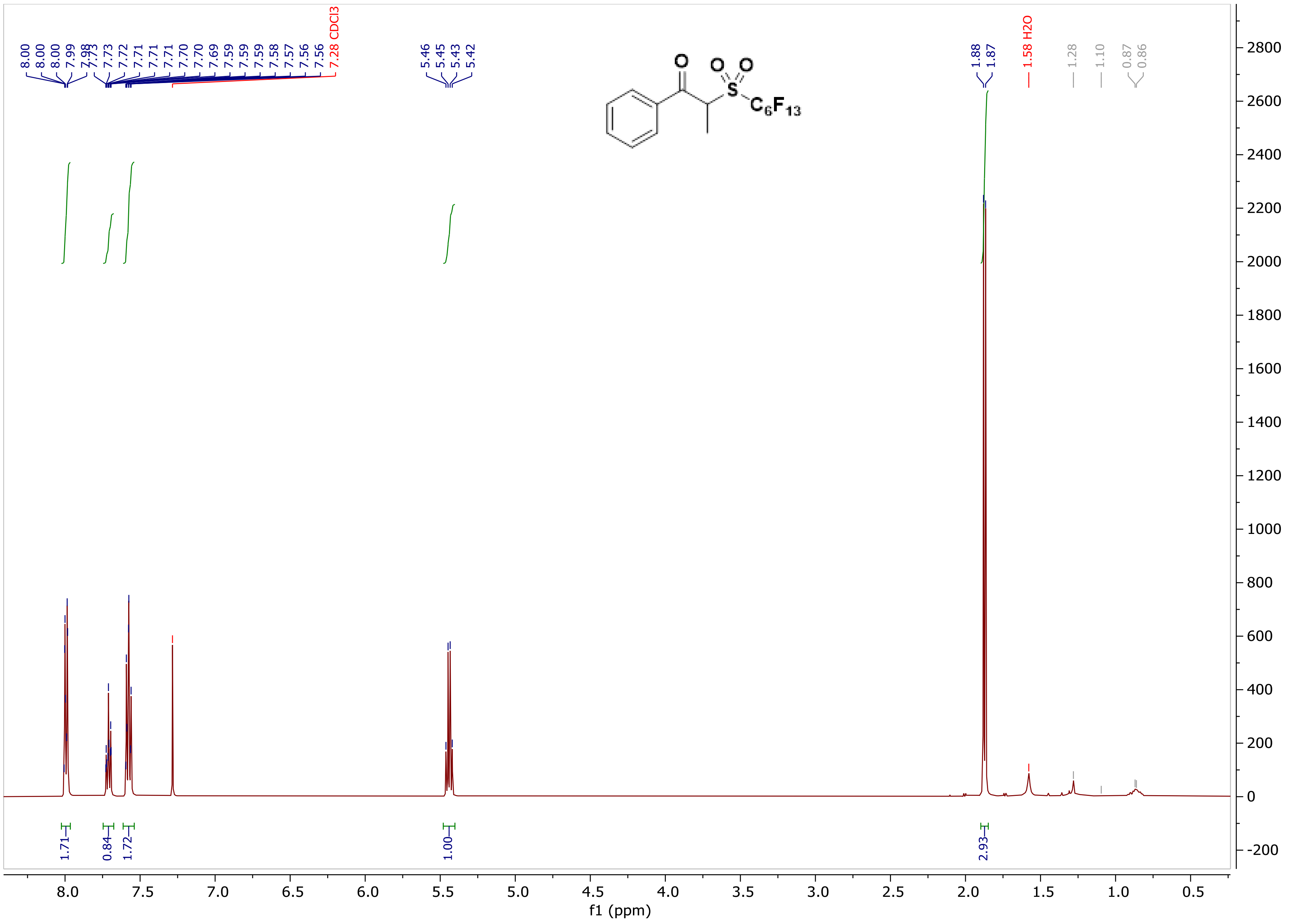

^{13}C NMR of **1b**, $CDCl_3$, 126 MHz

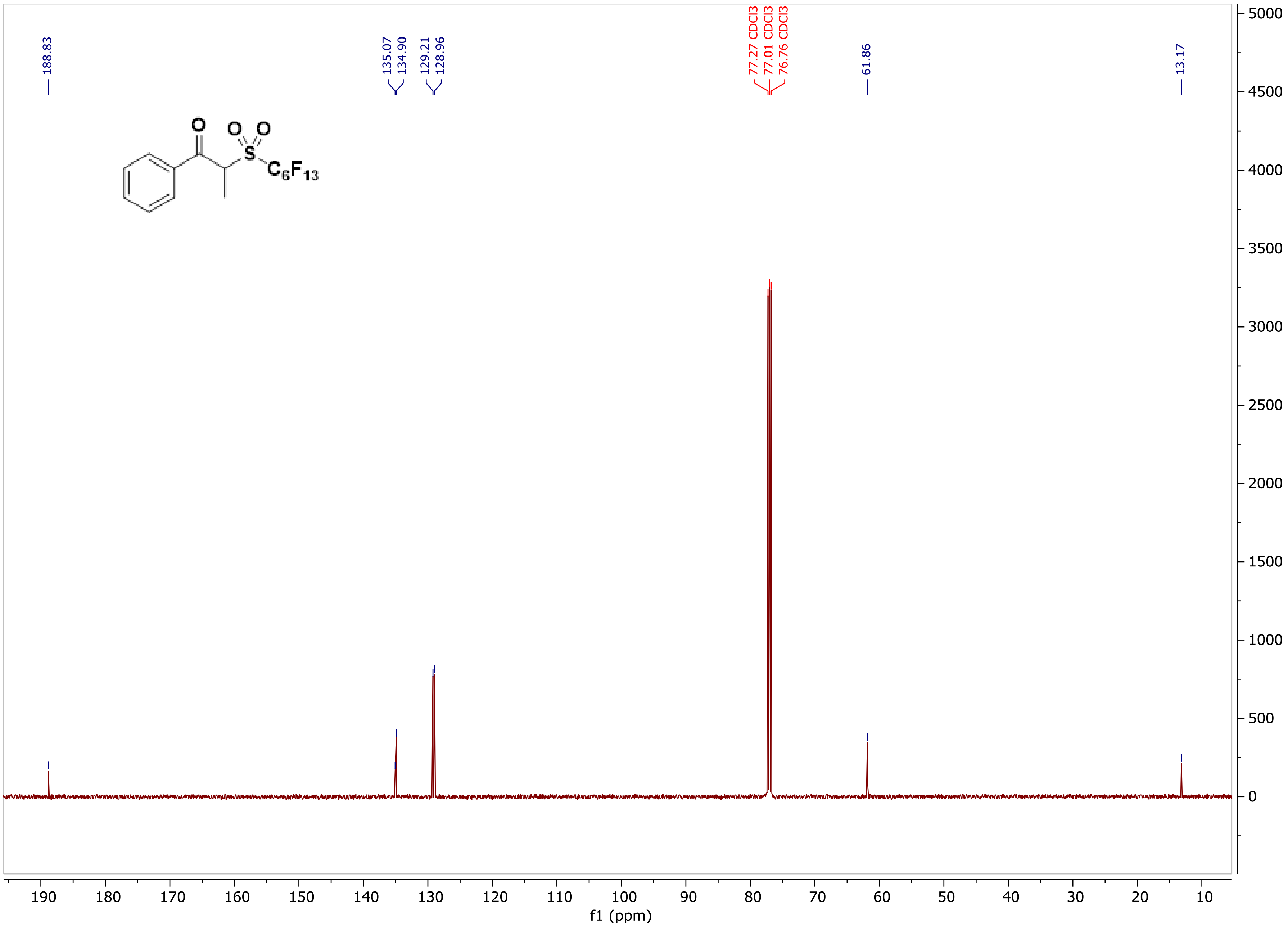

^{19}F NMR of **1b**, $CDCl_3$, 471 MHz

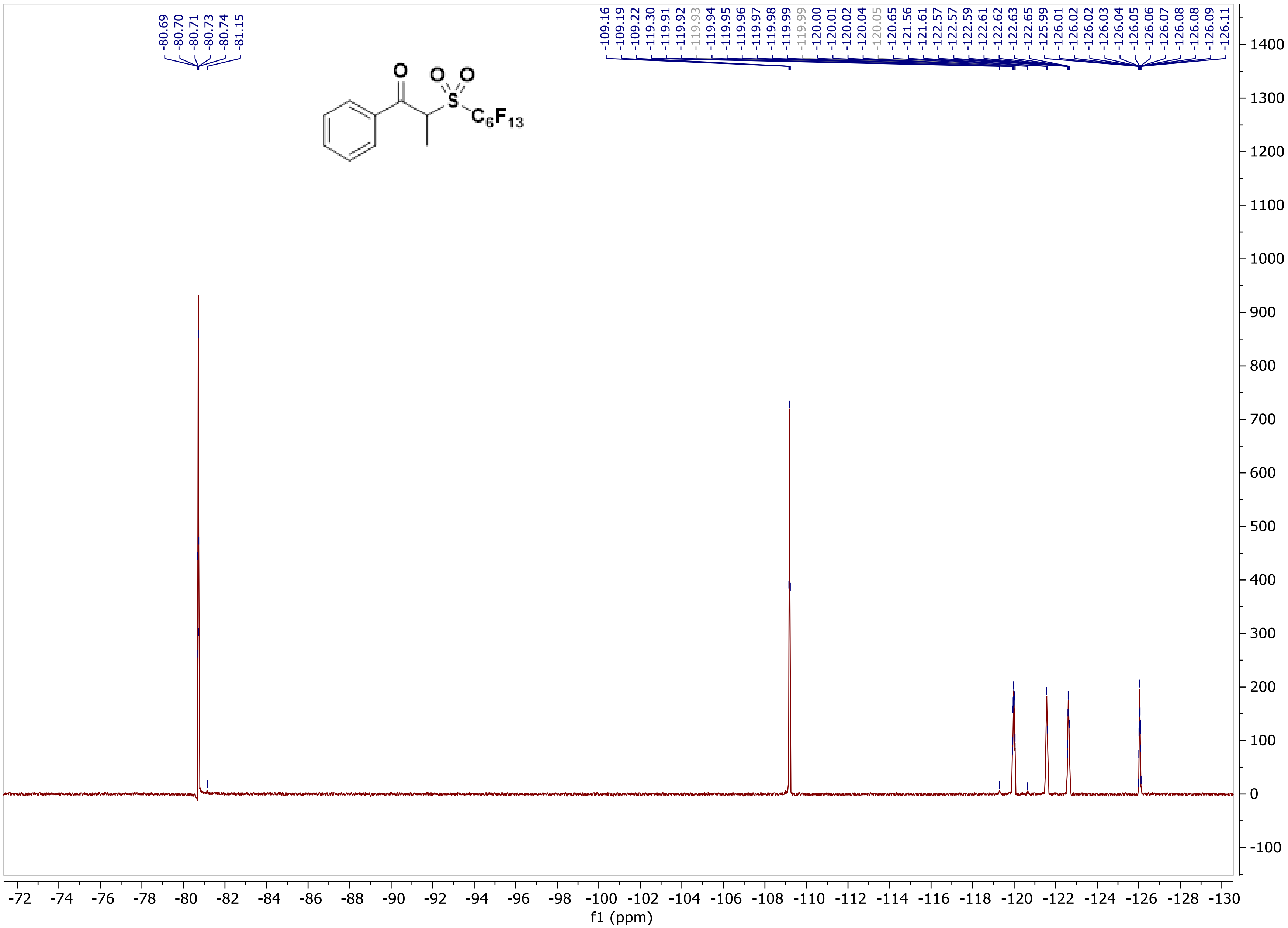

^{1}H-^{19}F HMBC of **1b**

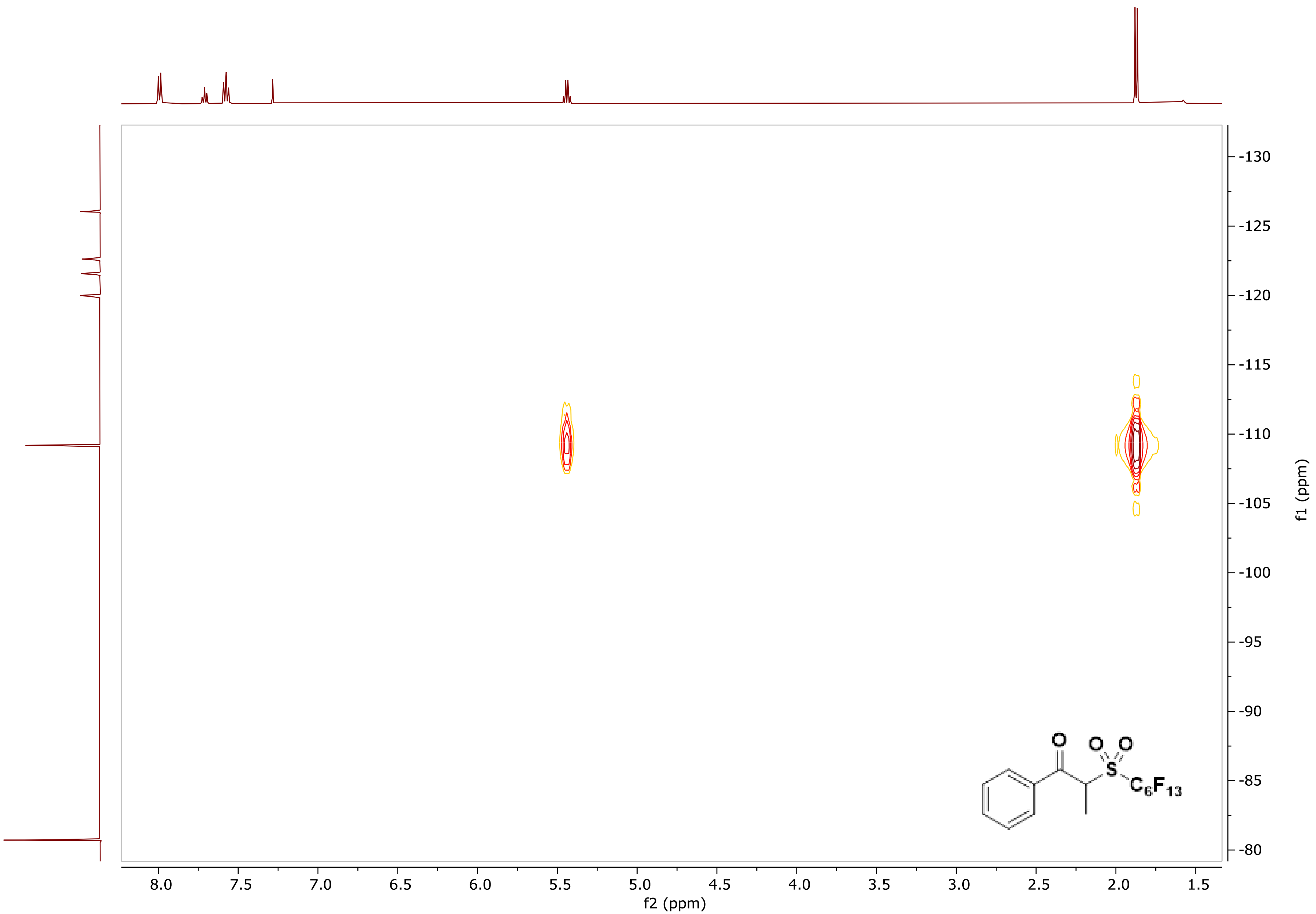

^{1}H NMR of **1a**, acetone-d_6, 500 MHz

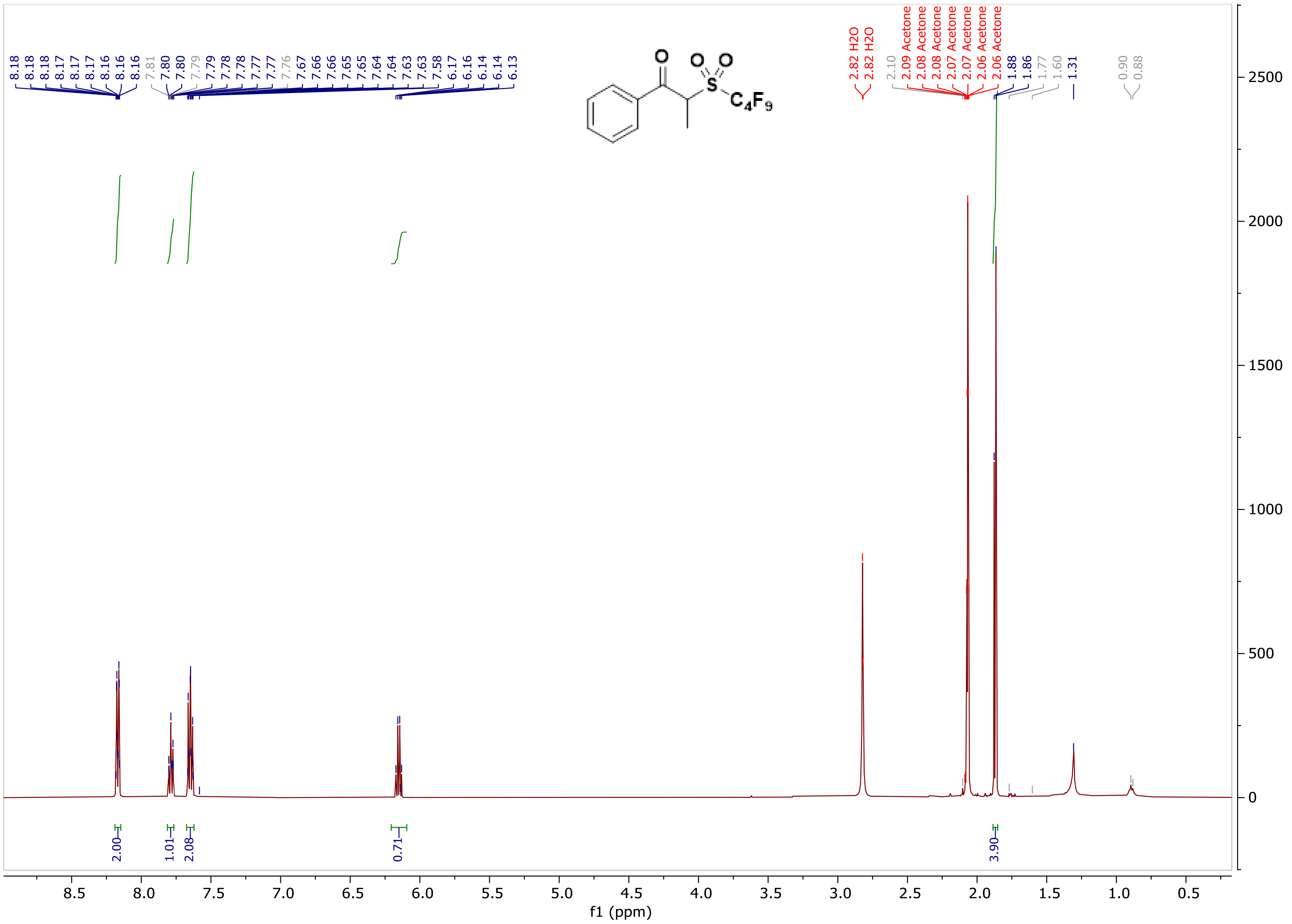

^{19}F NMR of **1a**, acetone-d_6, 471 MHz

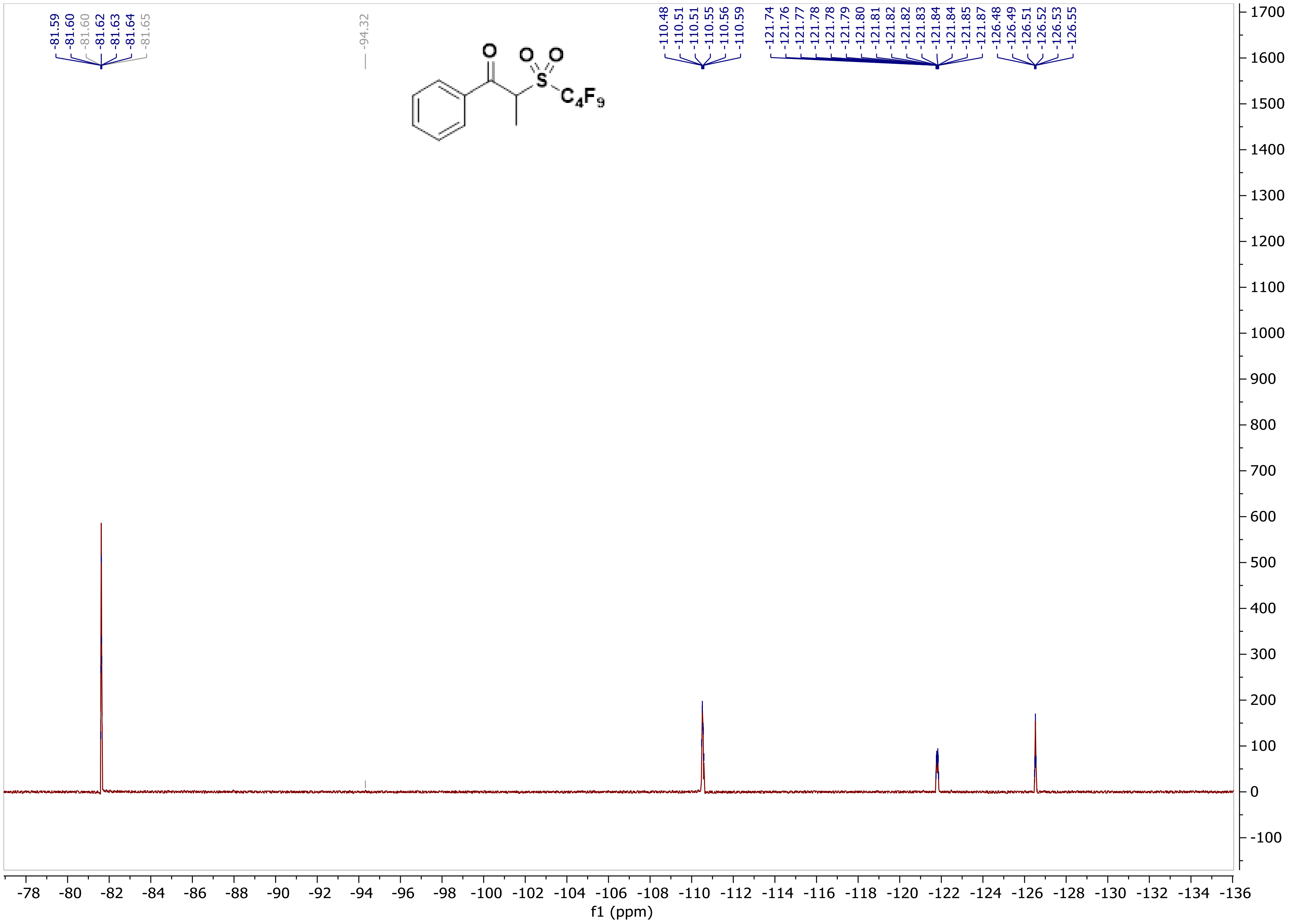

^{1}H-^{19}F HMBC of **1a**

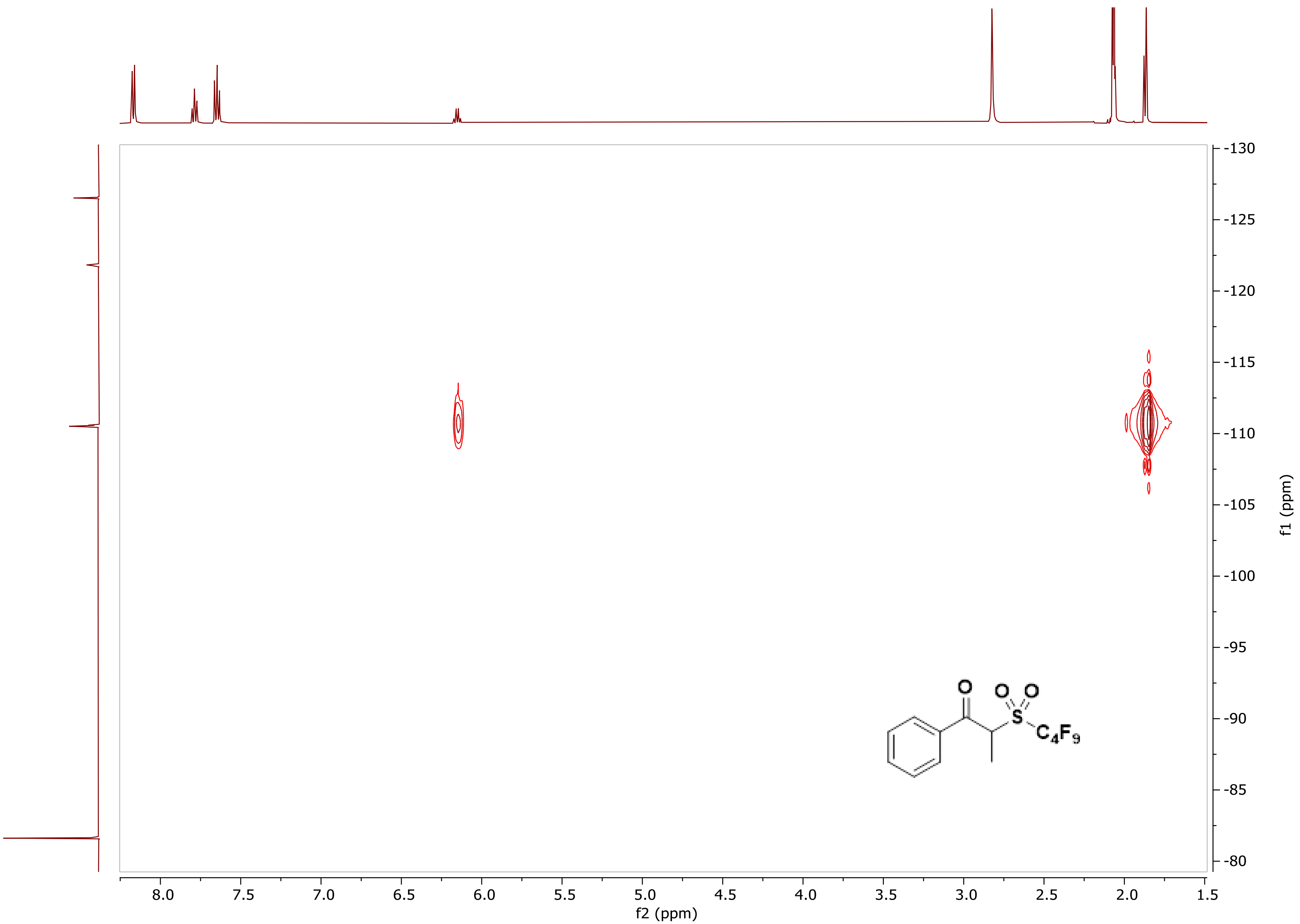

^{1}H NMR of **10c**, $CDCl_3$, 500 MHz

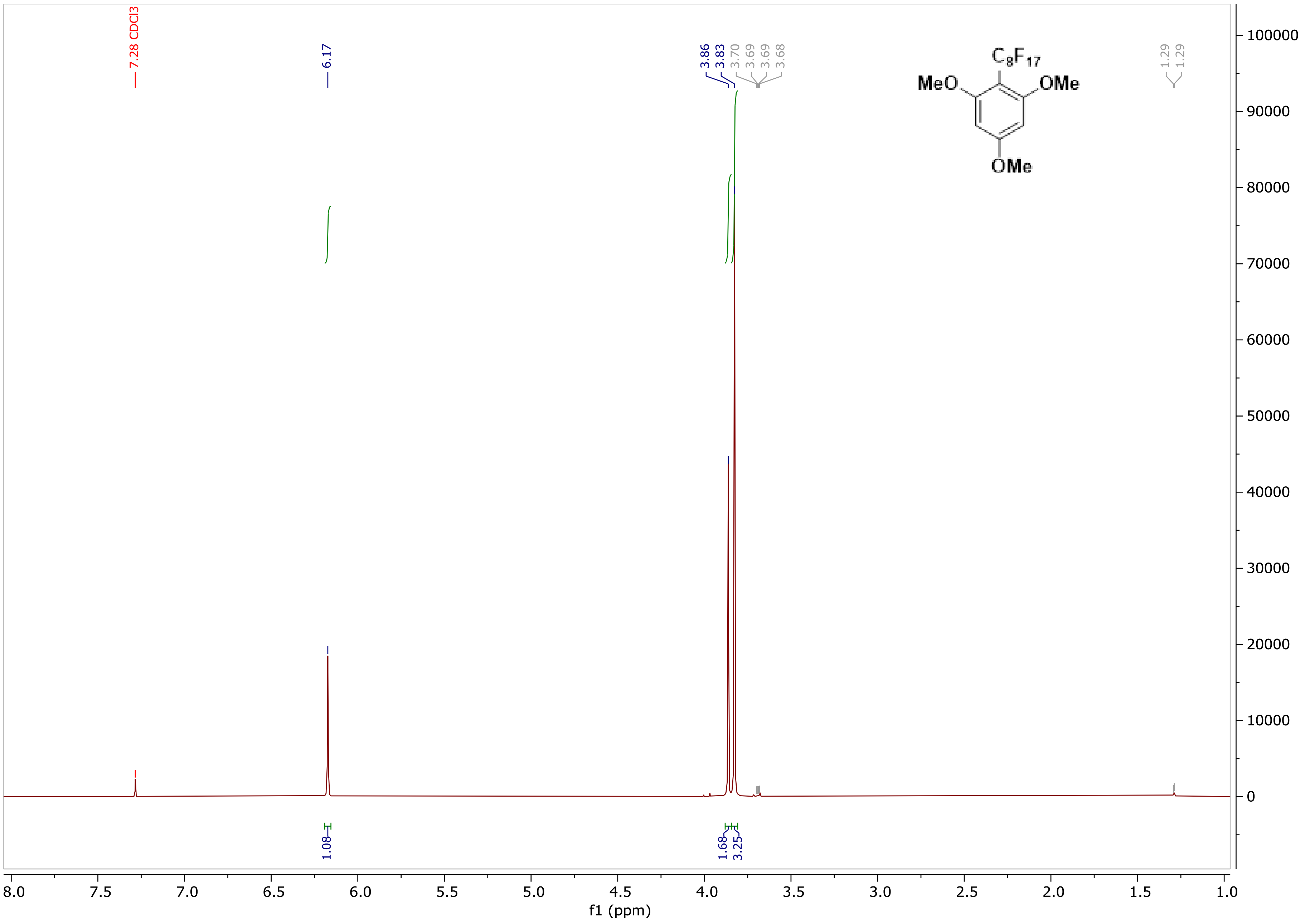

^{13}C NMR of **10c**, $CDCl_3$, 126 MHz

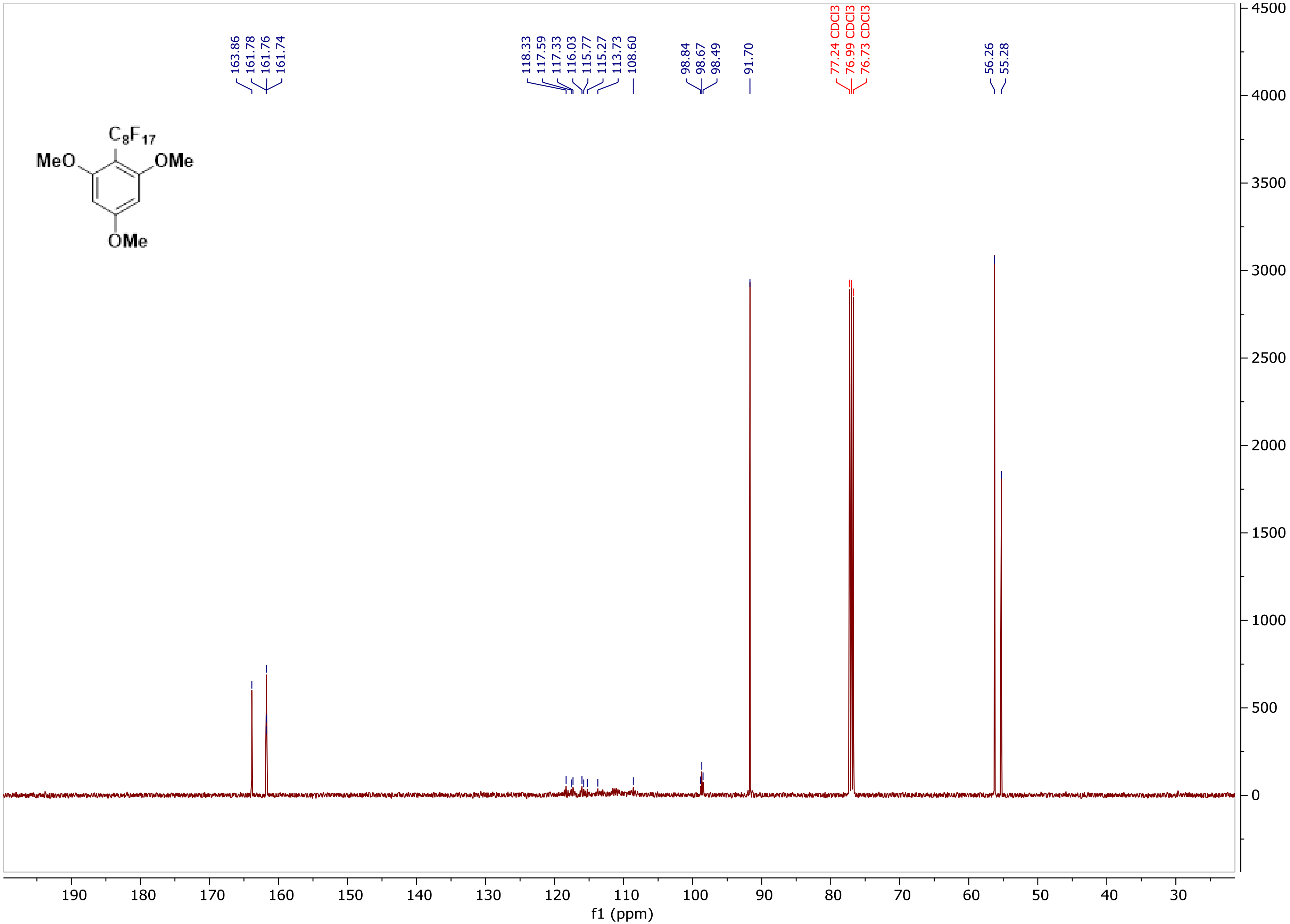

^{19}F NMR of **10c**, $CDCl_3$, 471 MHz

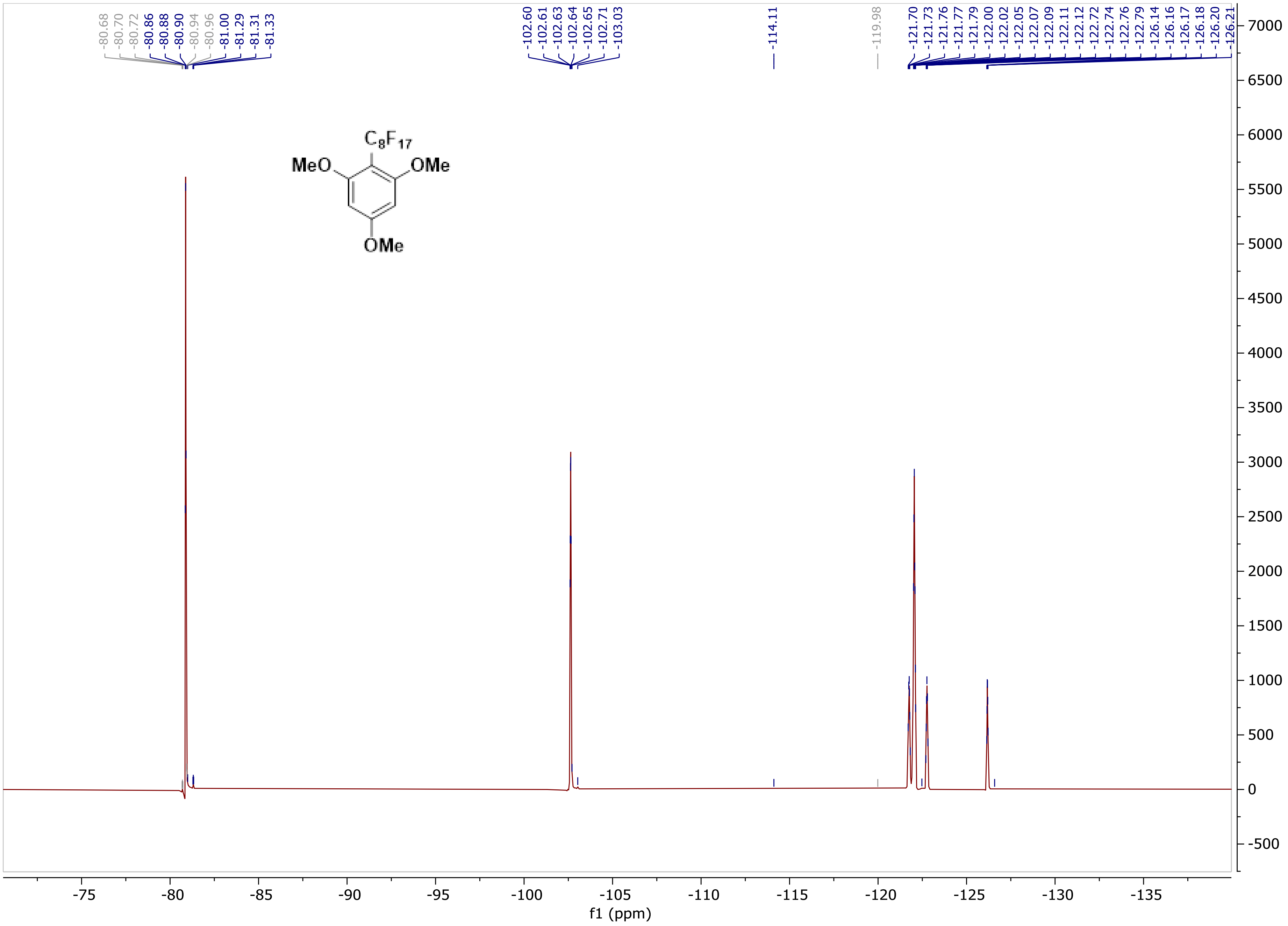

^{1}H NMR of **10b**, $CDCl_3$, 500 MHz

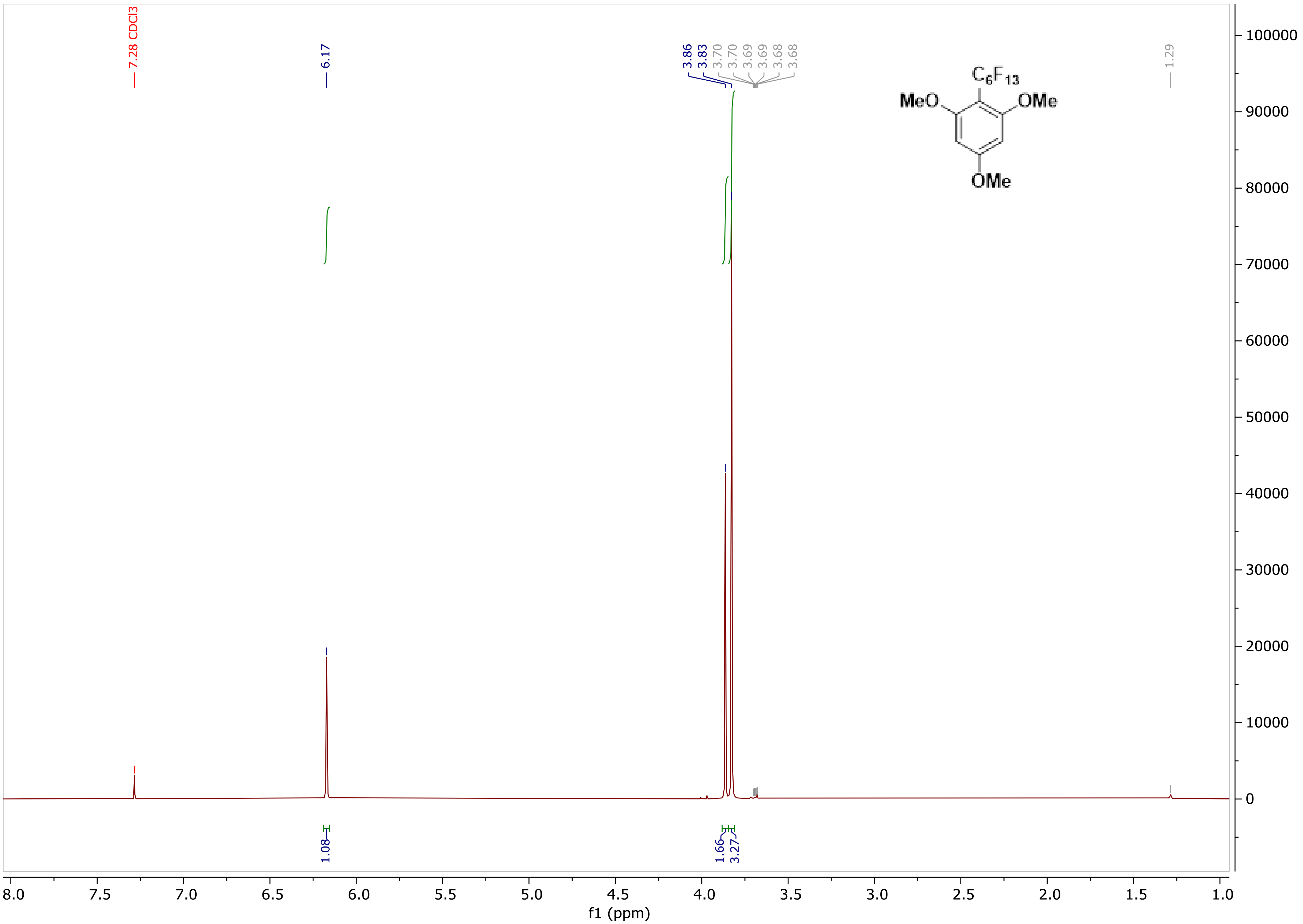

^{13}C NMR of **10b**, $CDCl_3$, 126 MHz

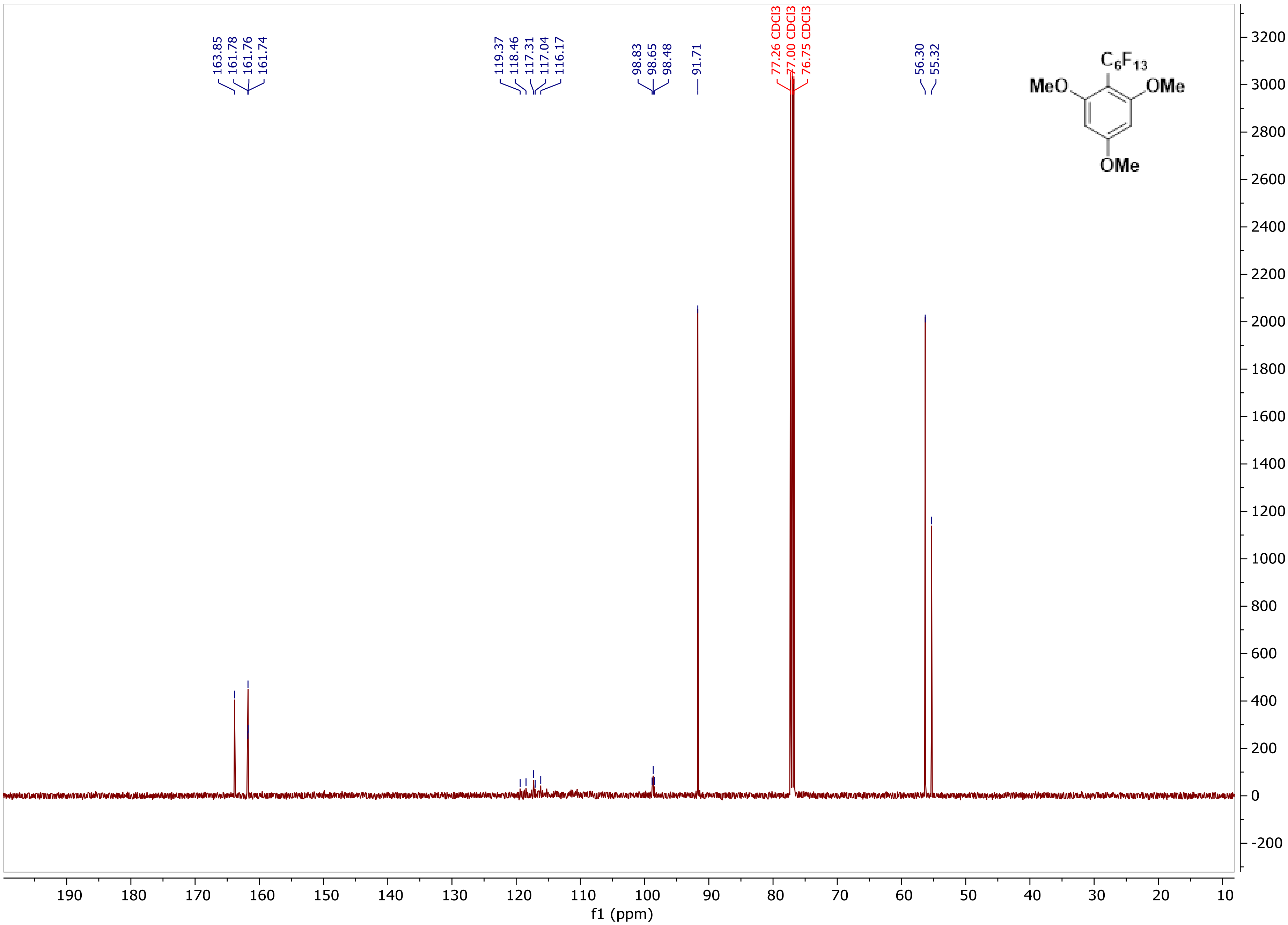

^{19}F NMR of **10b**, $CDCl_3$, 471 MHz

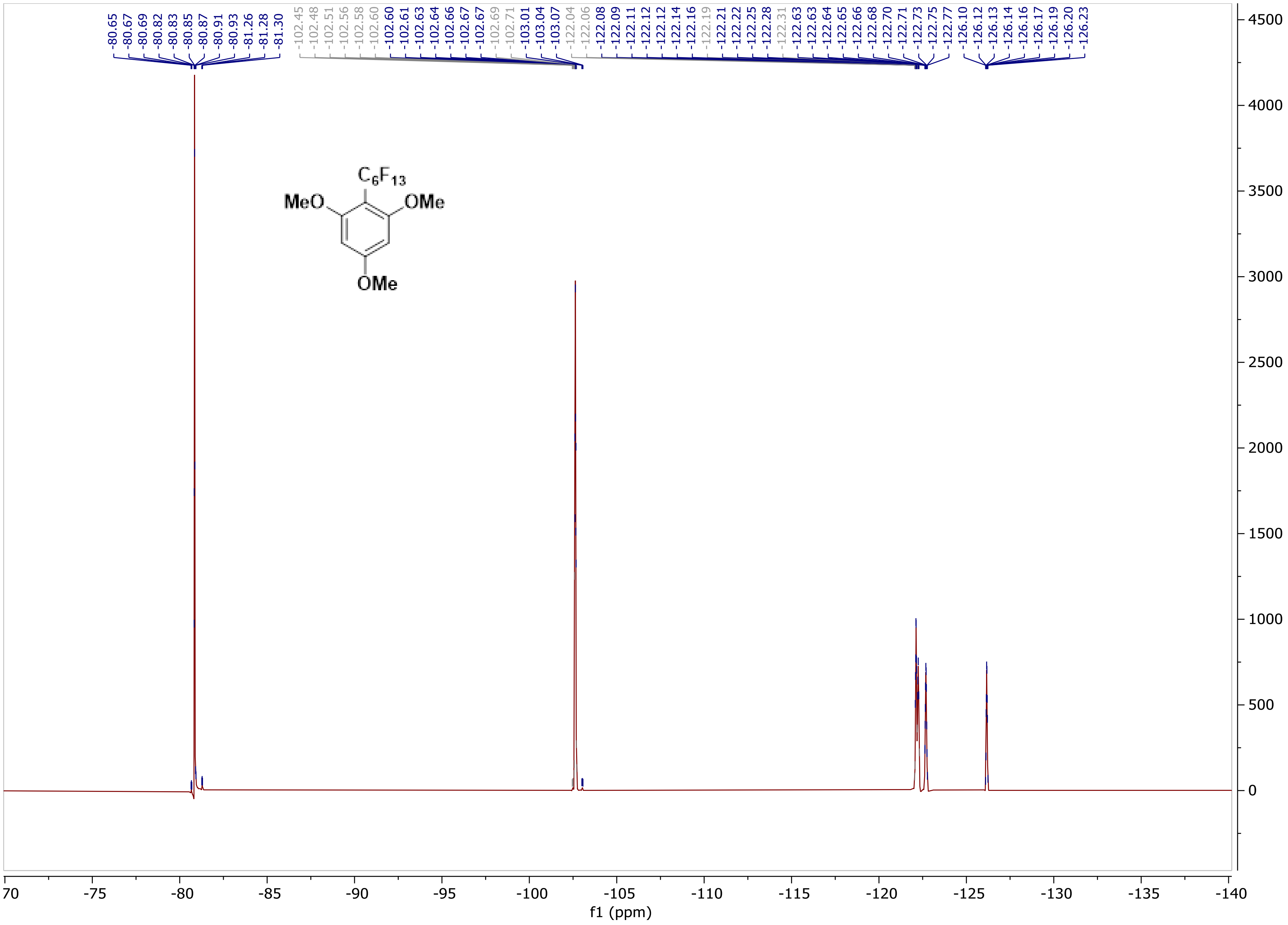

^1H NMR of **10a**, $CDCl_3$, 500 MHz

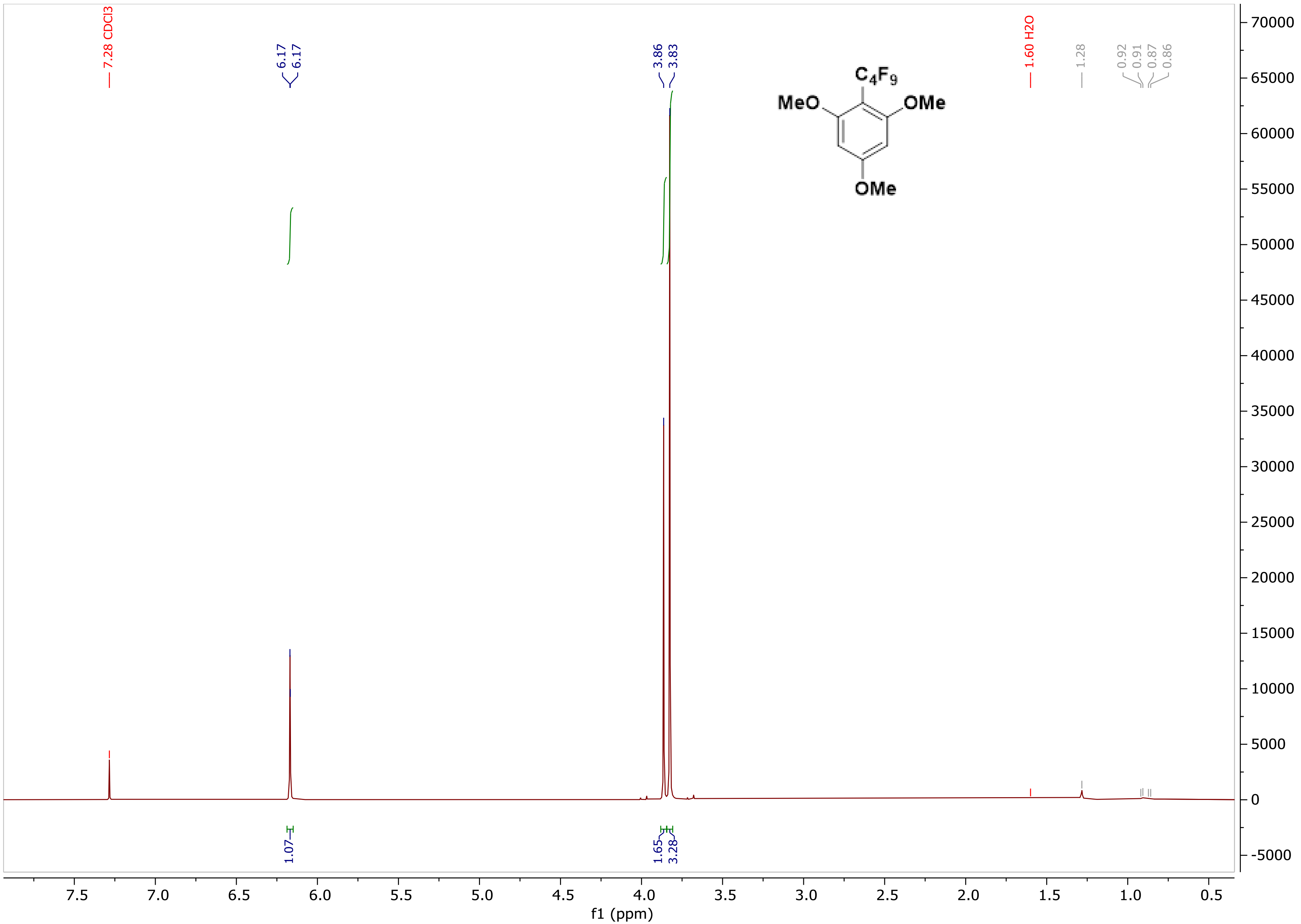

^{13}C NMR of **10a**, $CDCl_3$, 126 MHz

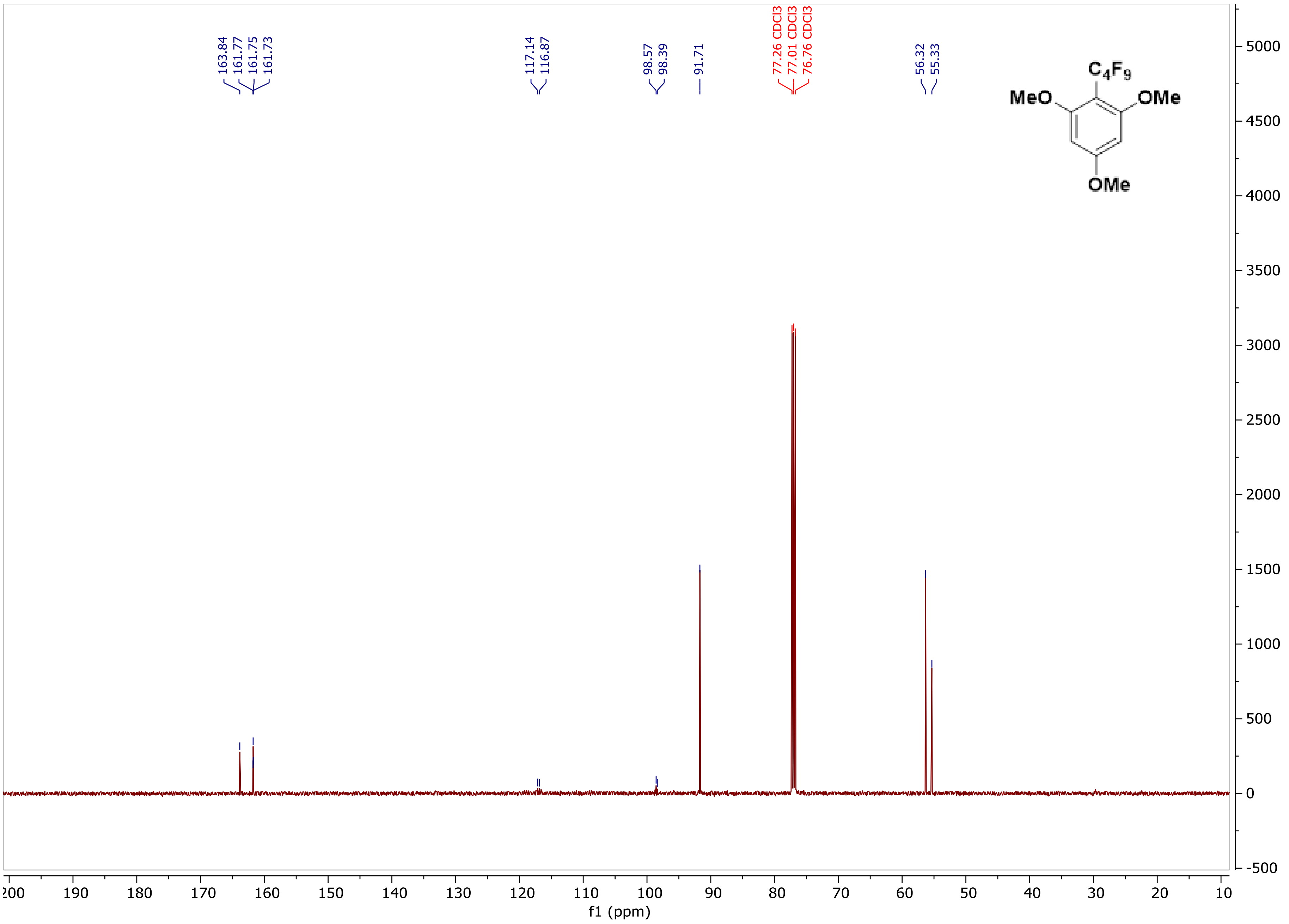

^{19}F NMR of **10a**, $CDCl_3$, 471 MHz

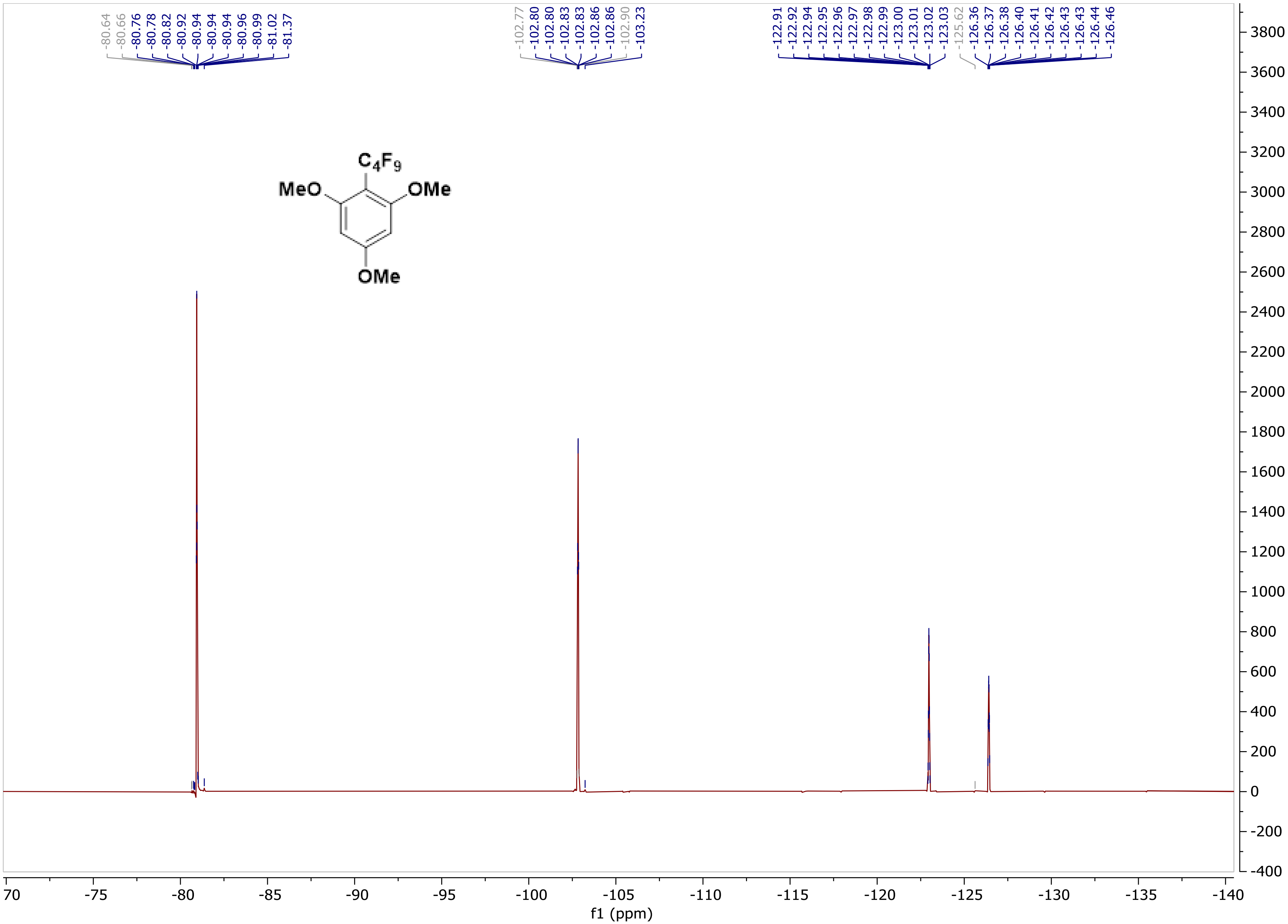

^{1}H NMR of **11b**, $CDCl_3$, 500 MHz

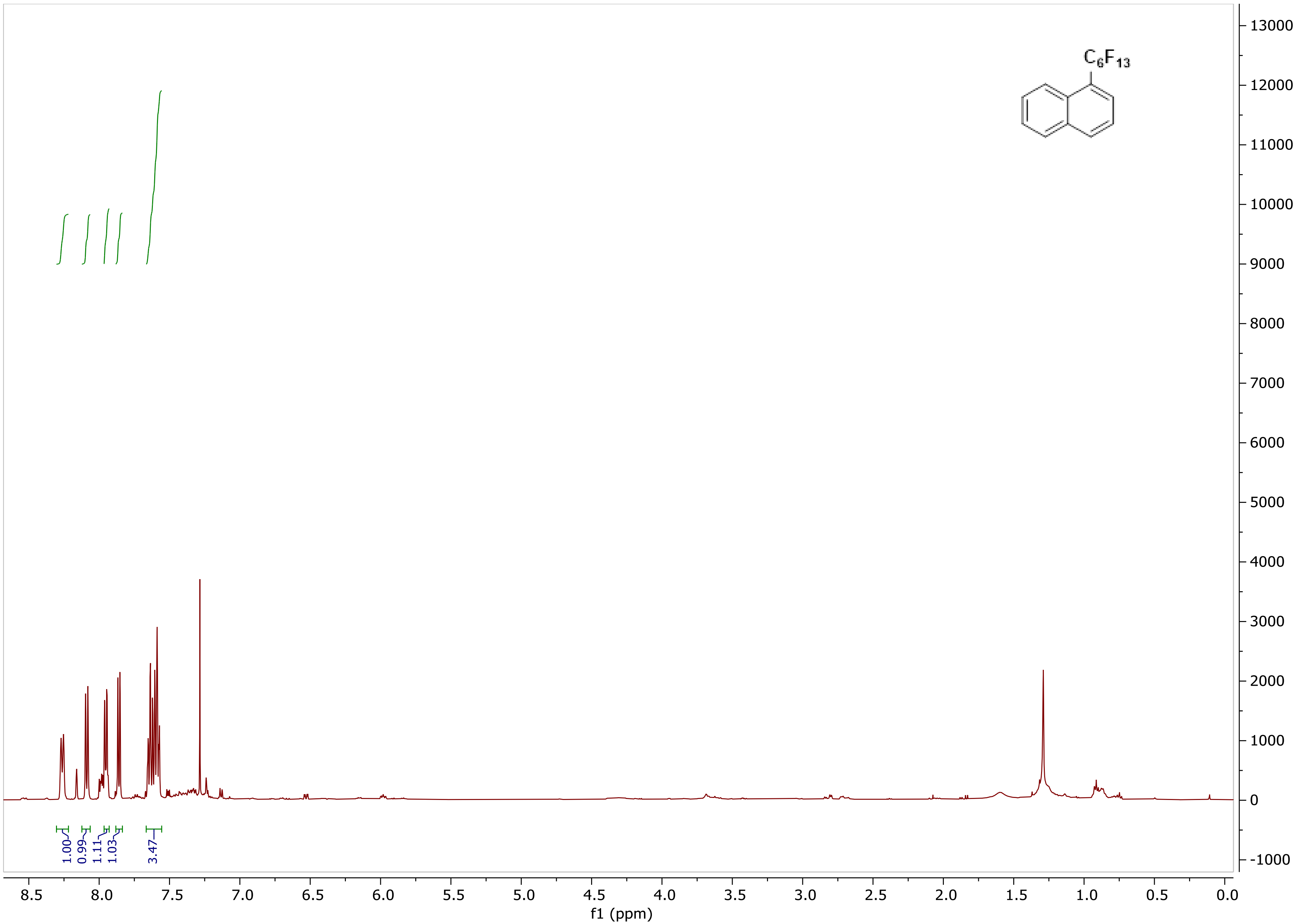

^{13}C NMR of **11b**, $CDCl_3$, 126 MHz

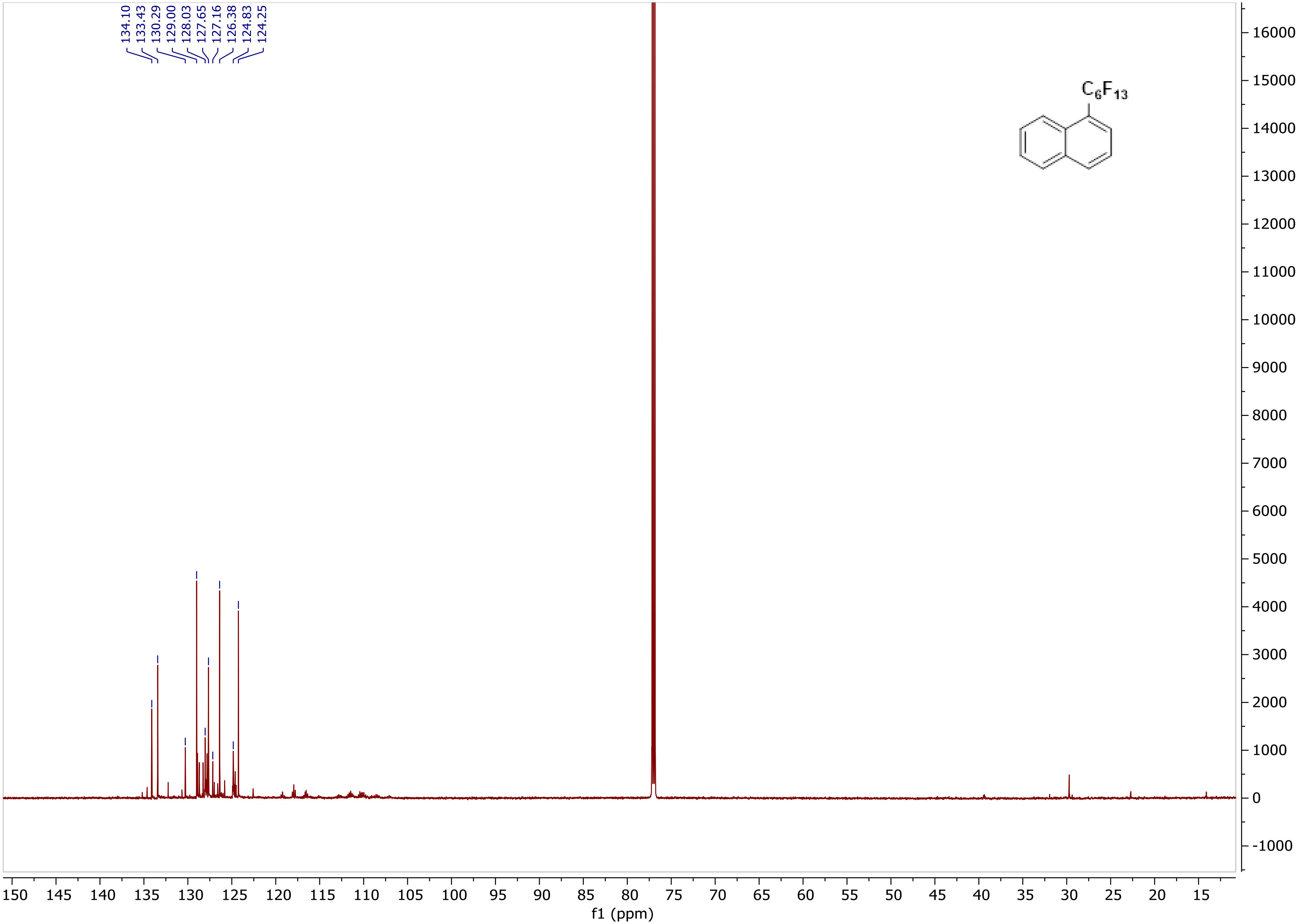

^{19}F NMR of **11b**, $CDCl_3$, 471 MHz

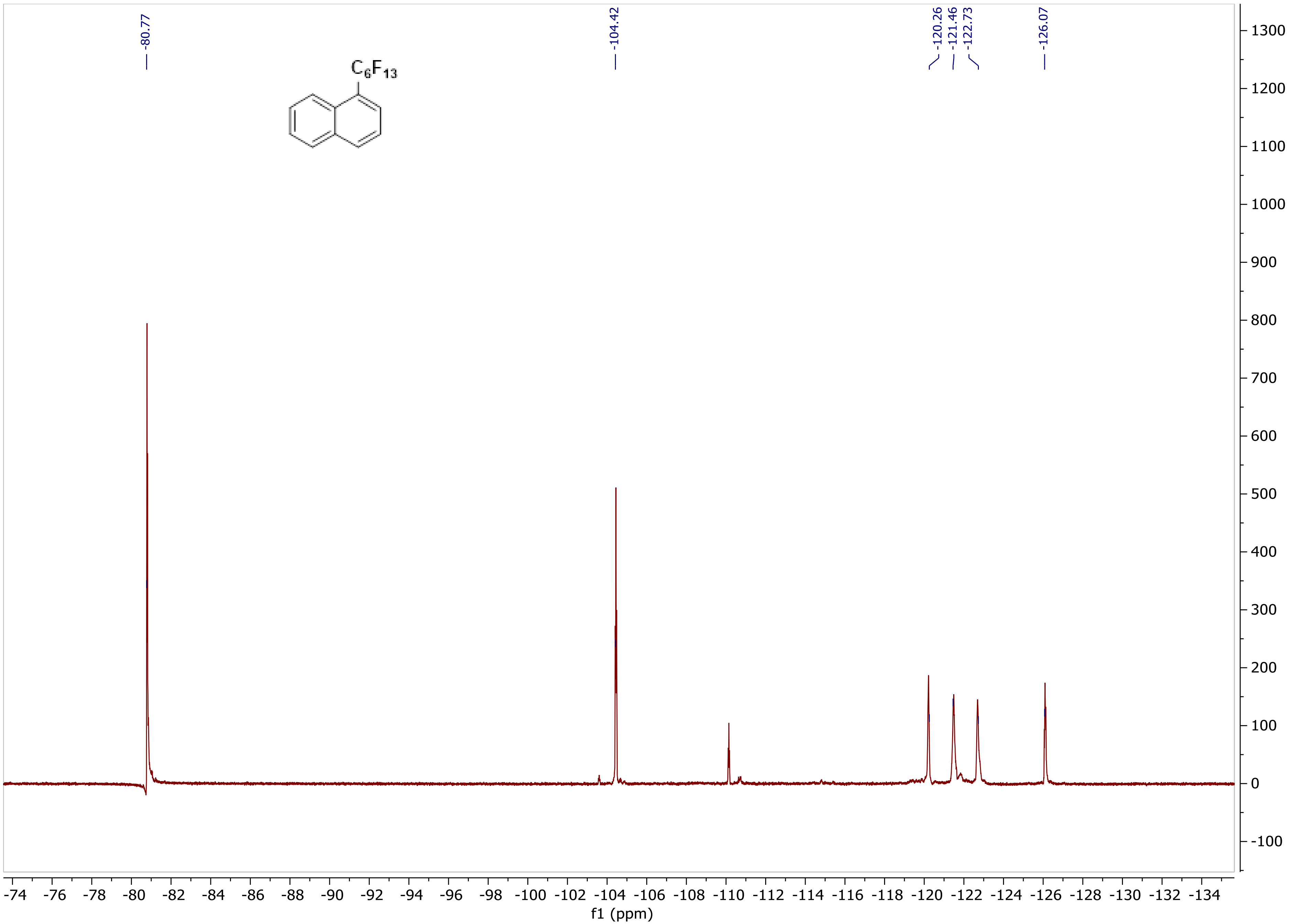

^{1}H-^{19}F HMBC of **11b**

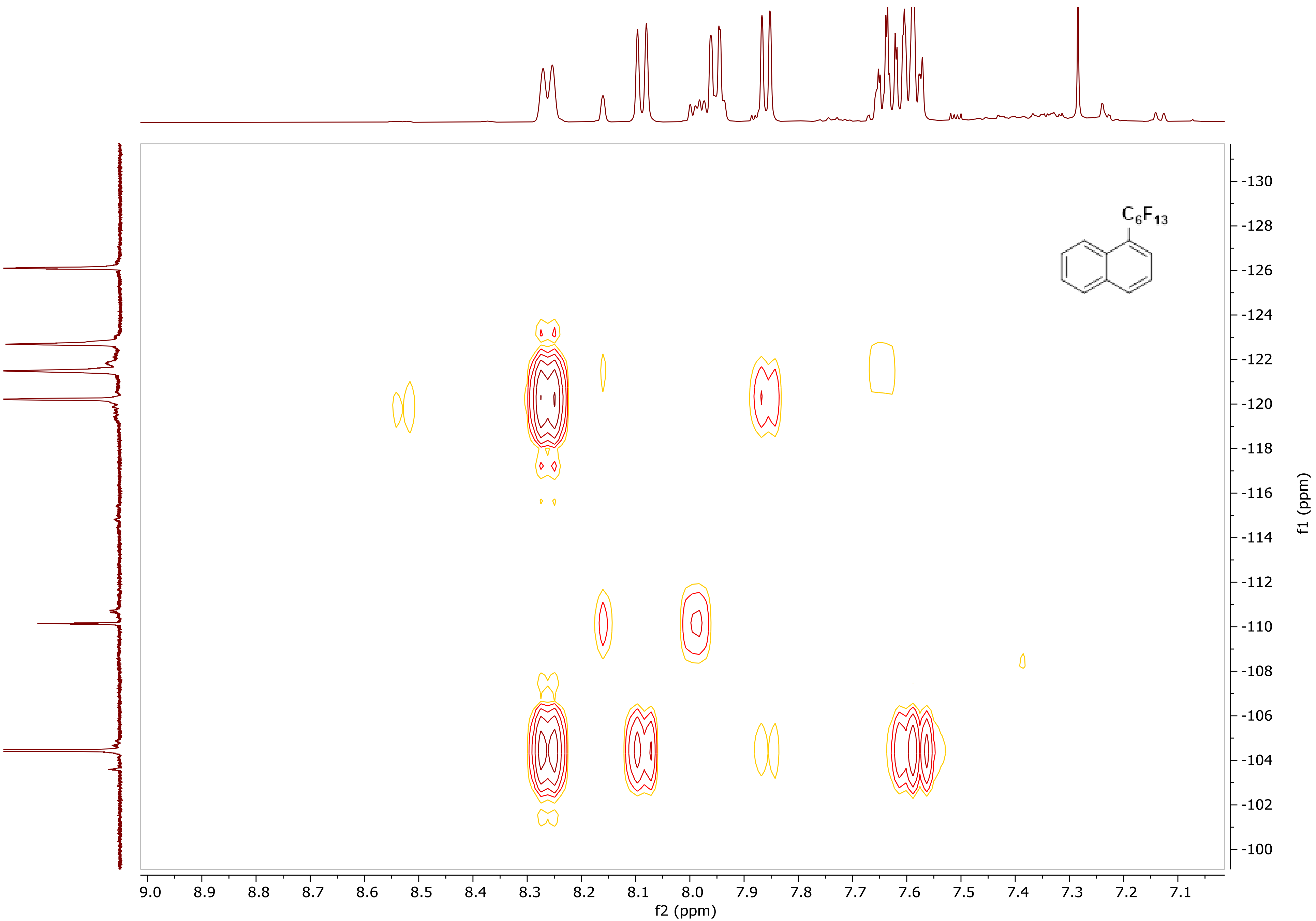

^{1}H NMR of **12c**, $CDCl_3$, 500 MHz

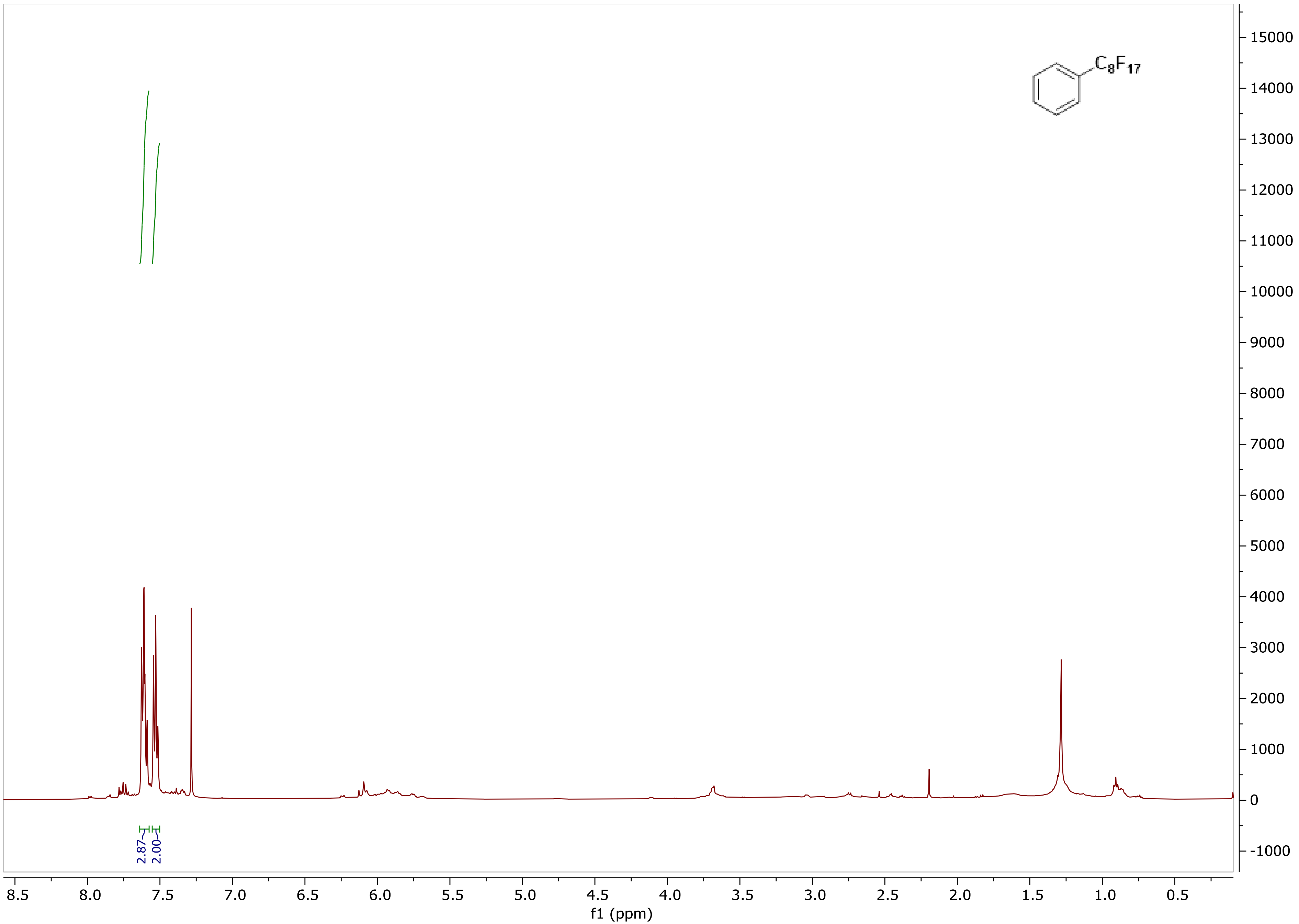

^{13}C NMR of **12c**, $CDCl_3$, 126 MHz

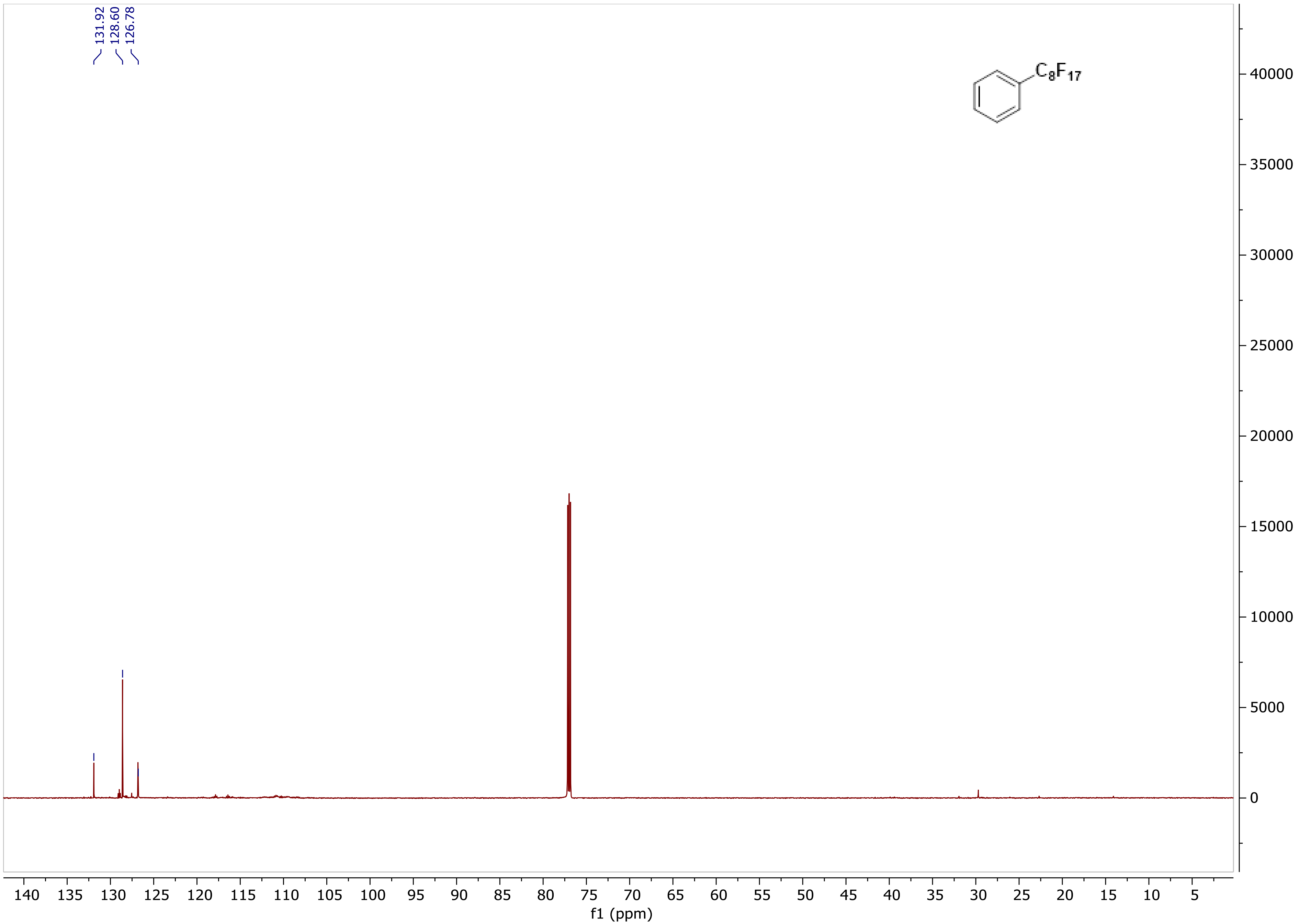

^{19}F NMR of **12c**, $CDCl_3$, 471 MHz

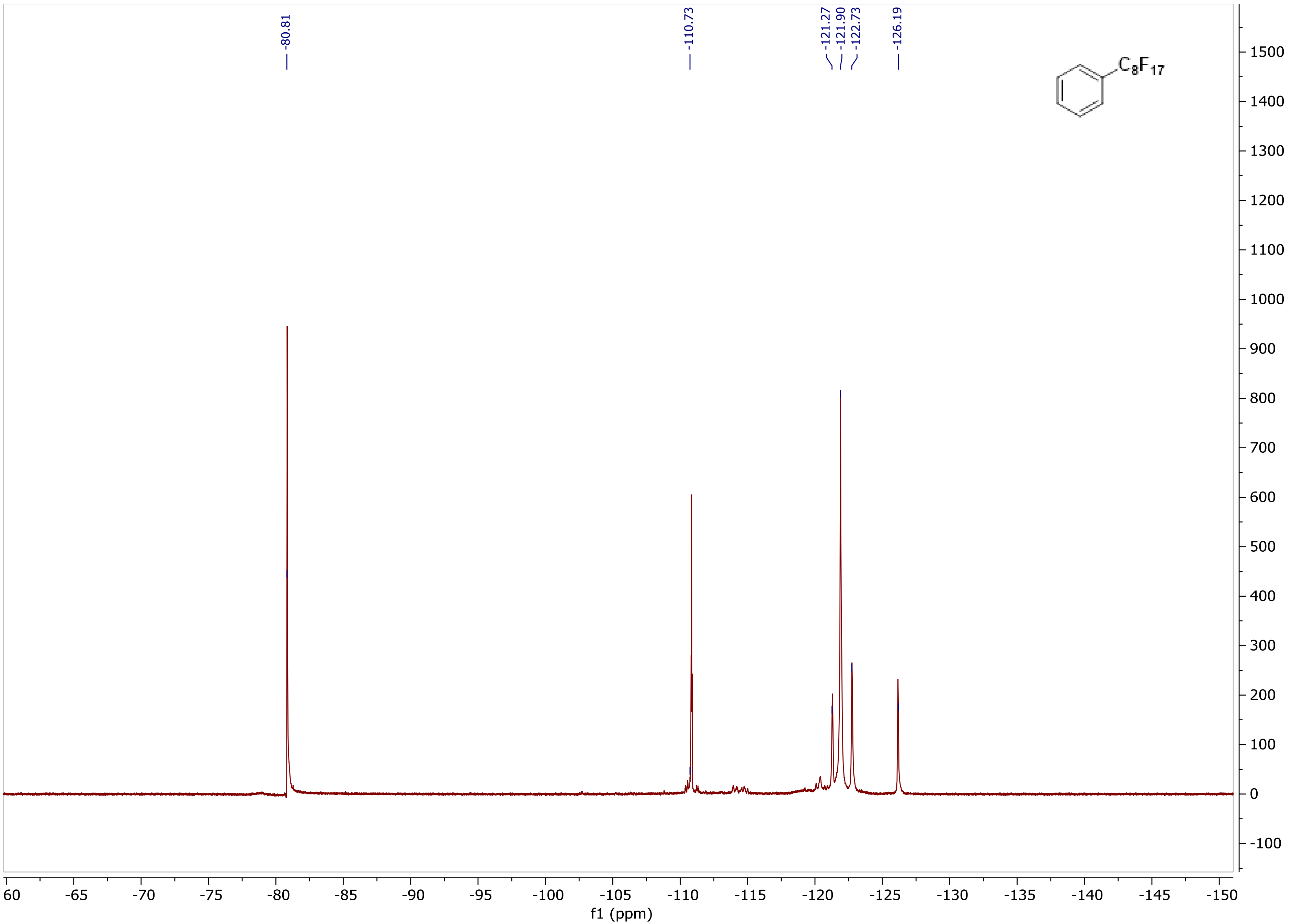

^{1}H-^{19}F HMBC of **12c**

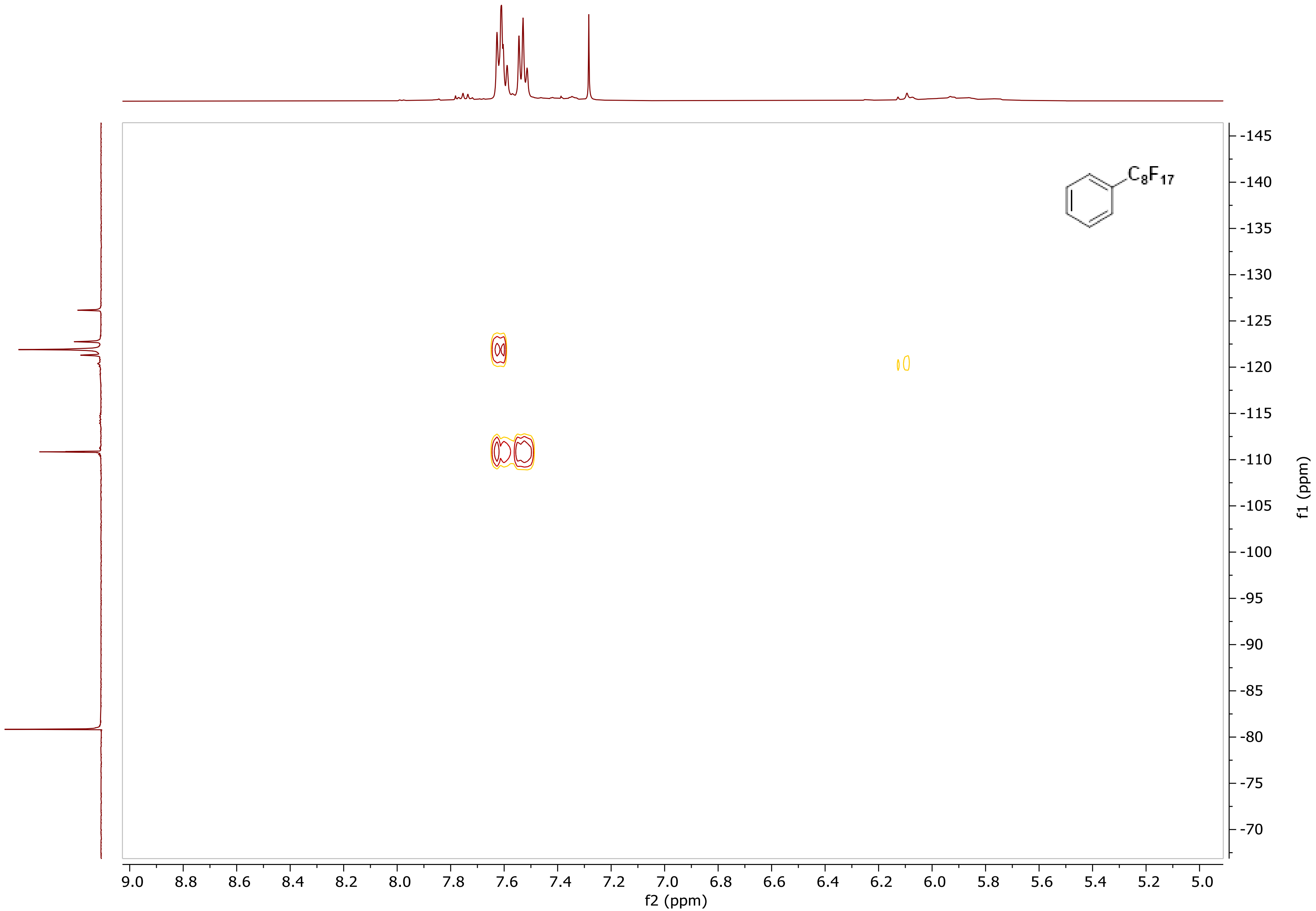

^{1}H NMR of **13b**, $CDCl_3$, 500 MHz

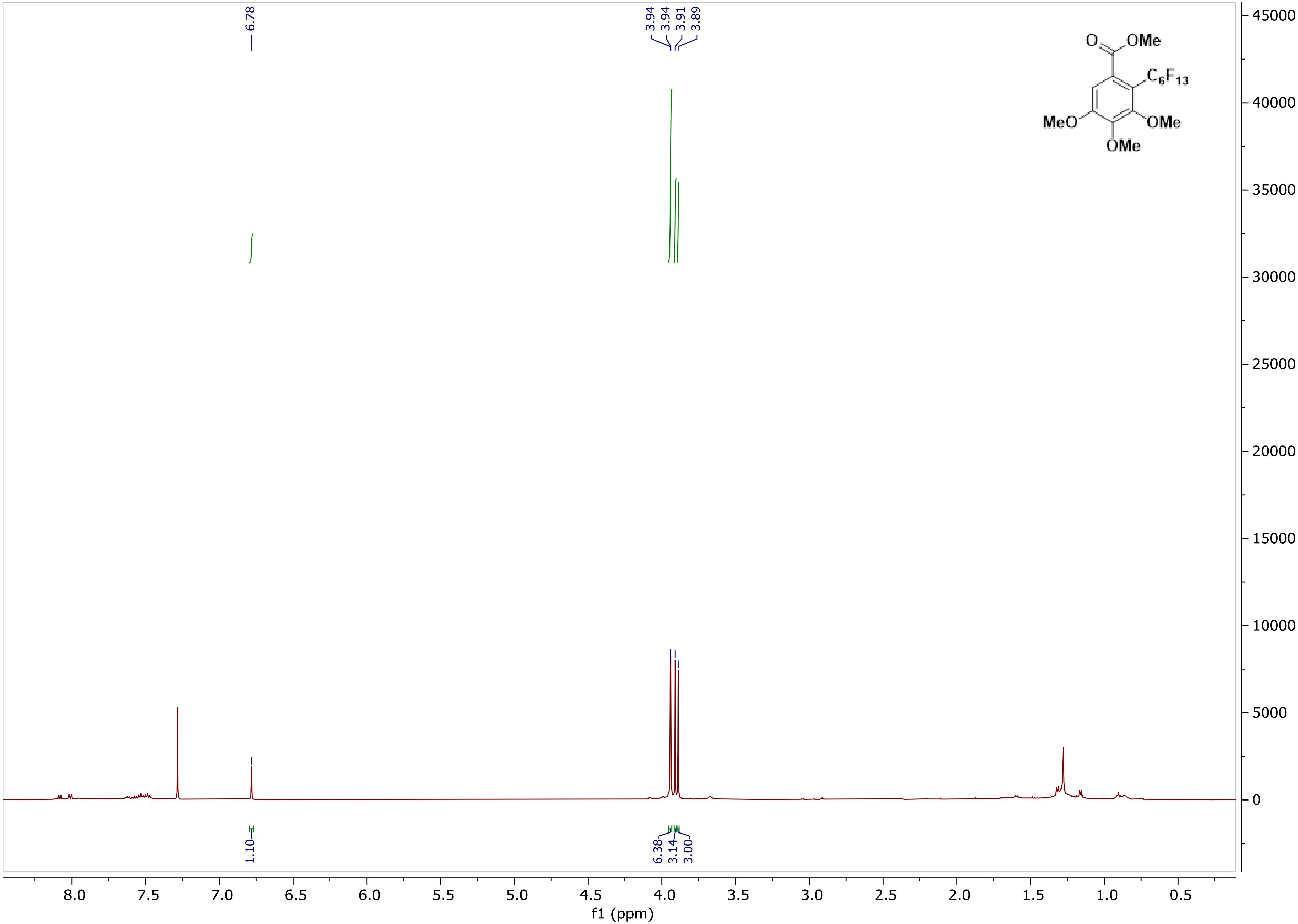

^{13}C NMR of **13b**, $CDCl_3$, 126 MHz

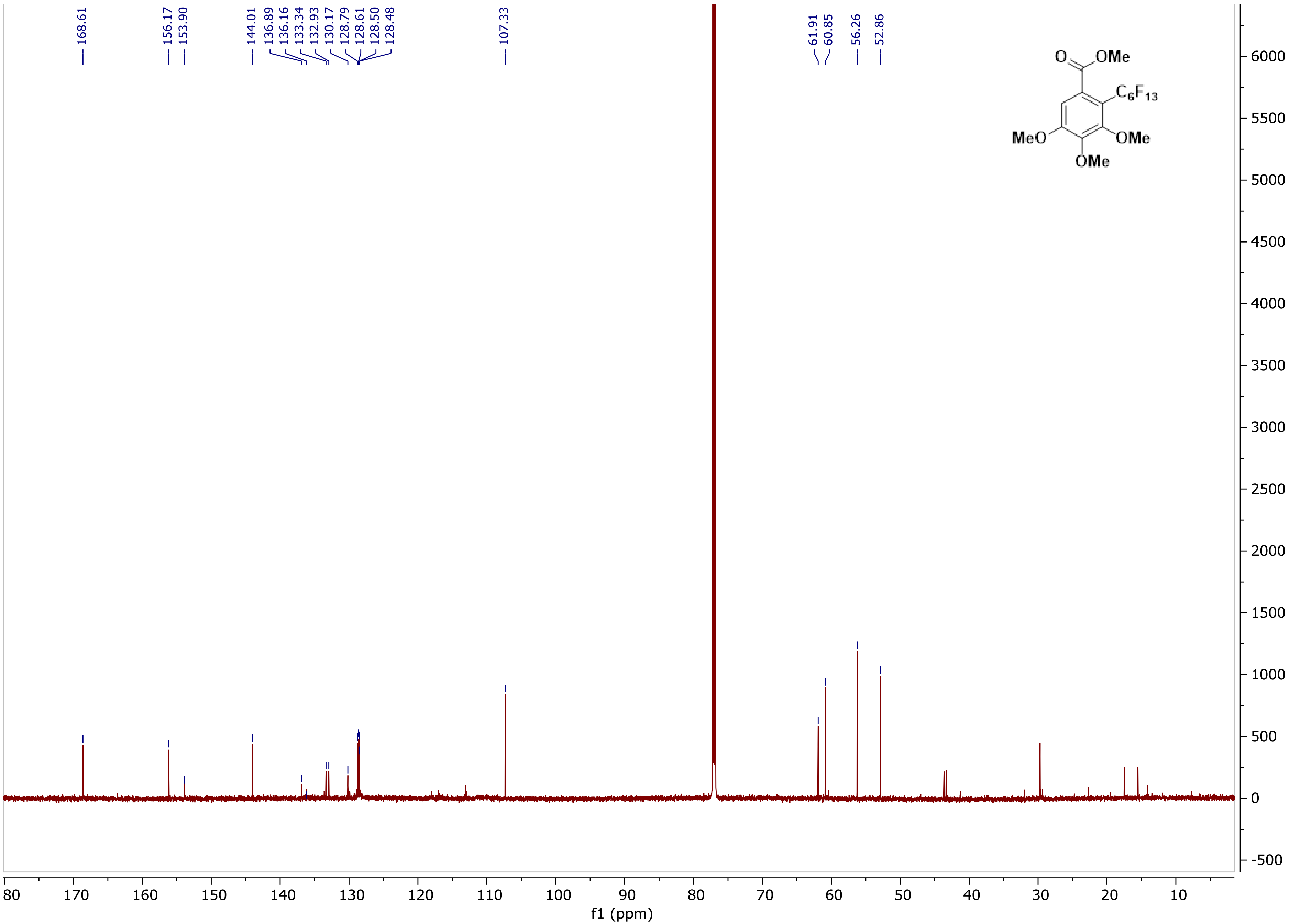

^{19}F NMR of **13b**, $CDCl_3$, 471 MHz

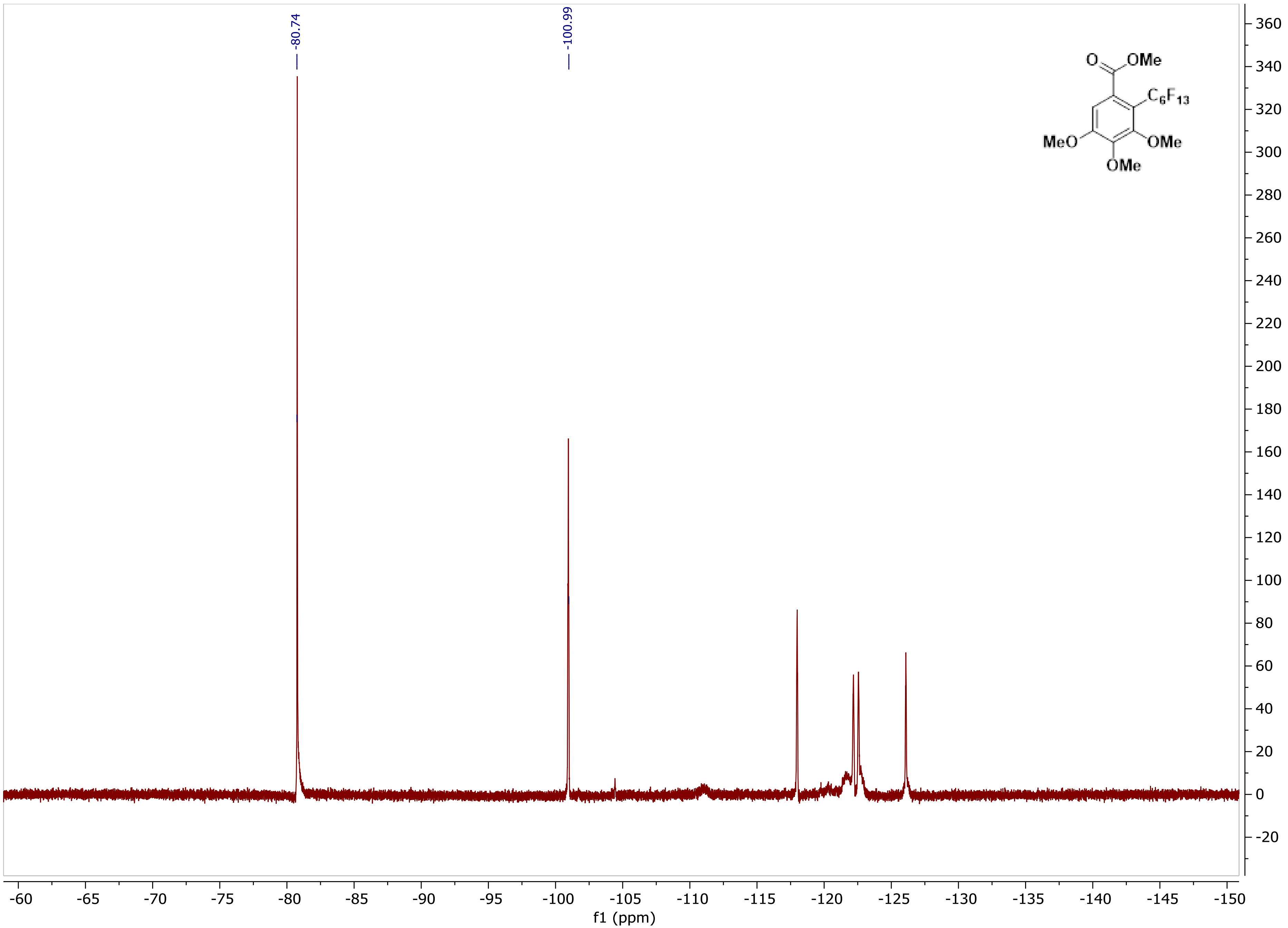

^{1}H-^{19}F HMBC of **13b**

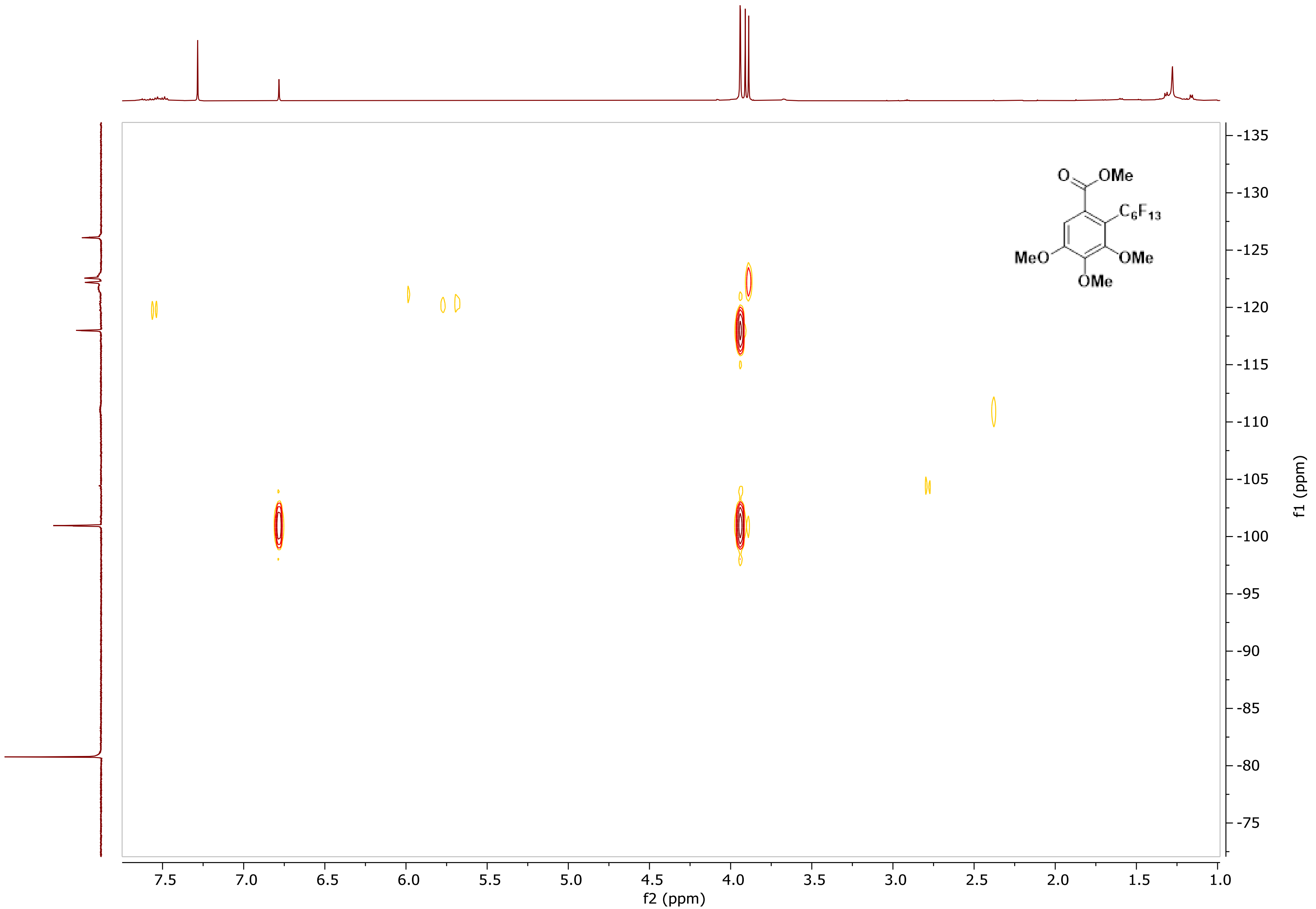

^{1}H NMR of **14b**, $CDCl_3$, 500 MHz

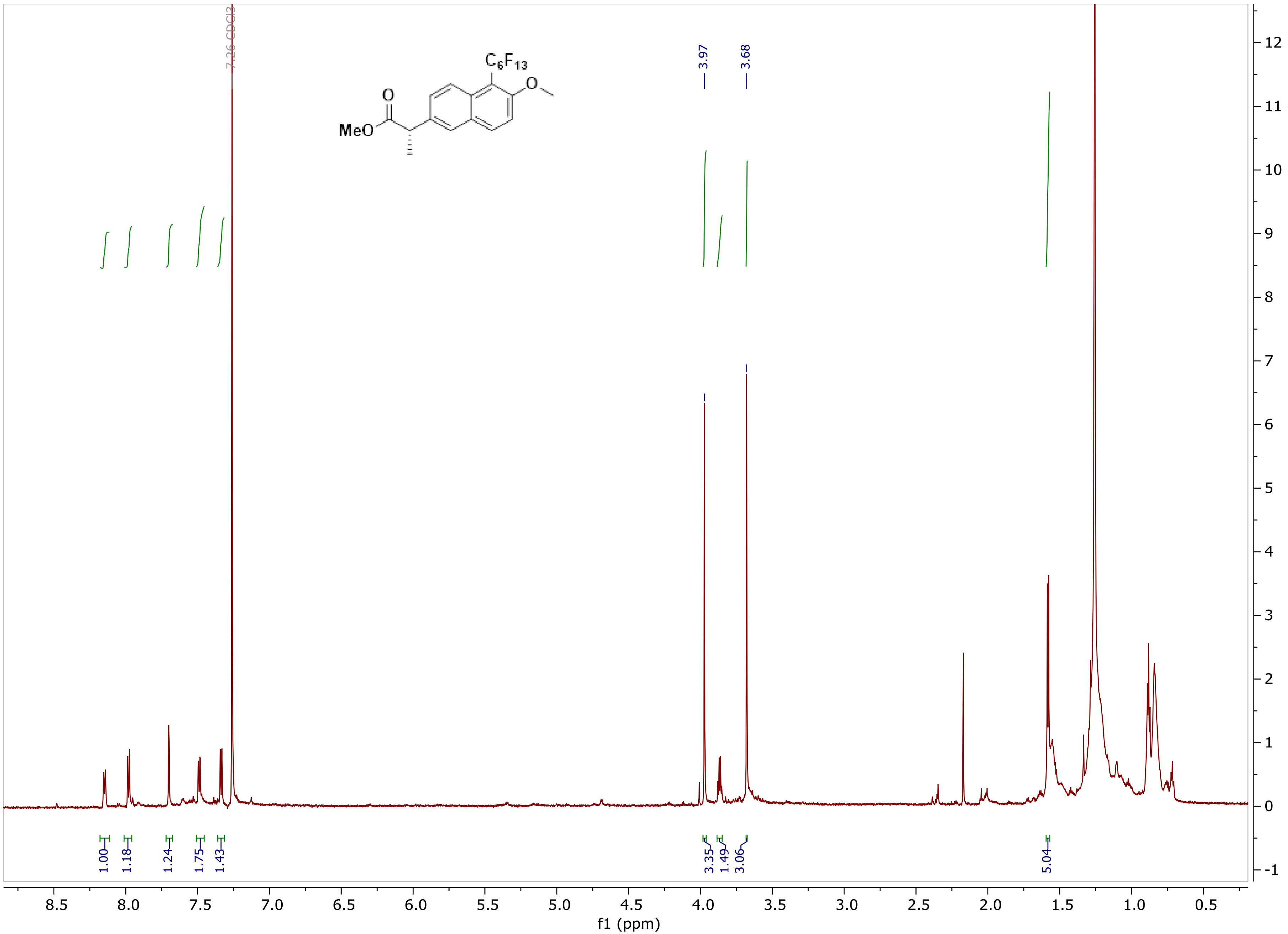

^{13}C NMR of **14b**, $CDCl_3$, 126 MHz

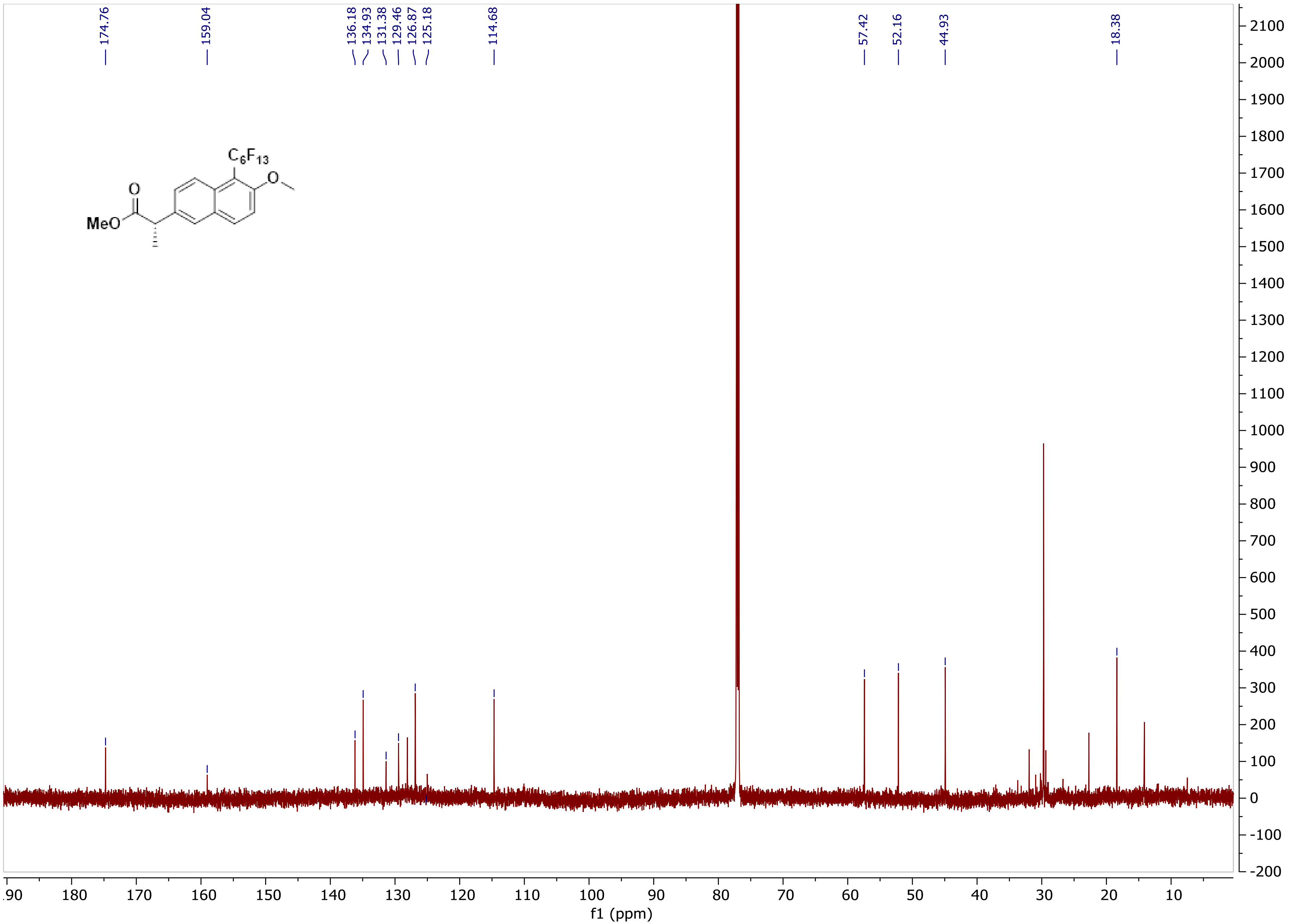

^{19}F NMR of **14b**, $CDCl_3$, 471 MHz

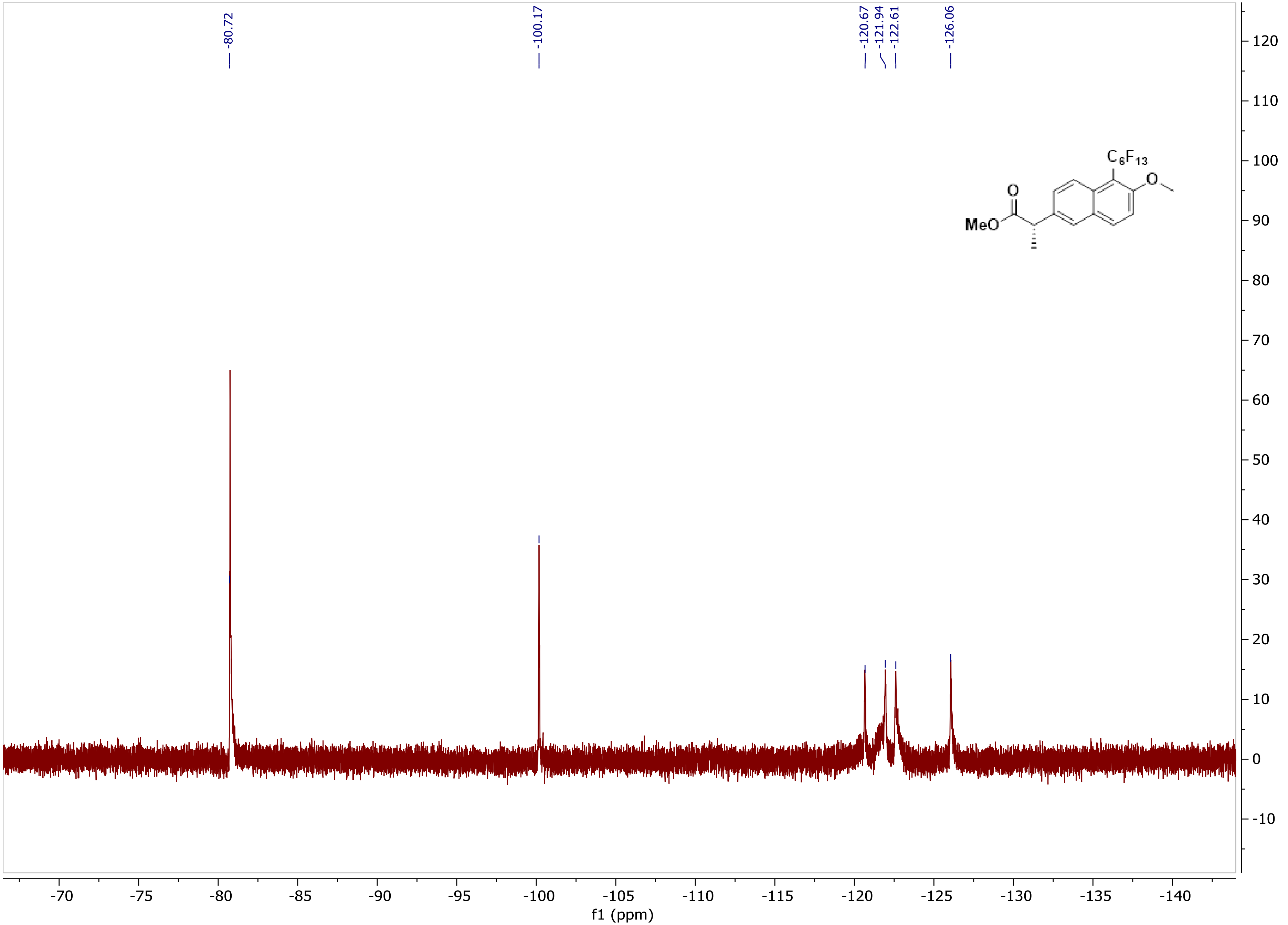

^{1}H-^{19}F HMBC of **14b**

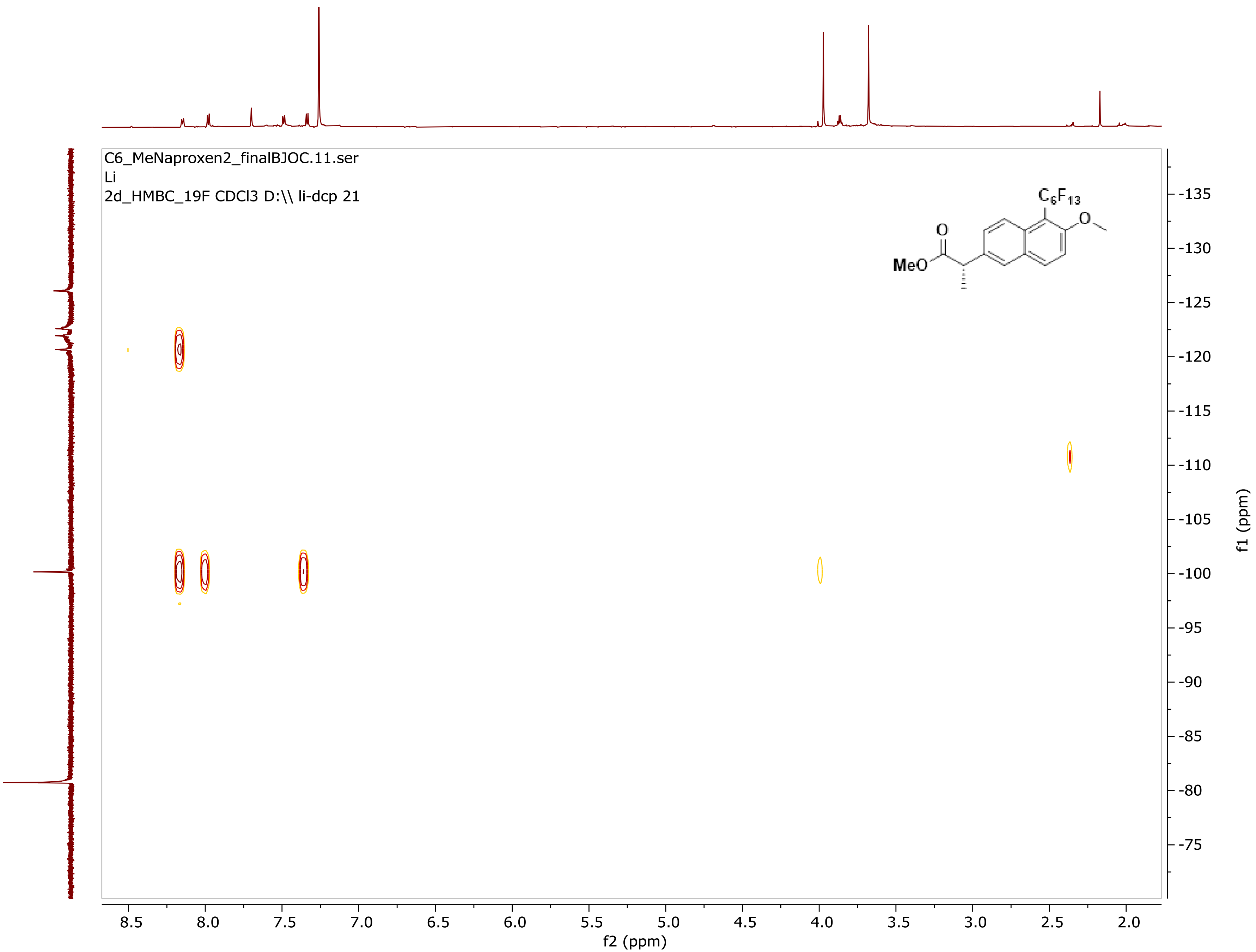

^{1}H NMR of **15b**, acetone-d_6, 500 MHz

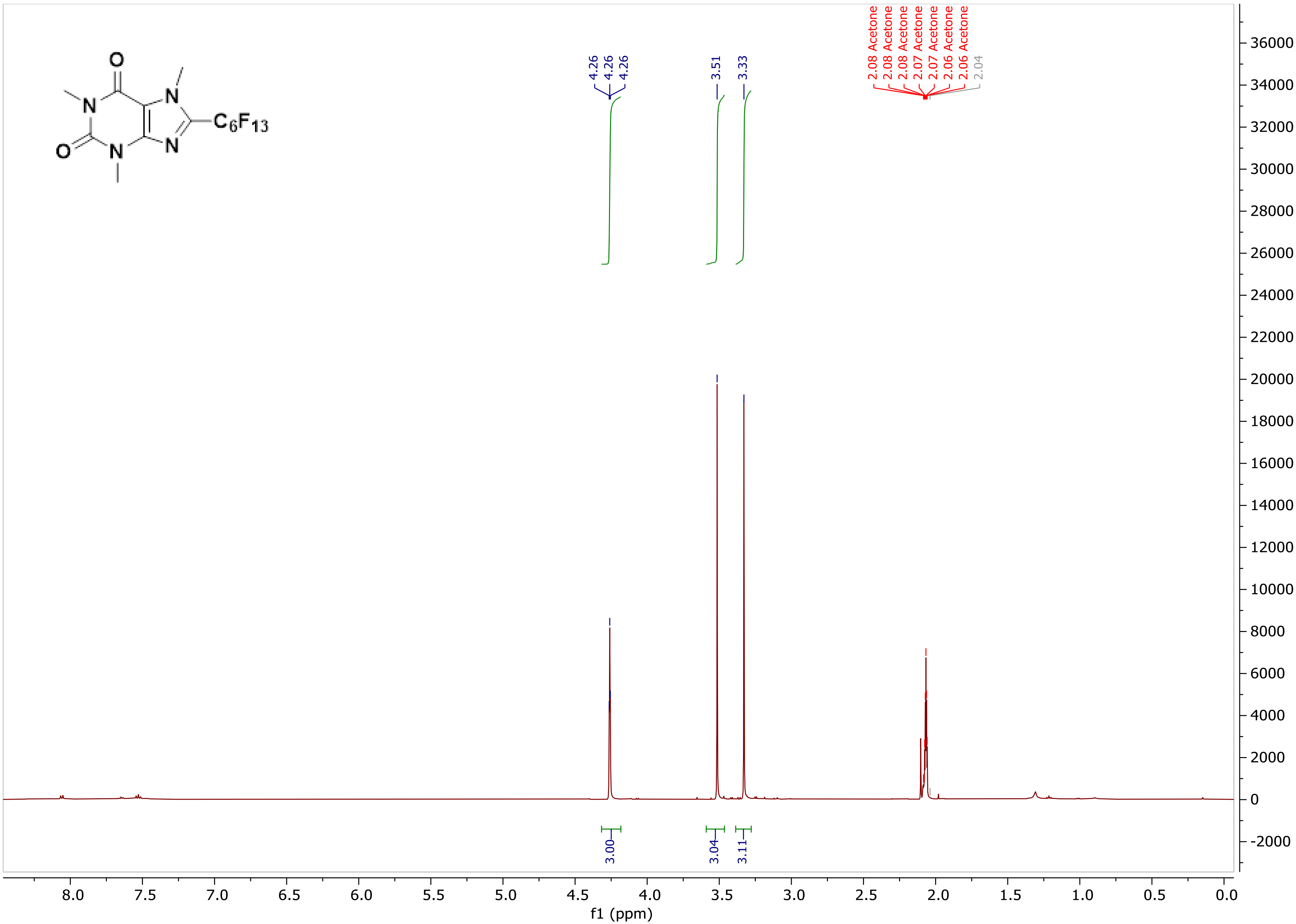

^{13}C NMR of **15b**, acetone-d_6, 126 MHz

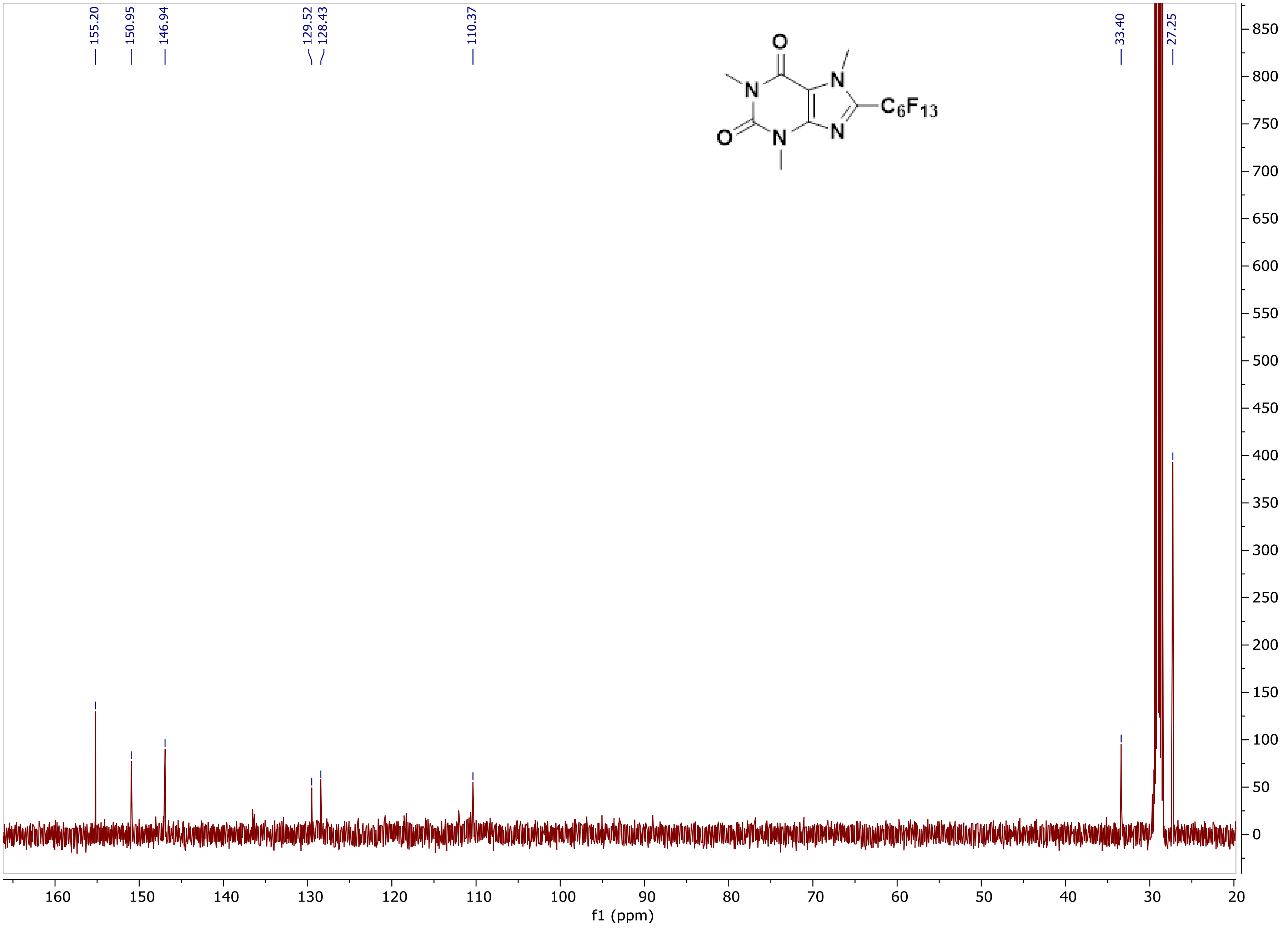

^{19}F NMR of **15b**, acetone-d_6, 471 MHz

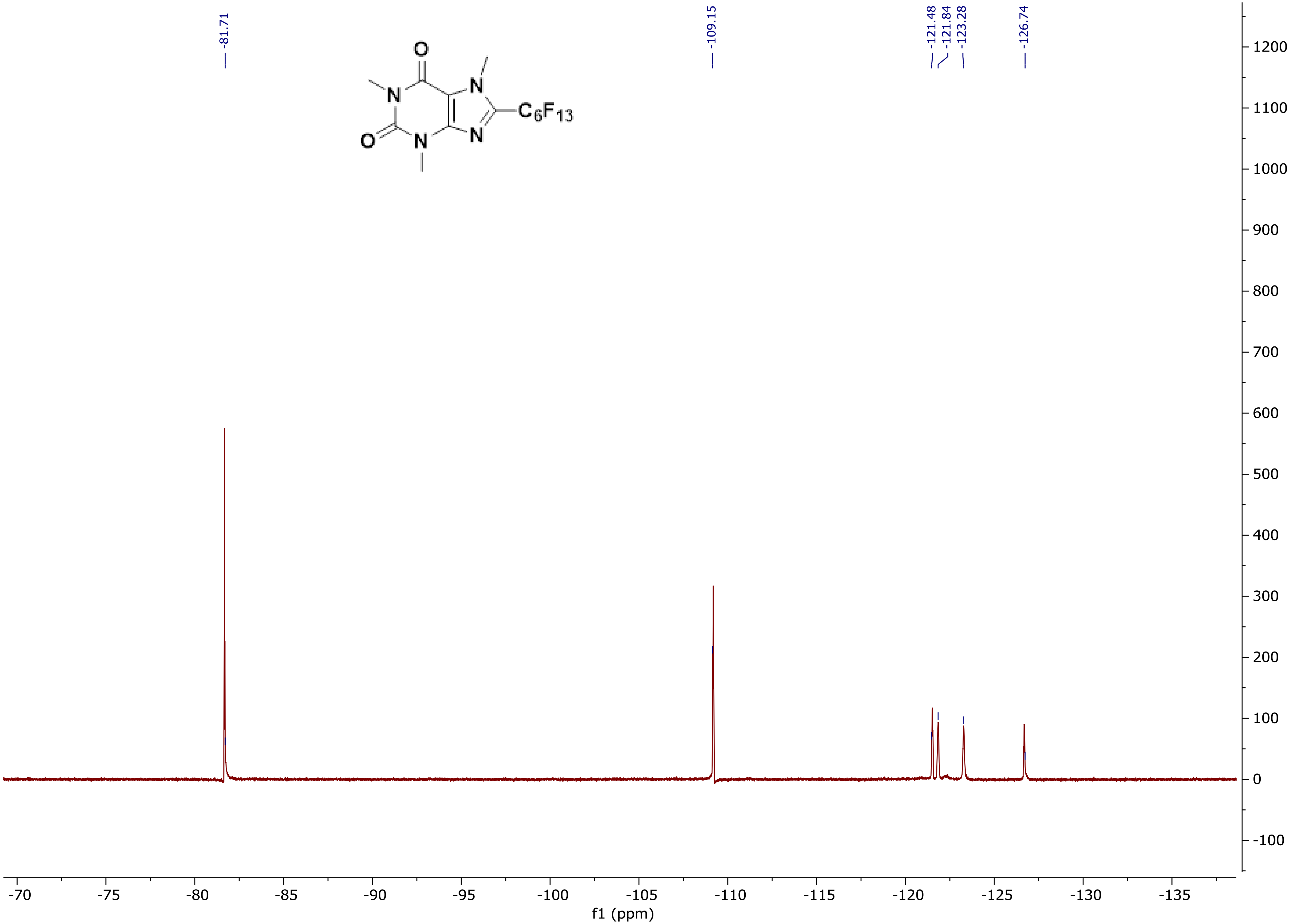

^{1}H NMR of **15c**, acetone-d_6, 500 MHz

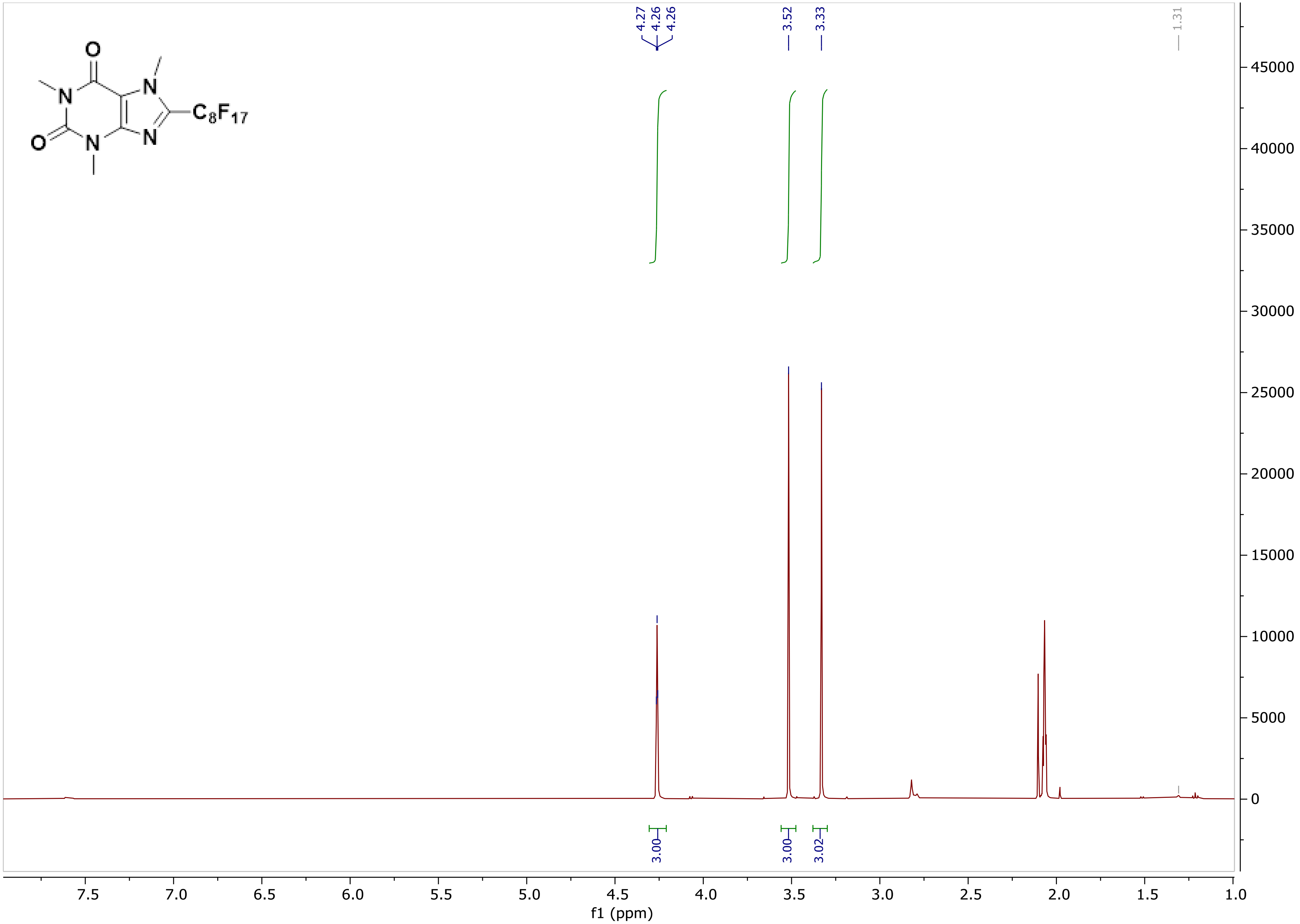

^{13}C NMR of **15c**, acetone-d_6, 126 MHz

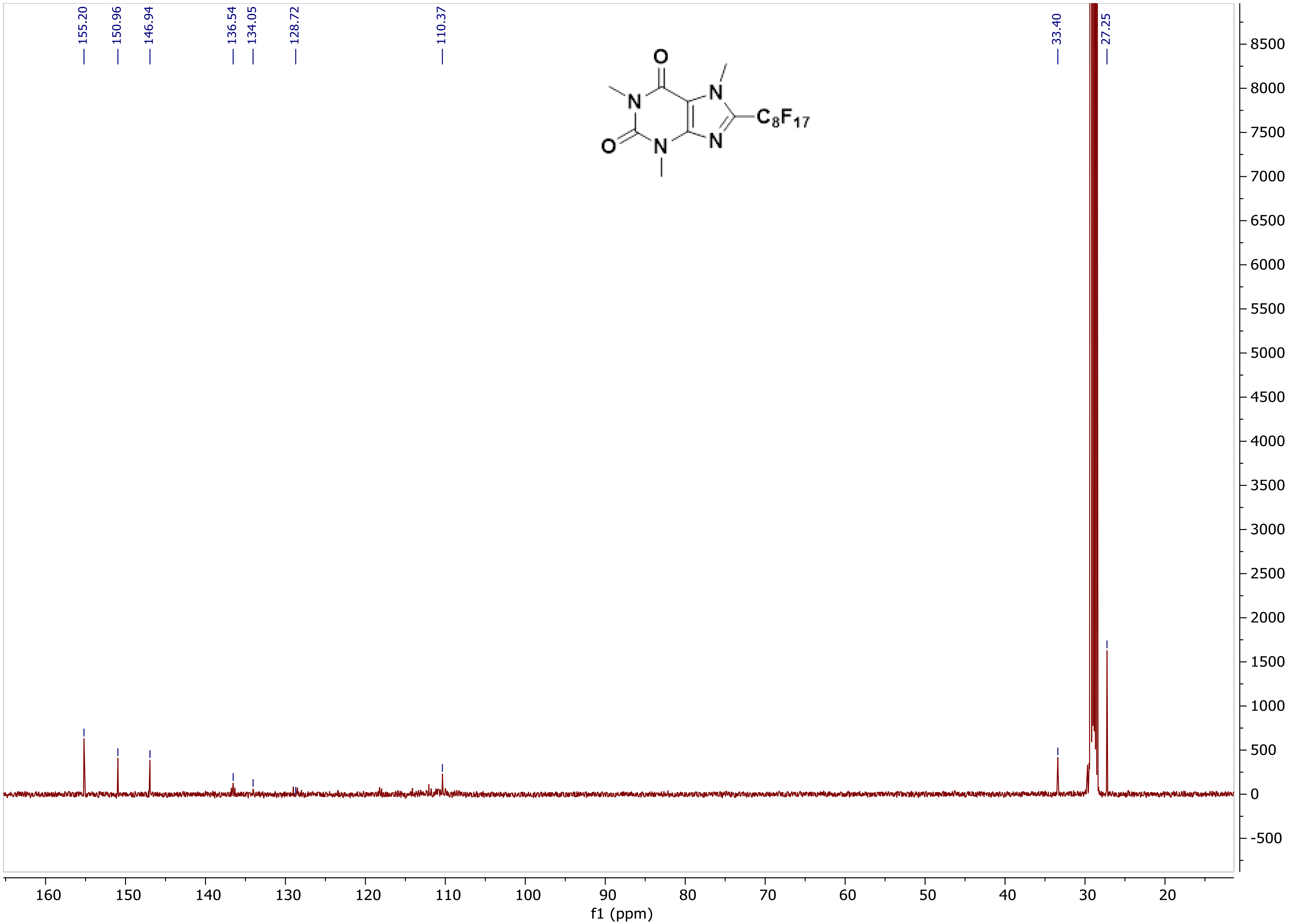

^{19}F NMR of **15c**, acetone-d_6, 471 MHz

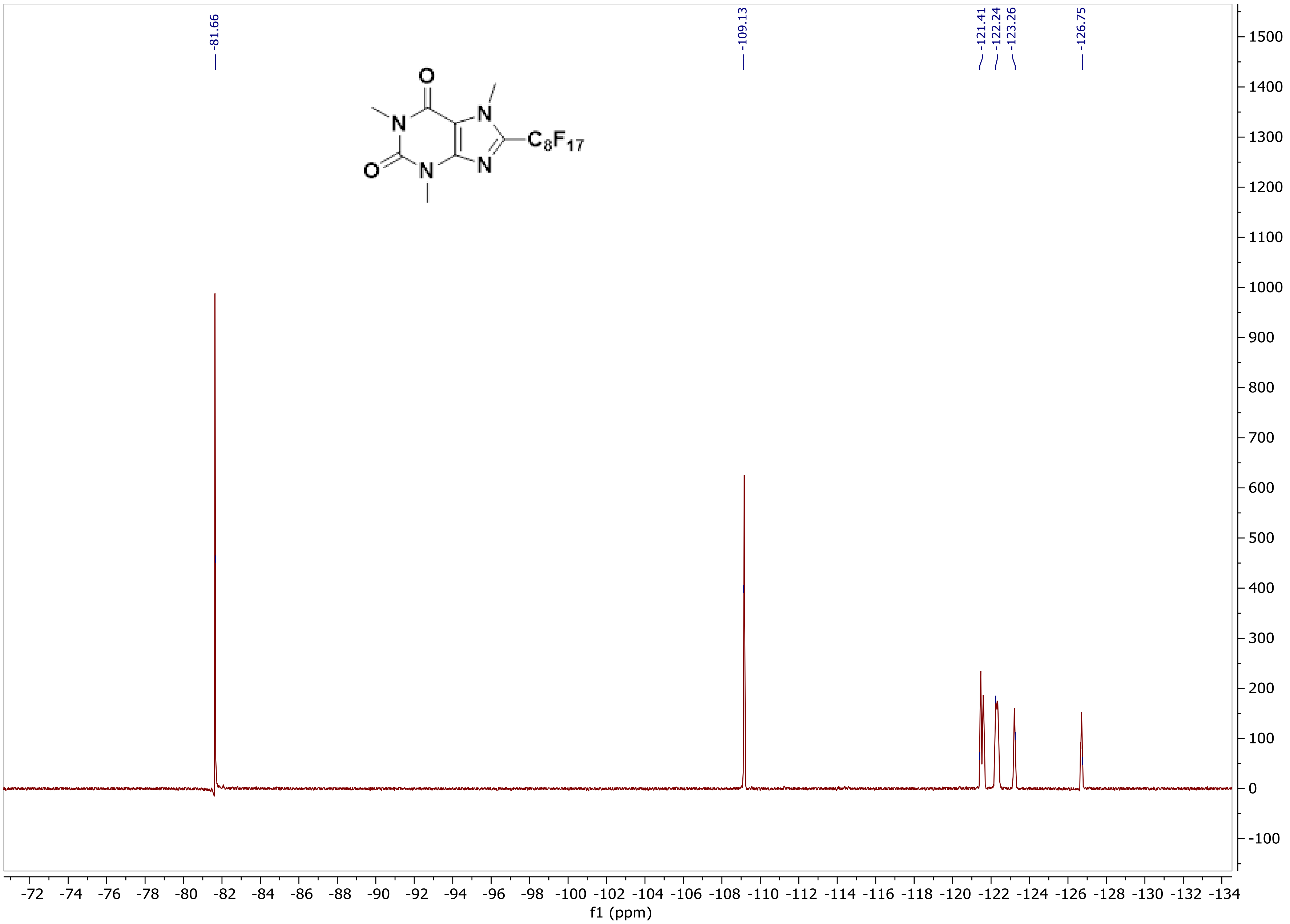

^{1}H NMR of byproduct B (perfluorooctyl analogue), acetone-d_6, 500 MHz

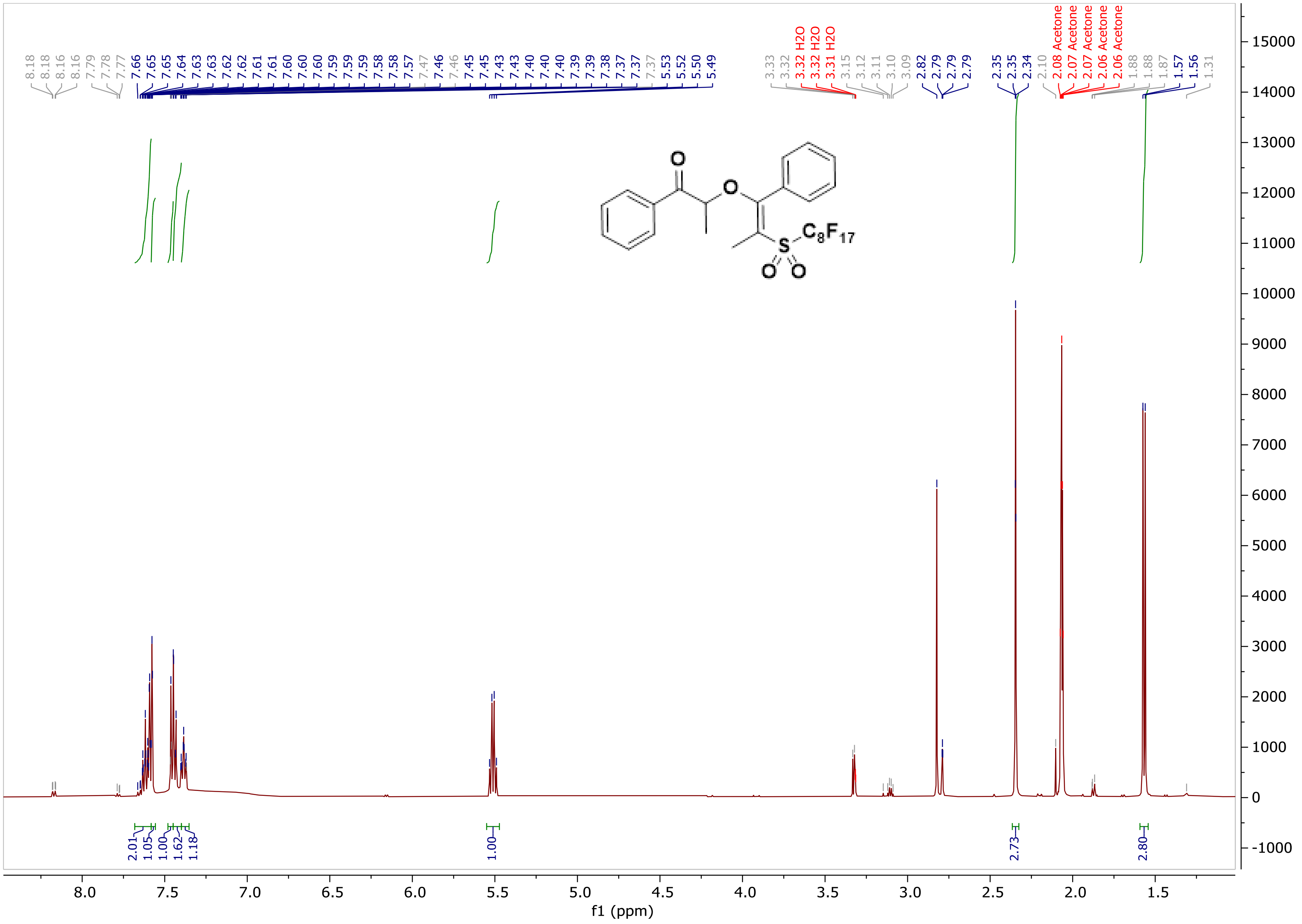

^{13}C NMR of byproduct B (perfluorooctyl analogue), acetone-d_6, 126 MHz

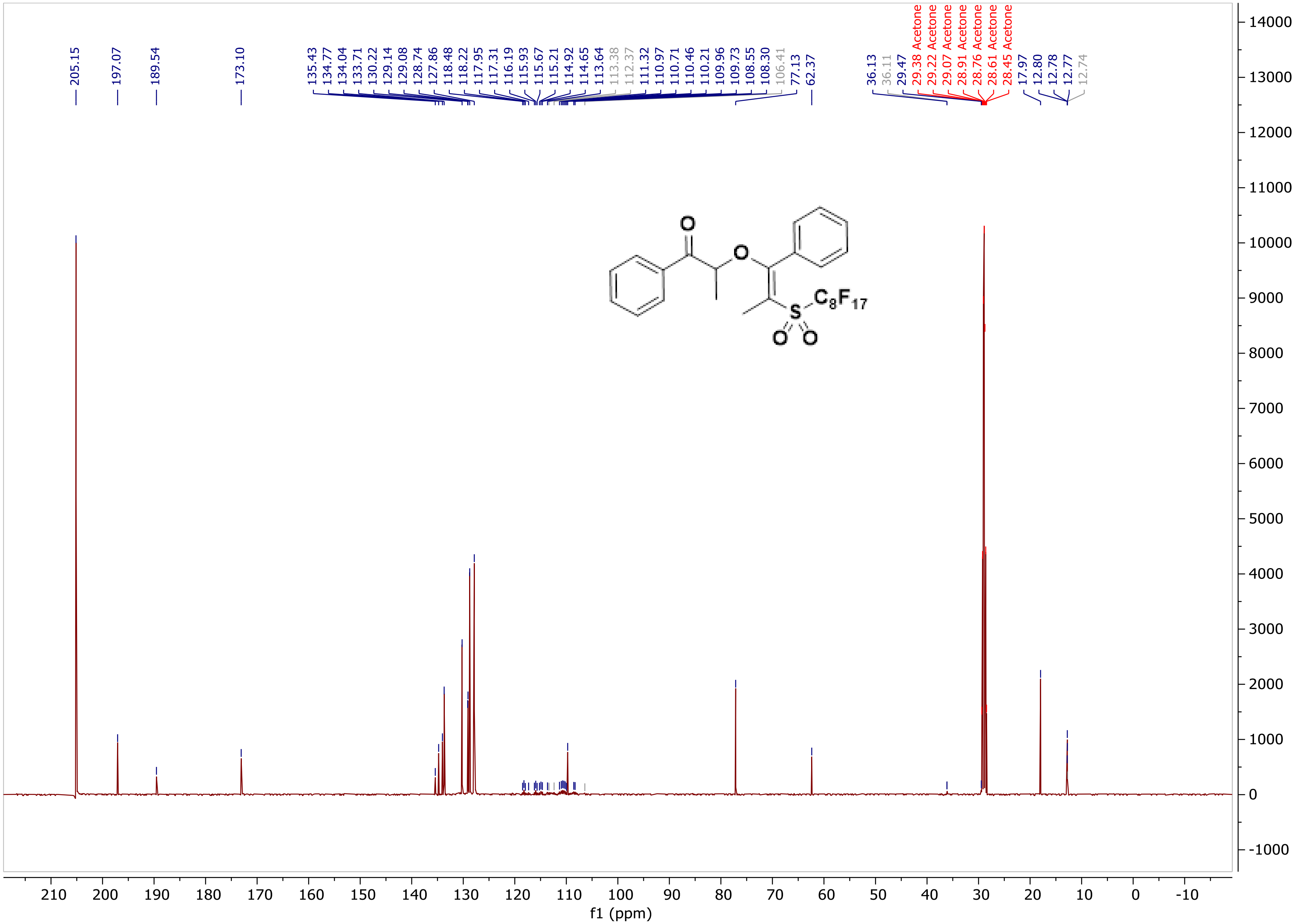

^{19}F NMR of byproduct B (perfluorooctyl analogue), acetone-d_6, 471 MHz

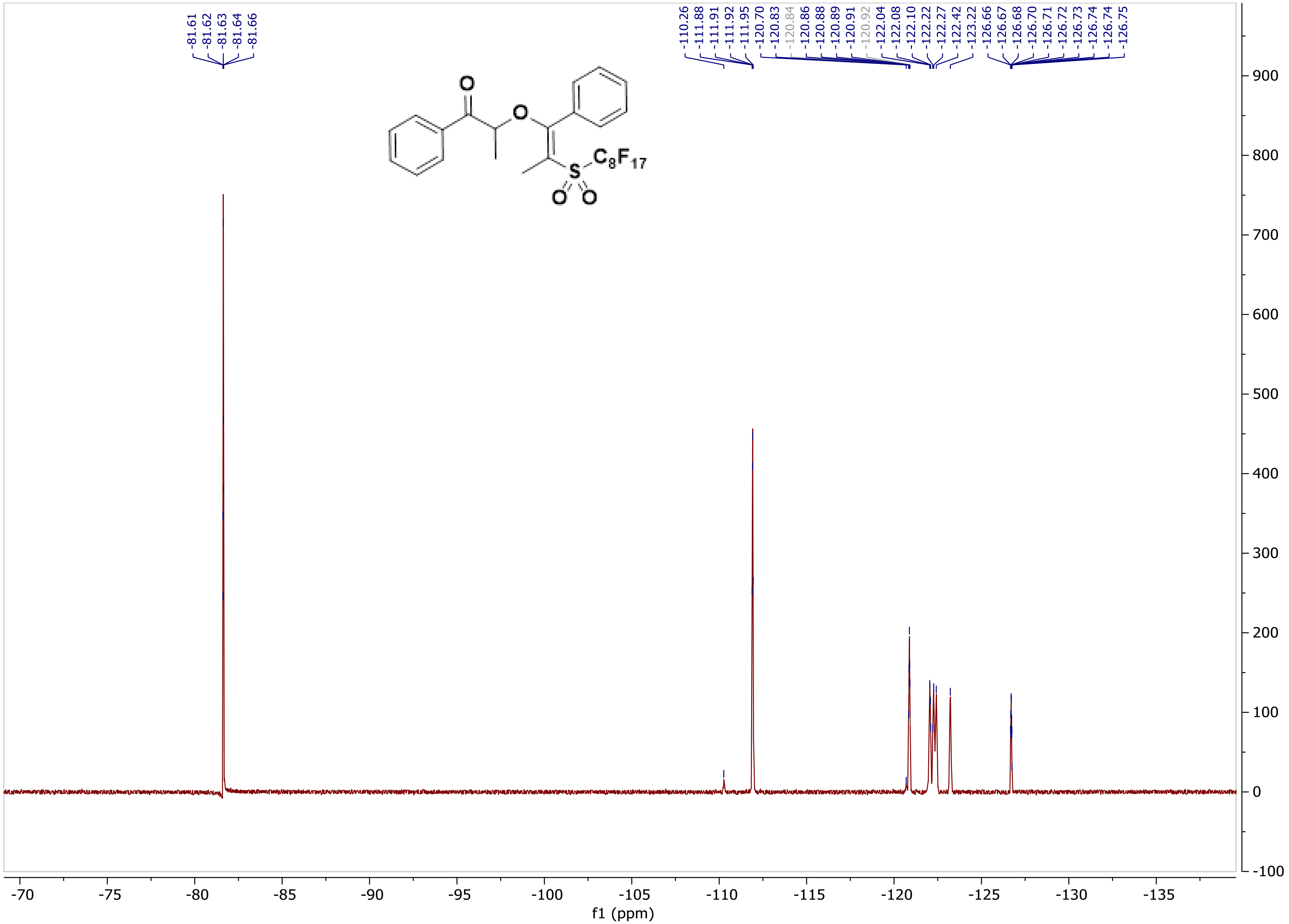

Supplement: File 1 — Detailed experimental procedures and compound characterization data. [file Beilstein_J_Org_Chem-18-788-s001.pdf]
